# Supplementary material for: Hydantoanabaenopeptins from Lake Kinneret Microcystis Bloom, Isolation, and Structure Elucidation of the Possible Intermediates in the Anabaenopeptins Biosynthesis
Source: Mar Drugs. 2023 Jul 13;21(7):401. doi: 10.3390/md21070401 (PMC10381486; doi:10.3390/md21070401)

Hydantoanabaenopeptins from Lake Kinneret *Microcystis* Bloom, Isolation, and Structure Elucidation of the Possible Intermediates in the Anabaenopeptins Biosynthesis

Supplementary Material

Table of Content

Page	Titel
S2-S5	Table of Content
S6	Figure S1. ^{1}H NMR Spectrum of Hydanto-anabaenopeptin A (**1**) in DMSO-d_6
S7	Figure S2. ^{13}C NMR Spectrum of Hydanto-anabaenopeptin A (**1**) in DMSO-d_6
S8	Figure S3. HSQC Spectrum of Hydanto-anabaenopeptin A (**1**) in DMSO-d_6
S9	Figure S4. HMBC Spectrum of Hydanto-anabaenopeptin A (**1**) in DMSO-d_6
S10	Figure S5. COSY Spectrum of Hydanto-anabaenopeptin A (**1**) in DMSO-d_6
S11	Figure S6. TOCSY Spectrum of Hydanto-anabaenopeptin A (**1**) in DMSO-d_6
S12	Figure S7. ROESY Spectrum of Hydanto-anabaenopeptin A (**1**) in DMSO-d_6
S13	Figure S8. Negative HR ESI MS of Hydanto-anabaenopeptin A (**1**)
S14	Figure S9. Positive HR ESI MS/MS Spectrum of Hydanto-anabaenopeptin A (**1**)
S15	Figure S10. Scheme of Fragmentation in the Positive HR ESI MS/MS Spectrum of Hydanto-anabaenopeptin A (**1**)
S16	Figure S11. LCMS Traces of Marfey's Method Amino Acids Analysis of Hydanto-anabaenopeptin A (**1**)
S17	Table S1. NMR Data of Hydanto-anabaenopeptin A (**1**) in DMSO-d_6
S21	Figure S12. LCMS Chromatogram and Mass spectrum of Hydanto-anabaenopeptin A (**1**)
S22	Figure S13. ^{1}H NMR Spectrum of Hydanto-anabaenopeptin B (**2**) in DMSO-d_6
S23	Figure S14. ^{13}C NMR Spectrum of Hydanto-anabaenopeptin B (**2**) in DMSO-d_6
S24	Figure S15. HSQC Spectrum of Hydanto-anabaenopeptin B (**2**) in DMSO-d_6
S25	Figure S16. HMBC Spectrum of Hydanto-anabaenopeptin B (**2**) in DMSO-d_6
S26	Figure S17. COSY Spectrum of Hydanto-anabaenopeptin B (**2**) in DMSO-d_6
S27	Figure S18. TOCSY Spectrum of Hydanto-anabaenopeptin B (**2**) in DMSO-d_6
S28	Figure S19. ROESY Spectrum of Hydanto-anabaenopeptin B (**2**) in DMSO-d_6
S29	Figure S20. Negative HR ESI MS of Hydanto-anabaenopeptin B (**2**)
S30	Figure S21. Positive HR ESI MS/MS Spectrum of Hydanto-anabaenopeptin B (**2**)
S31	Figure S22. Scheme of Fragmentation in the Positive HR ESI MS/MS Spectrum of Hydanto-anabaenopeptin B (**2**)
S32	Figure S23. LCMS Traces of Marfey's Method Amino Acids Analysis of Hydanto-anabaenopeptin B (**2**)
S33	Table S2. NMR Data of Hydanto-anabaenopeptin B (**2**) in DMSO-d_6
S36	Figure S24. LCMS Chromatogram and Mass spectrum of Hydanto-anabaenopeptin B (**2**)
S37	Figure S25. ^{1}H NMR Spectrum of Hydanto-anabaenopeptin F (**3**) in DMSO-d_6
S38	Figure S26. ^{13}C NMR Spectrum of Hydanto-anabaenopeptin F (**3**) in DMSO-d_6

S39	Figure S27. HSQC Spectrum of Hydanto-anabaenopeptin F (**3**) in DMSO-d_6
S40	Figure S28. HMBC Spectrum of Hydanto-anabaenopeptin F (**3**) in DMSO-d_6
S41	Figure S29. COSY Spectrum of Hydanto-anabaenopeptin F (**3**) in DMSO-d_6
S42	Figure S30. TOCSY Spectrum of Hydanto-anabaenopeptin F (**3**) in DMSO-d_6
S43	Figure S31. ROESY Spectrum of Hydanto-anabaenopeptin F (**3**) in DMSO-d_6
S44	Figure S32. Negative HR ESI MS of Hydanto-anabaenopeptin F (**3**)
S45	Figure S33. Positive HR ESI MS/MS Spectrum of Hydanto-anabaenopeptin F (**3**)
S46	Figure S34. Scheme of Fragmentation in the Positive HR ESI MS/MS Spectrum of Hydanto-anabaenopeptin F (**3**)
S47	Figure S35. LCMS Traces of Marfey's Method Amino Acids Analysis of Hydanto-anabaenopeptin F (**3**)
S48	Table S3. NMR Data of Hydanto-anabaenopeptin F (**3**) in DMSO-d_6
S52	Figure S36. LCMS Chromatogram and Mass spectrum of Hydanto-anabaenopeptin F (**3**)
S53	Figure S37. ^{1}H NMR Spectrum of Hydanto-oscillamide Y (**4**) in DMSO-d_6
S54	Figure S38. ^{13}C NMR Spectrum of Hydanto-oscillamide Y (**4**) in DMSO-d_6
S55	Figure S39. HSQC Spectrum of Hydanto-oscillamide n Y (**4**) in DMSO-d_6
S56	Figure S40. HMBC Spectrum of Hydanto-oscillamide Y (**4**) in DMSO-d_6
S57	Figure S41. COSY Spectrum of Hydanto-oscillamide Y (**4**) in DMSO-d_6
S58	Figure S42. TOCSY Spectrum of Hydanto-oscillamide Y (**4**) in DMSO-d_6
S59	Figure S43. ROESY Spectrum of Hydanto-oscillamide Y (**4**) in DMSO-d_6
S60	Figure S44. Negative HR ESI MS of Hydanto-oscillamide Y (**4**)
S61	Figure S45. Positive HR ESI MS/MS Spectrum of Hydanto-oscillamide Y (**4**)
S62	Figure S46. Scheme of Fragmentation in the Positive HR ESI MS/MS Spectrum of Hydanto-oscillamide Y (**4**)
S63	Figure S47. LCMS Traces of Marfey's Method Amino Acids Analysis of Hydanto-oscillamide Y (**4**)
S64	Table S4. NMR Data of Hydanto-oscillamide Y (**4**) in DMSO-d_6
S67	Figure S48. LCMS Chromatogram and Mass spectrum of Hydanto-oscillamide Y (**4**)
S68	Figure S49. ^{1}H NMR Spectrum of ^{1}[Dht]-Anabaenopeptin A (**5**) in DMSO-d_6
S69	Figure S50. ^{13}C NMR Spectrum of ^{1}[Dht]-Anabaenopeptin A (**5**) in DMSO-d_6
S70	Figure S51. HSQC Spectrum of ^{1}[Dht]-Anabaenopeptin A (**5**) in DMSO-d_6
S71	Figure S52. HMBC Spectrum of ^{1}[Dht]-Anabaenopeptin A (**5**) in DMSO-d_6
S72	Figure S53. COSY Spectrum of ^{1}[Dht]-Anabaenopeptin A (**5**) in DMSO-d_6
S73	Figure S54. TOCSY Spectrum of ^{1}[Dht]-Anabaenopeptin A (**5**) in DMSO-d_6
S74	Figure S55. ROESY Spectrum of ^{1}[Dht]-Anabaenopeptin A (**5**) in DMSO-d_6
S75	Figure S56. Positive HR ESI MS of ^{1}[Dht]-Anabaenopeptin A (**5**)

S76	Figure S57. Positive HR ESI MS/MS Spectrum of ^{1}[Dht]-Anabaenopeptin A (**5**)
S77	Figure S58. Scheme of Fragmentation in the Positive HR ESI MS/MS Spectrum of ^{1}[Dht]-Anabaenopeptin A (**5**)
S78	Figure S59. LCMS Traces of Marfey's Method Amino Acids Analysis of ^{1}[Dht]-Anabaenopeptin A (**5**)
S79	Table S5. NMR Data of ^{1}[Dht]-Anabaenopeptin A (**5**) in DMSO-d_6
S82	Figure S60. LCMS Chromatogram and Mass spectrum of ^{1}[Dht]-Anabaenopeptin A (**5**)
S83	Figure S61. ^{1}H NMR Spectrum of Hydanto-^{1}[Dht]-anabaenopeptin A (**6**) in DMSO-d_6
S84	Figure S62. ^{13}C NMR Spectrum of Hydanto-^{1}[Dht]-anabaenopeptin A (**6**) in DMSO-d_6
S85	Figure S63. HSQC Spectrum of Hydanto-^{1}[Dht]-anabaenopeptin A (**6**) in DMSO-d_6
S86	Figure S64. HMBC Spectrum of Hydanto-^{1}[Dht]-anabaenopeptin A (**6**) in DMSO-d_6
S87	Figure S65. COSY Spectrum of Hydanto-^{1}[Dht]-anabaenopeptin A (**6**) in DMSO-d_6
S88	Figure S66. TOCSY Spectrum of Hydanto-^{6}[Dht]-anabaenopeptin A (**6**) in DMSO-d_6
S89	Figure S67. ROESY Spectrum of Hydanto-^{1}[Dht]-anabaenopeptin A (**6**) in DMSO-d_6
S90	Figure S68. Positive HR ESI MS of Hydanto-^{1}[Dht]-anabaenopeptin A (**6**)
S91	Figure S69. Positive HR ESI MS/MS Spectrum of Hydanto-^{1}[Dht]-anabaenopeptin (**6**)
S92	Figure S70. Scheme of Fragmentation in the Positive HR ESI MS/MS Spectrum of Hydanto-^{1}[Dht]-anabaenopeptin (**6**)
S93	Figure S71. LCMS Traces of Marfey's Method Amino Acids Analysis of Hydanto-^{1}[Dht]-anabaenopeptin (**6**)
S94	Table S6. NMR Data of Hydanto-^{1}[Dht]-anabaenopeptin A (**6**) in DMSO-d_6
S97	Figure S72. LCMS Chromatogram and Mass spectrum of Hydanto-^{1}[Dht]-anabaenopeptin A (**6**)
S98	Figure S73. ^{1}H NMR Spectrum of Anabaenopeptin A (**7**) in DMSO-d_6
S99	Figure S74. ^{13}C NMR Spectrum of Anabaenopeptin A (**7**) in DMSO-d_6
S100	Figure S75. Negative HR ESI MS of Anabaenopeptin A (**7**)
S101	Figure S76. Positive HR ESI MS/MS Spectrum of Anabaenopeptin A (**7**)
S102	Figure S77. Scheme of Fragmentation in the Positive HR ESI MS/MS Spectrum of Anabaenopeptin A (**7**)
S103	Figure S78. LCMS Traces of Marfey's Method Amino Acids Analysis of Anabaenopeptin A (**7**)
S104	Figure S79. LCMS Chromatogram and Mass spectrum of Anabaenopeptin A (**7**)
S105	Figure S80. ^{1}H NMR Spectrum of Anabaenopeptin B (**8**) in DMSO-d_6
S106	Figure S81. ^{13}C NMR Spectrum of Anabaenopeptin B (**8**) in DMSO-d_6
S107	Figure S82. Positive HR ESI MS of Anabaenopeptin B (**8**)
S108	Figure S83. Positive HR ESI MS/MS Spectrum of Anabaenopeptin B (**8**)
S109	Figure S84. Scheme of Fragmentation in the Positive HR ESI MS/MS Spectrum of Anabaenopeptin B (**8**)
S110	Figure S85. LCMS Traces of Marfey's Method Amino Acids Analysis of Anabaenopeptin B (**8**)
S111	Figure S86. LCMS Chromatogram and Mass spectrum of Anabaenopeptin B (**8**)

S112	Figure S87. ^{1}H NMR Spectrum of Anabaenopeptin F (**9**) in DMSO-d_6
S113	Figure S88. Negative HR ESI MS of Anabaenopeptin F (**9**)
S114	Figure S89. Positive HR ESI MS/MS Spectrum of Anabaenopeptin F (**9**)
S115	Figure S90. Scheme of Fragmentation in the Positive HR ESI MS/MS Spectrum of Anabaenopeptin F (**9**)
S116	Figure S91. LCMS Traces of Marfey's Method Amino Acids Analysis of Anabaenopeptin F (**9**)
S117	Figure S92. LCMS Chromatogram and Mass spectrum of Anabaenopeptin F (**9**)
S118	Figure S93. ^{1}H NMR Spectrum of Oscillamide Y (**10**) in DMSO-d_6
S119	Figure S94. Negative HR ESI MS of Oscillamide Y (**10**)
S120	Figure S95. Positive HR ESI MS/MS Spectrum of Oscillamide Y (**10**)
S121	Figure S96. Scheme of Fragmentation in the Positive HR ESI MS/MS Spectrum of Oscillamide Y (**10**)
S122	Figure S97. LCMS Traces of Marfey's Method Amino Acids Analysis of Oscillamide Y (**10**)
S123	Figure S98. LCMS Chromatogram and Mass spectrum of Oscillamide Y (**10**)

Figure S1. ^{1}H NMR Spectrum of Hydanto-anabaenopeptin A (**1**) in DMSO-d_6

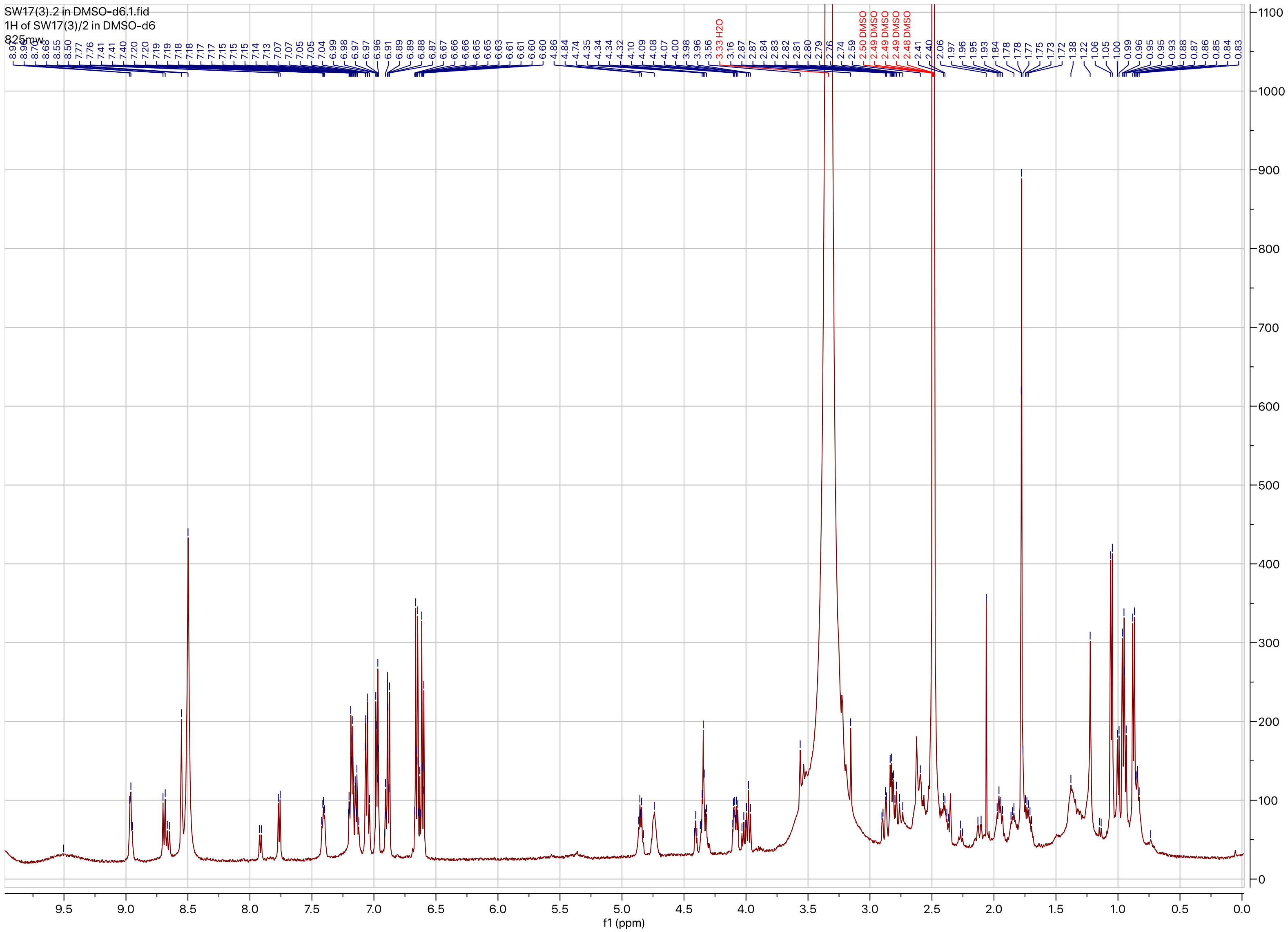

Figure S2. ^{13}C NMR Spectrum of Hydanto-anabaenopeptin A (**1**) in DMSO-d_6

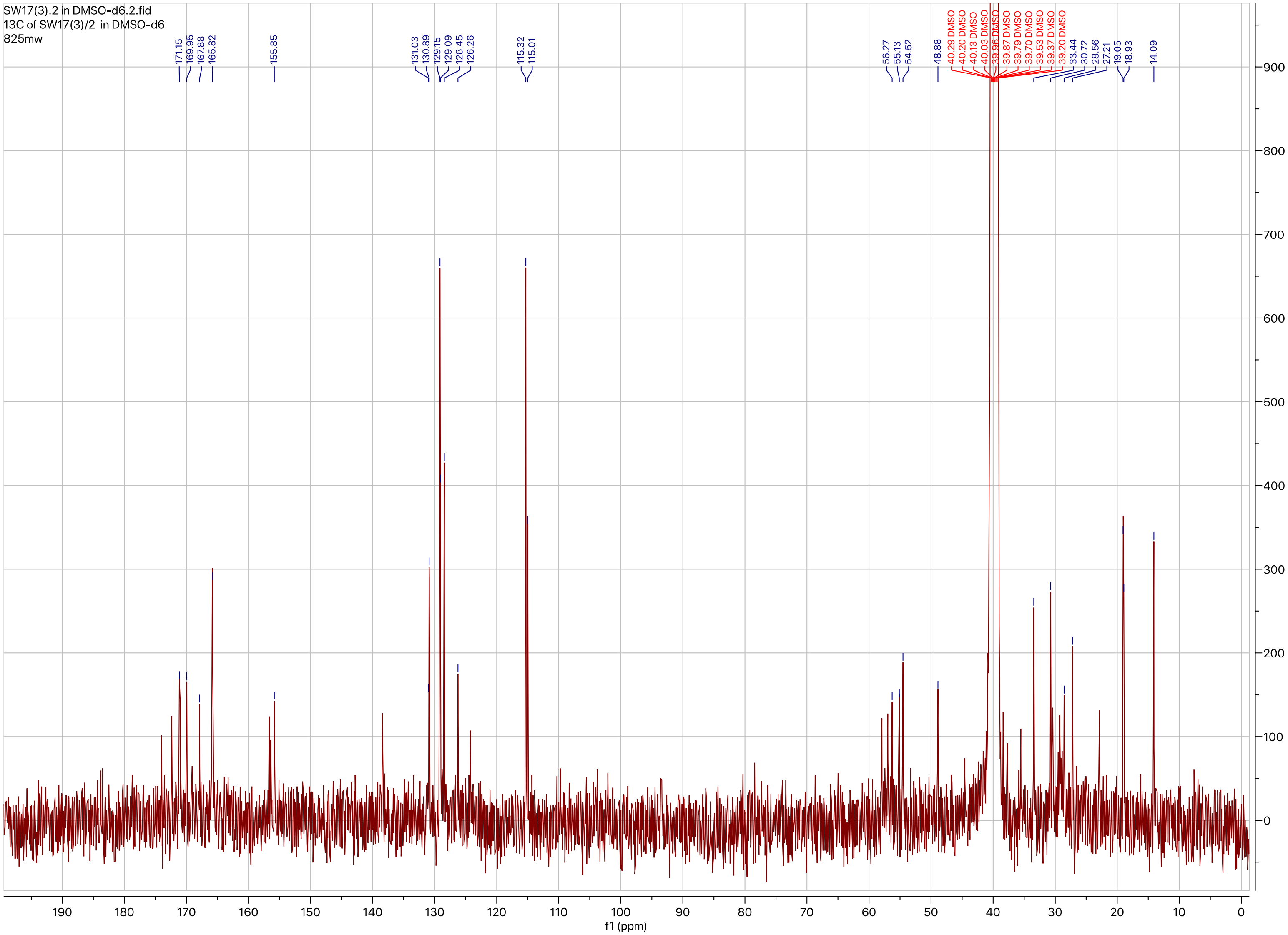

Figure S3. HSQC Spectrum of Hydanto-anabaenopeptin A (**1**) in DMSO-d_6

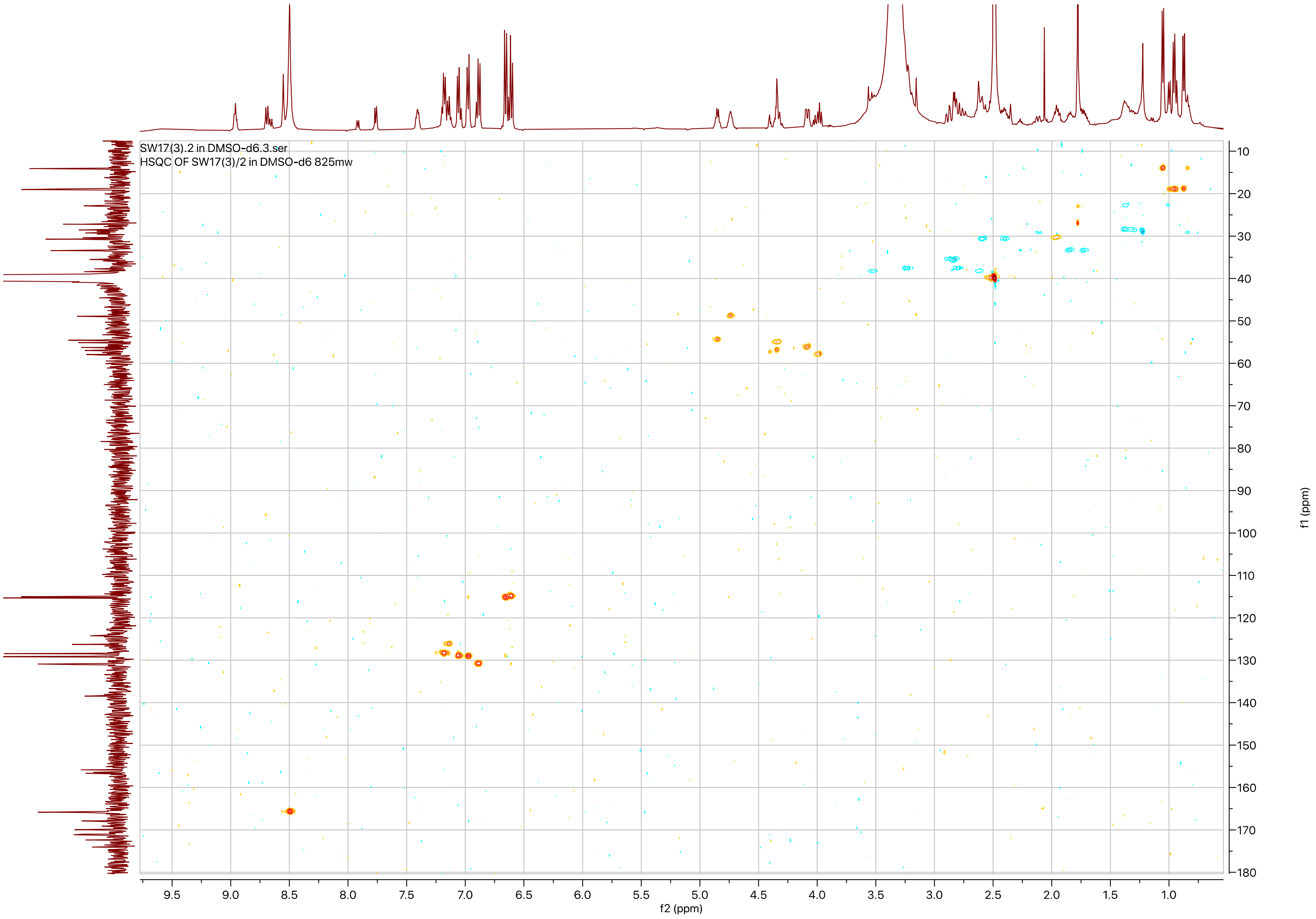

Figure S4. HMBC Spectrum of Hydanto-anabaenopeptin A (**1**) in DMSO-d_6

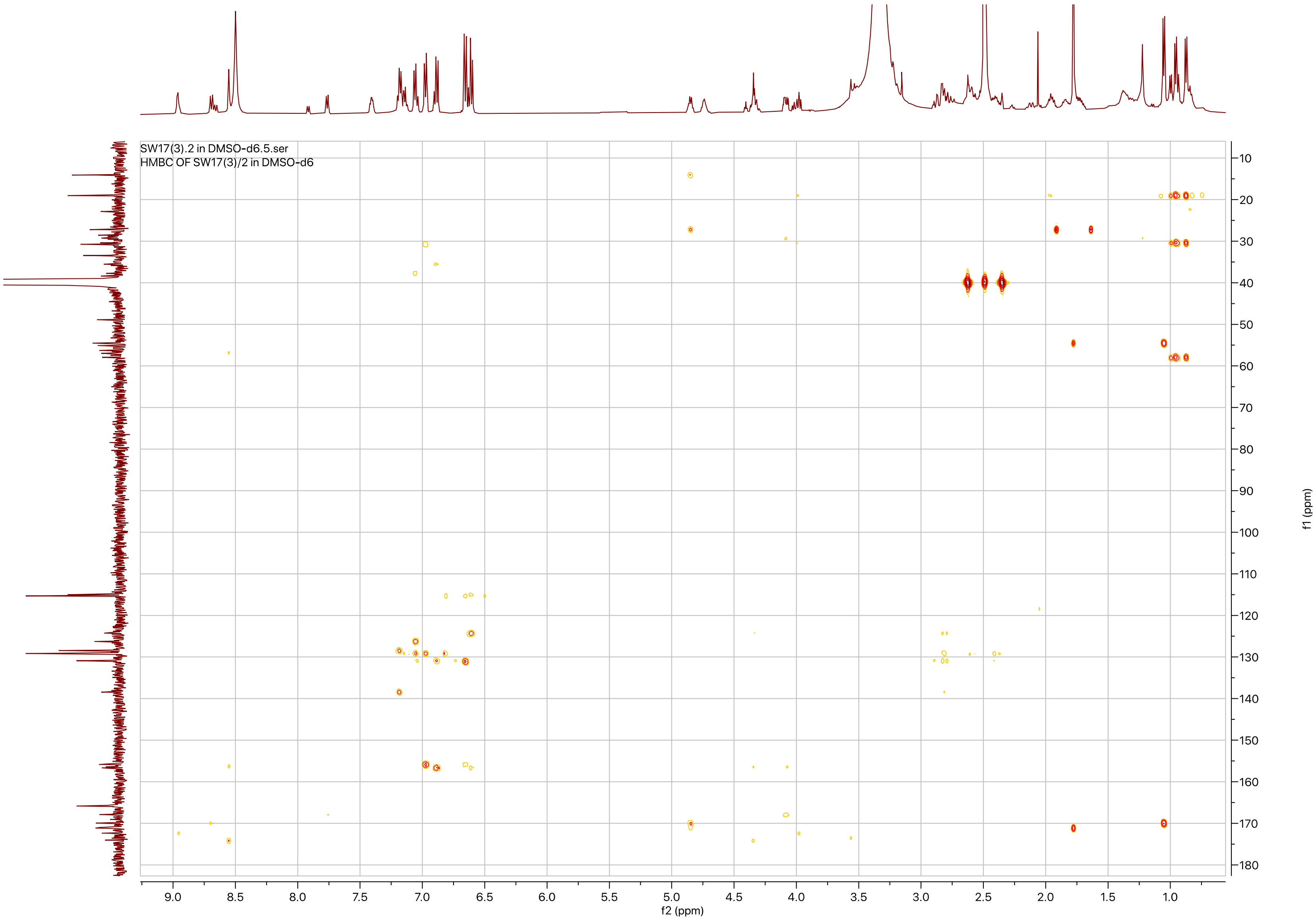

Figure S5. COSY Spectrum of Hydanto-anabaenopeptin A (**1**) in DMSO-d_6

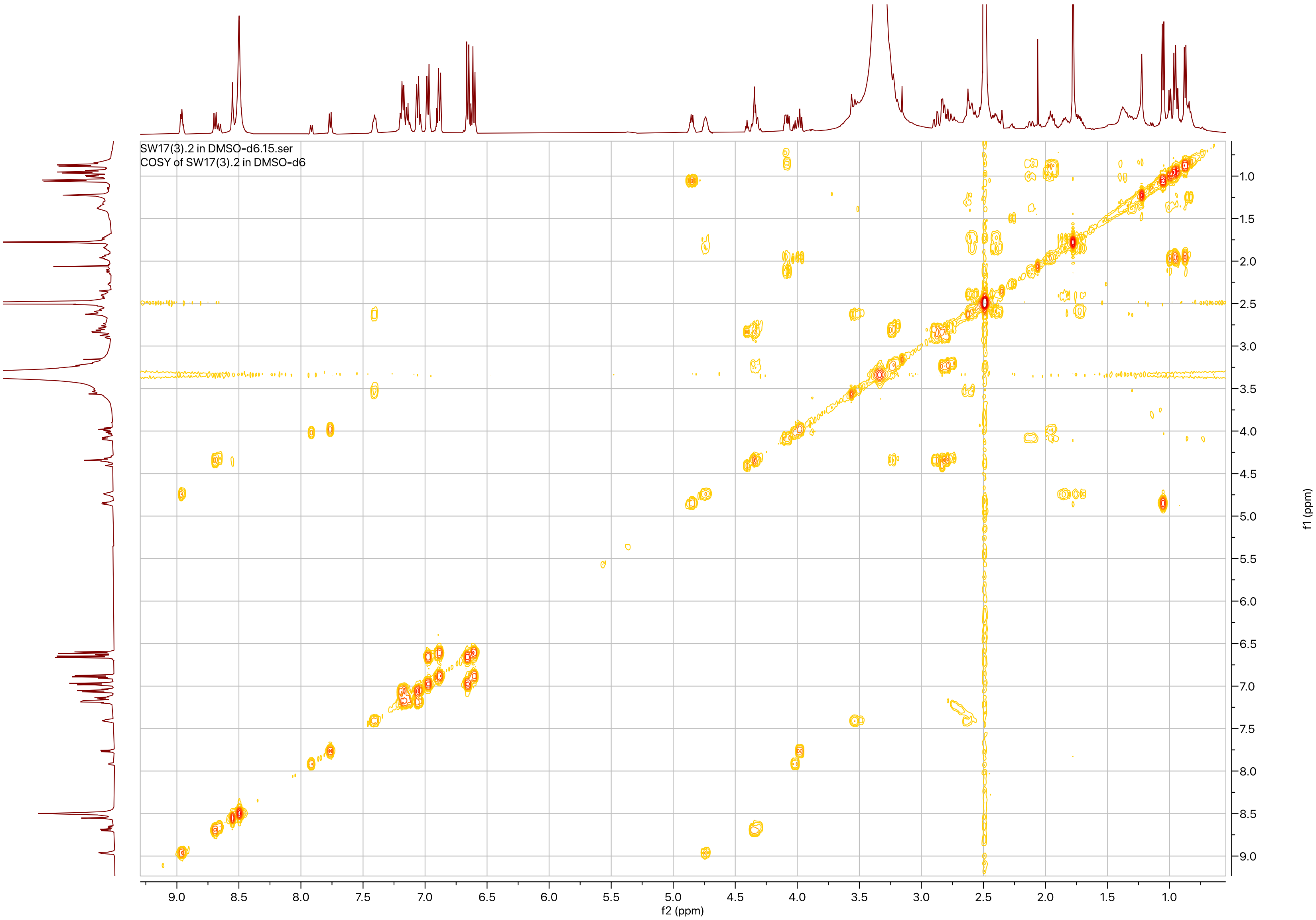

Figure S6. TOCSY Spectrum of Hydanto-anabaenopeptin A (**1**) in DMSO-d_6

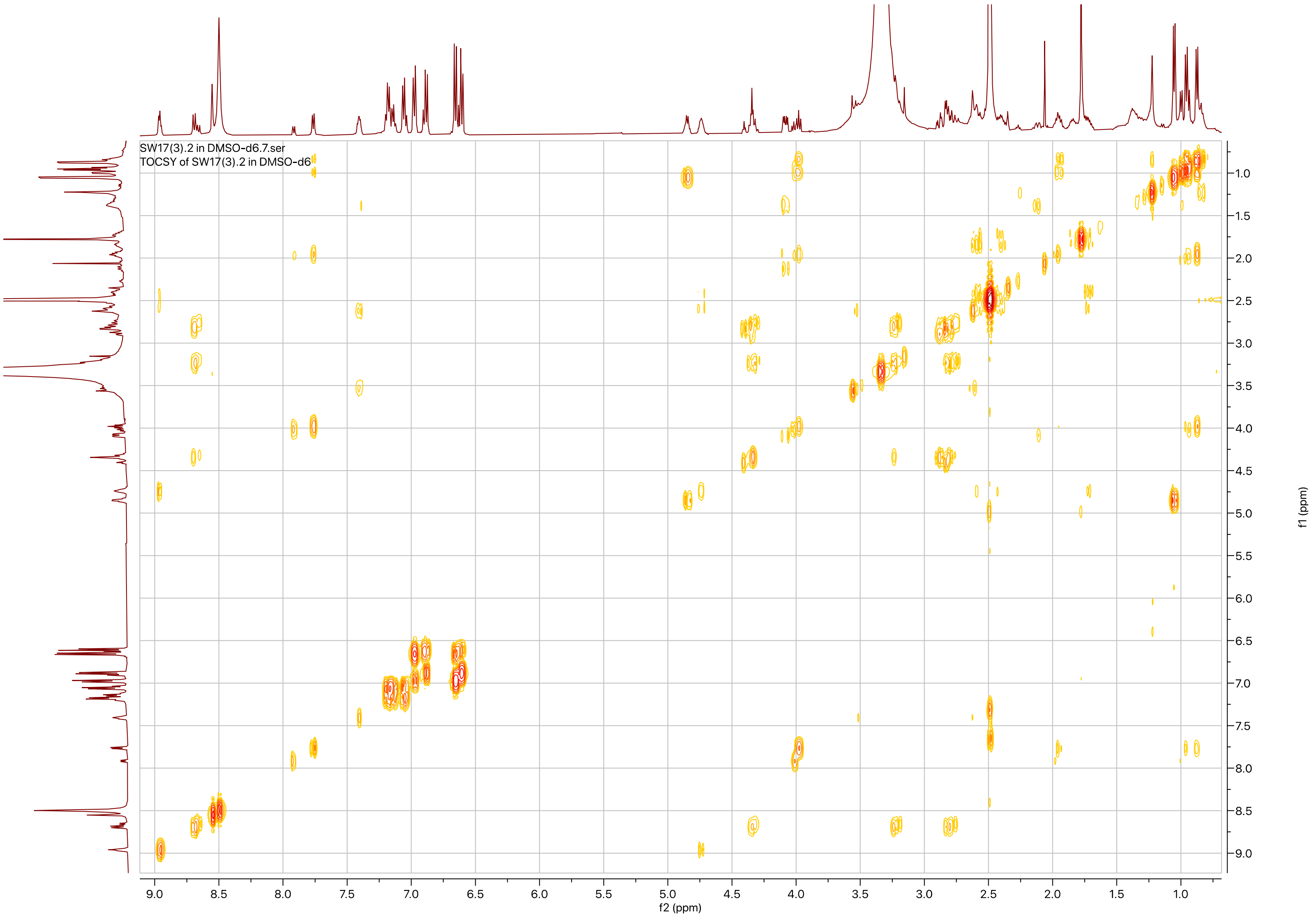

Figure S7. ROESY Spectrum of Hydanto-anabaenopeptin A (**1**) in DMSO-d_6

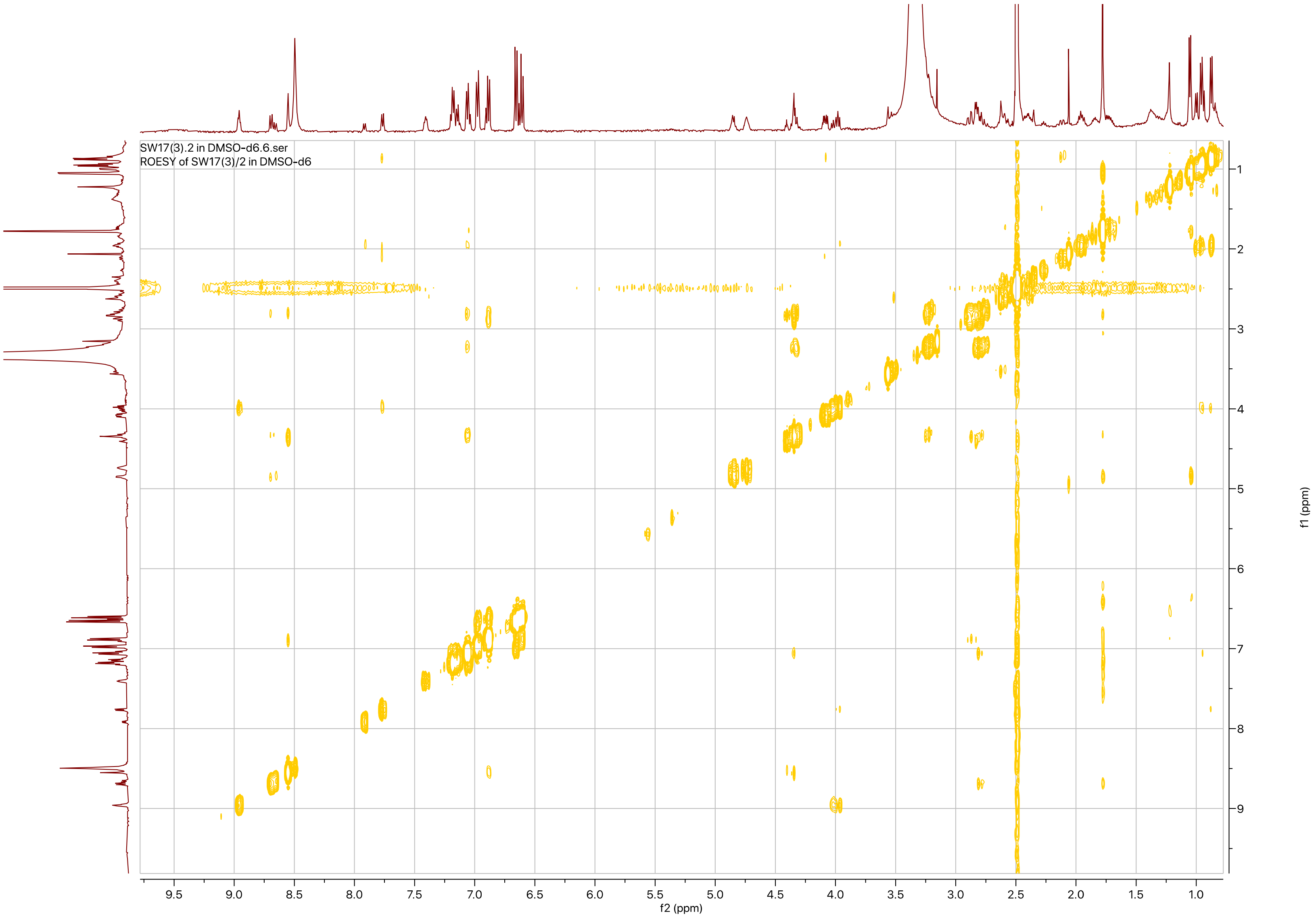

Figure S8. Negative HR ESI MS of Hydanto-anabaenopeptin A (**1**)

Single Mass Analysis
Tolerance = 3.0 PPM / DBE: min = -1.5, max = 50.0
Element prediction: Off
Number of isotope peaks used for i-FIT = 3
Monoisotopic Mass, Odd and Even Electron Ions
570 formula(e) evaluated with 4 results within limits (all results (up to 1000) for each mass)
Elements Used:

Mass	Calc. Mass	mDa	PPM	DBE	Formula	i-FIT	i-FIT Norm	Fit Conf %	C	H	N	O
824.3985	824.3983	0.2	0.2	21.5	C44 H54 N7 O9	586.3	1.335	26.32	44	54	7	9
	824.3983	0.2	0.2	27.0	C43 H48 N14 O4	586.2	1.221	29.48	43	48	14	4
	824.3996	-1.1	-1.3	26.5	C45 H50 N11 O5	588.2	3.232	3.95	45	50	11	5
	824.3970	1.5	1.8	22.0	C42 H52 N10 O8	585.9	0.910	40.25	42	52	10	8

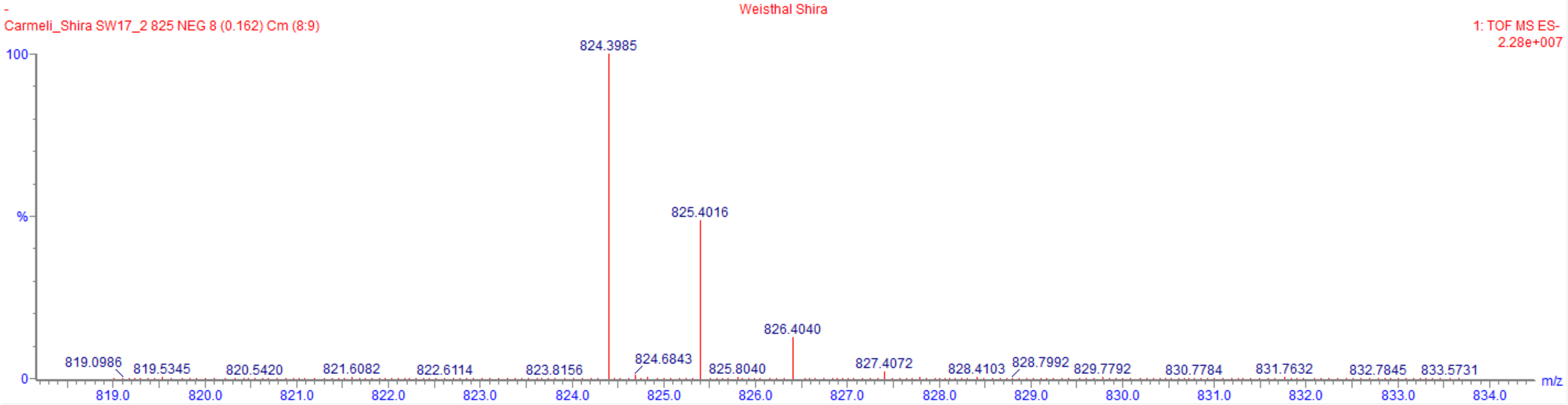

For Help, press F1

Figure S9. Positive HR ESI MS/MS Spectrum of Hydanto-anabaenopeptin A (**1**)

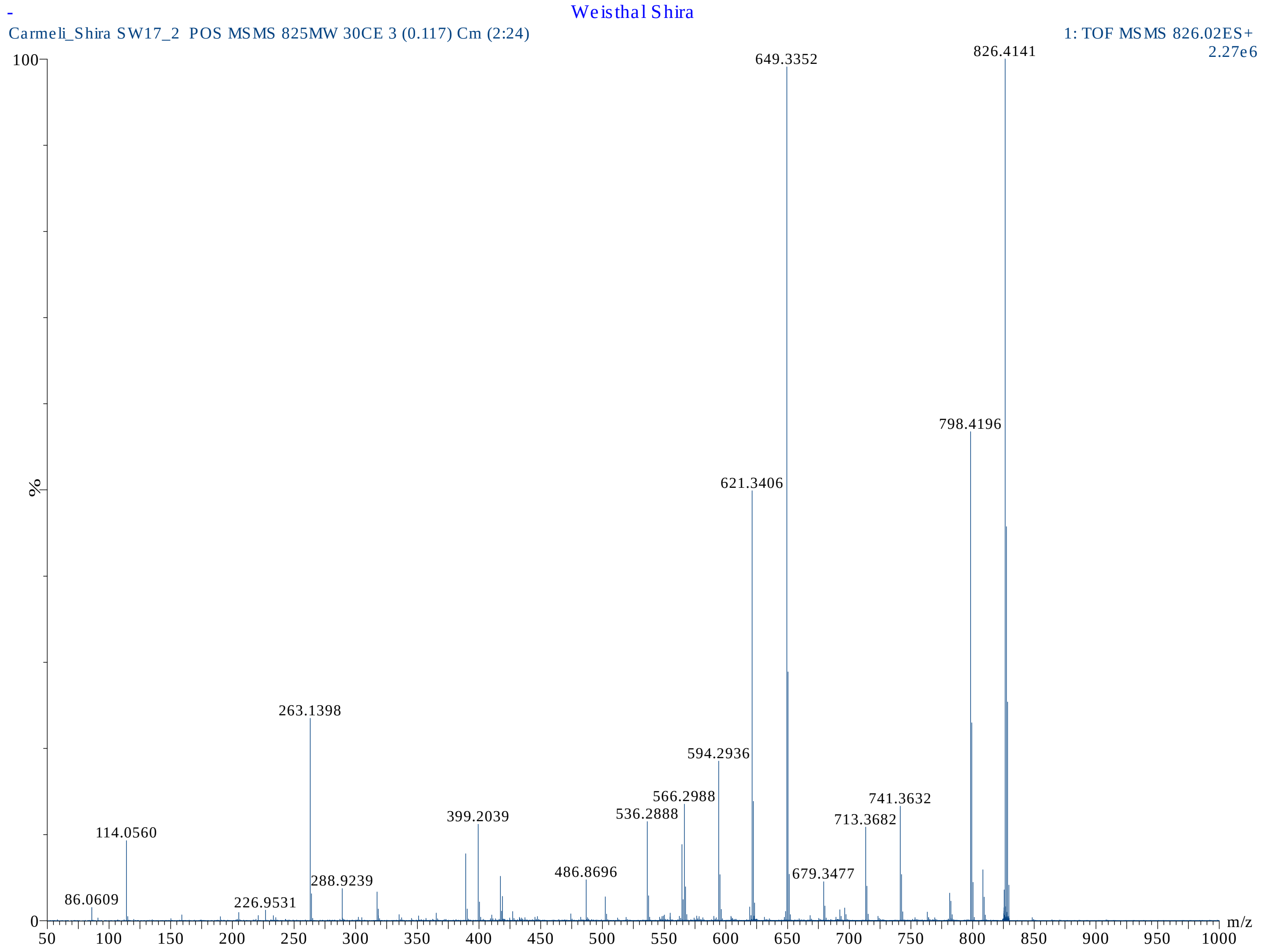

Figure S10. Scheme of Fragmentations in the Positive HR ESI MS/MS Spectrum of Hydanto-anabaenopeptin A (**1**)

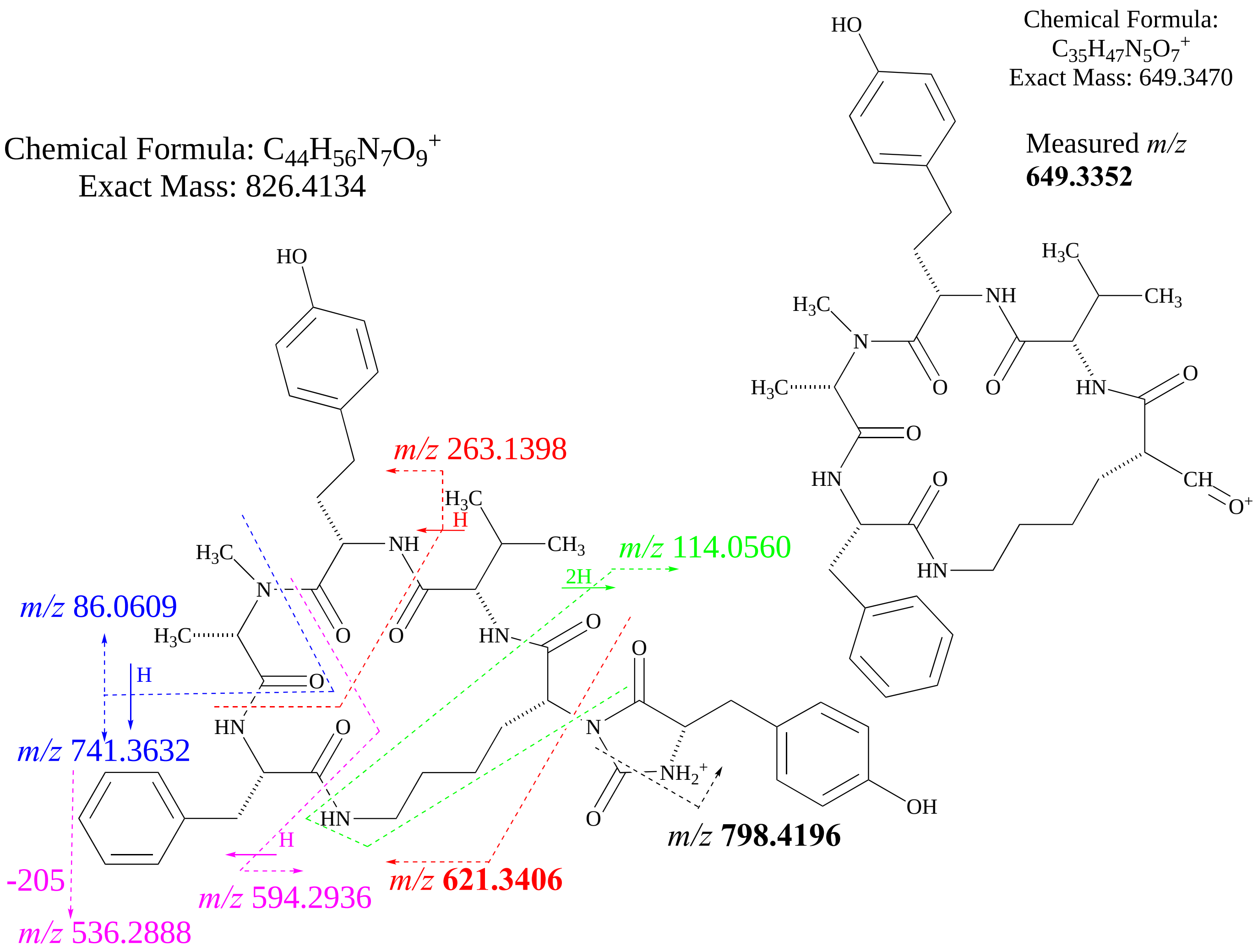

Figure S11. LCMS Traces of Marfey's Method Amino Acids Analysis of Hydanto-anabaenopeptin A (**1**)

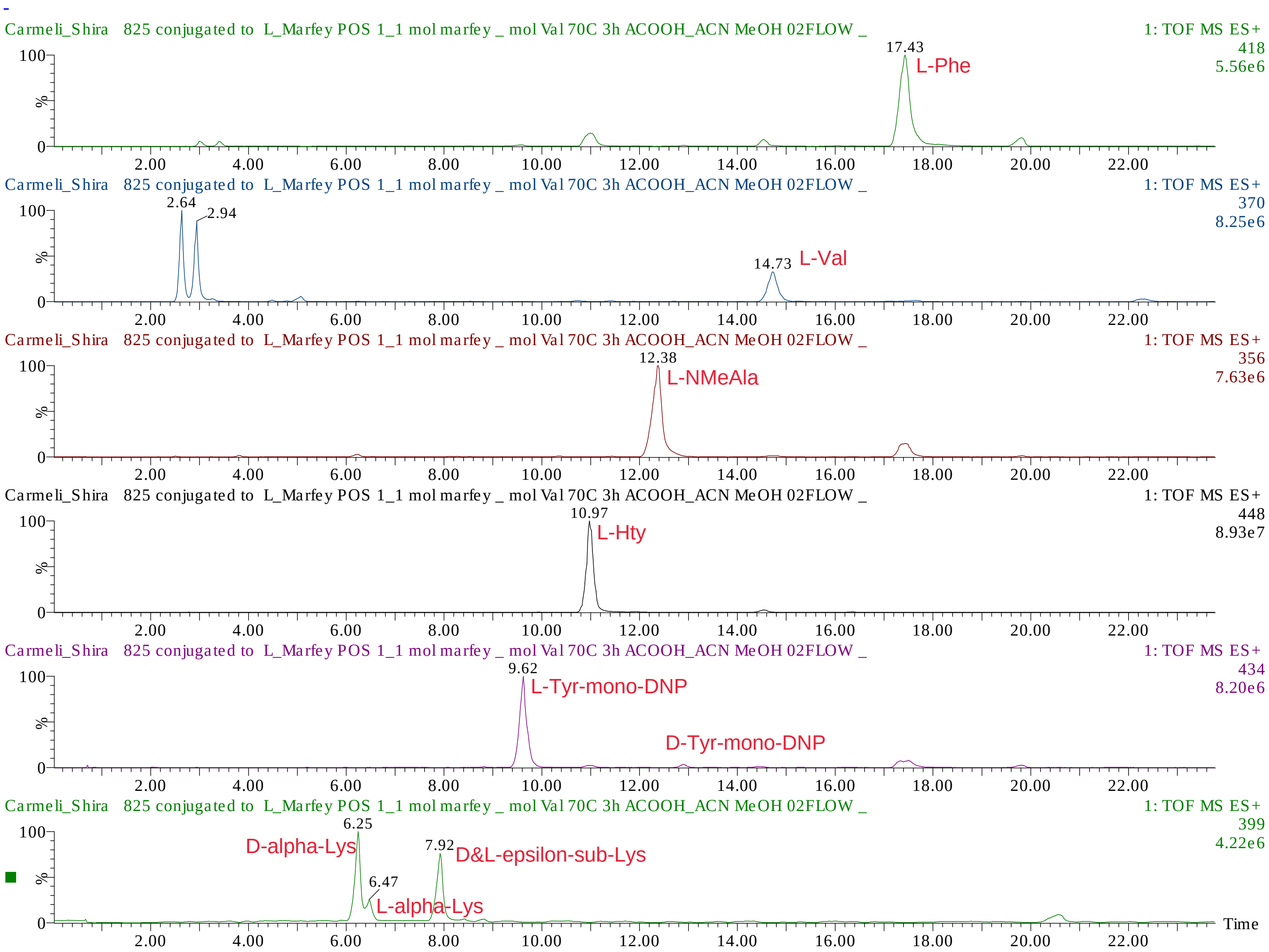

Table S1. NMR Data of Hydanto-anabaenopeptin A (**1**) in DMSO-d_6[a]

Position	δC, Mult.	δH, Mult. (*J* in Hz)[b]	HMBC correlations[c]	COSY correlations	TOCSY correlations	ROESY correlations
Tyr-1	174.1, C		Tyr-2, 3b, 2-NH, Lys-2			
Tyr-2<br><br>*Tyr-2*	57.0, CH<br><br>*57.5, CH*	4.34, t (4.3)<br>*4.40, t (4.4)*	Tyr-3b	Tyr-2-NH, 3a, 3b<br>*Tyr-2-NH, 3b*		Tyr-2-NH, 3b<br>*Tyr-2-NH, 3b*
Tyr-2-NH<br><br>*Tyr-2-NH*		8.55, s<br><br>*8.49, s*			Tyr-2, 3a, 3b<br>*Tyr-2, 3a, 3b*	Tyr-2
Tyr-3a<br><br>Tyr-3b<br>*Tyr-3a*<br>*Tyr-3b*	35.5, CH_2<br><br>*35.8, CH_2*	2.89, dd (14.0,4.0)<br>2.82, m<br>*2.83, m*<br>*2.83, m*	Tyr-5,5’	Tyr-2, 3b<br>Tyr-2, 3a<br><br>*Tyr-2*<br>*Tyr-2*		Tyr-2, 5,5’<br><br><br>*Tyr-2*
Tyr-4<br><br>*Tyr-4*	124.3, C<br><br>*124.5, C*		Tyr-2, 3a, 3b, 6,6’<br>*Tyr-2, 3a, 3b, 6,6’*			
Tyr-5,5’<br><br>*Tyr-5,5’*	130.9, CH<br><br>*130.9, CH*	6.88, d (8.4)<br><br>*6.90, d (8.4)*	Tyr-3a, 3b, 5’,5, 6,6’<br>*Tyr-3a, 3b, 5’,5, 6,6’*	Tyr-6,6’<br><br>*Tyr-6,6’*		Tyr-2-NH, 3a, 3b, 6,6’
Tyr-6,6’<br>*Tyr-6,6’*	115.0, CH	6.61, d (8.4)<br>*6.62, d (8.4)*	Tyr-6’,6	Tyr-5,5’<br>*Tyr-5,5’*		Tyr-5,5’
Tyr-7<br>*Tyr-7*	156.7, C<br>*156.8, C*		Tyr-5,5’, 6,6’<br>*Tyr-5,5’, 6,6’*			
CO<br>*CO*	156.4, C<br>*156.6, C*		Tyr-2, 2-NH, Lys-2<br>*Tyr-2, 2-NH, Lys-2*			
Lys-1<br>*Lys-1*	167.9, C<br>*168.0, C*		Val-2-NH, Lys-2<br>*Val-2-NH, Lys-2*			
Lys-2<br>*Lys-2*	56.3, CH<br>*56.6, CH*	4.08, dd (12.0,5.0)<br>*4.09, dd (11.3,5.0)*		Lys-3a<br>*Lys-3a*	Lys-3a<br>*Lys-3a*	

Lys-3a<br>Lys-3b<br>*Lys-3a*<br>*Lys-3b*	29.3, CH_2	2.12, brdq (12.5,3.5)<br>0.84, m<br>*1.94, m*<br>*0.74, m*	Lys-2	Lys-2, 4b<br><br><br>*Lys-2,*		
Lys-4a<br>Lys-4b	22.9, CH_2	1.48, m<br>1.37, m		 Lys-3a		
Lys-5a<br>Lys-5b	28.6, CH_2	1.37, m<br>1.30, m		Lys-5b<br>Lys-5a		
Lys-6a<br>Lys-6b<br>*Lys-6a*<br>*Lys-6b*	38.4, CH_2<br><br>*38.2, CH_2*	3.54, m<br>2.63, m<br>*3.46, m*<br>*2.64, m*		Lys-6b, 6-NH<br>Lys-6a, 6-NH<br>*Lys-6b, 6-NH*<br>*Lys-6a, 6-NH*		Lys-6b<br>Lys-6a
Lys-6-NH		7.41, dd (7.2, 4.5)			Lys-6a, 6b	Phe-2-NH
Val-1<br>*Val-1*	172.4, C<br>*172.3, C*		Val-2, Hty-2-NH<br>*Val-2, Hty-2-NH*			
Val-2<br>*Val-2*	57.9, CH<br>*58.0, CH*	3.98, t (7.8)<br>*4.02, t (7.4)*	Val-4, 5<br>*Val-4, 5*	Val-2-NH, 3<br>*Val-2-NH, 3*	Val-2-NH, 3, 4, 5<br>*Val-2-NH, 3, 4, 5*	Val-2-NH<br>*Val-2-NH*
Val-2-NH<br>*Val-2-NH*		7.76, d (7.2)<br>*7.92, d (7.2)*		Val-2<br>*Val-2*	Val-2, 3, 4, 5<br>*Val-2, 3*	Val-2, 5<br>*Val-2-NH, Val-5*
Val-3<br>*Val-3*	30.4, CH	1.96, m<br>*1.98, m*	Val-2, 4, 5<br>*Val-2, 4, 5*	Val-2, 4, 5<br>*Val-2, 4, 5*		Val-2
Val-4<br>*Val-4*	19.1, CH_3	0.96, d (6.9)<br>*1.00, d (6.7)*	Val-2, 3, 5<br>*Val-2, 3, 5*	Val-3<br>*Val-3*		Val-2
Val-5<br>*Val-5*	18.9, CH_3	0.88, d (6.7)<br>*0.94, d (7.0)*	Val-2, 3, 4<br>*Val-2, 3, 4*	Val-3<br>*Val-3*		Val-2<br>*Phe-5,5'*
Hty-1<br>*Hty-1*	171.2, C<br>*171.0, C*		NMe-Ala-2, 3, Hty-2, 3a, *NMe-Ala-2, 3*			
Hty-2<br><br>*Hty-2*	48.9, CH<br><br>*49.0, CH*	4.74, m<br><br>*4.73, m*		Hty-2-NH, 3a, 3b	Hty-2-NH, 3a, 3b, 4a, 4b	

				Hty-2-NH, *3a,*<br>*3b*		
Hty-2-NH<br>*Hty-2-NH*		8.97, d (4.9)<br>*8.95, d (4.9)*			Hty-2	
Hty-3a<br>Hty-3b	33.5, CH_2	1.85, m<br>1.73, m		Hty-3b, 4a, 4b<br>Hty-3a, 4a, 4b		
Hty-4a<br>Hty-4b	30.7, CH_2	2.60, m<br>2.40, m	Hty-6,6'	Hty-3a, 3b, 4b<br>Hty-3a, 3b, 4a		
Hty-5	131.0, C		Hty-4b, 7,7'			
Hty-6,6'<br>*Hty-6,6'*	129.2, CH	6.98, d (8.5)<br>*6.97, d (8.6)*	Hty-4a, 4b, 6',6	Hty-7,7'<br>*Hty-7,7'*		Hty-7,7'
Hty-7,7'	115.3, CH	6.66, d (8.5)	Hty-7',7	Hty-6,6' *Hty-*<br>*7,7'*		Hty-6,6'
Hty-8	155.9, C		Hty-6,6', 7,7'			
NMe-Ala-1<br><br>*NMe-Ala-1*	170.0, C<br><br>*169.6, C*		Phe-2-NH, NMe-<br>Ala-2, 3<br>*Phe-2-NH, NMe-*<br>*Ala-2, 3*			
NMe-Ala-2<br>*NMe-Ala-2*	54.5, CH<br>*54.6, CH*	4.85, q (7.3)<br>*4.86, m*	NMe-Ala-3, NMe	NMe-Ala-3,		NMe-Ala-3,<br>NMe
NMe-Ala-3	14.1, CH_3	1.05, d (6.7)	NMe-Ala-2	NMe-Ala-2		NMe-Ala-2,<br>NMe
NMe-Ala-Me	27.2, CH_3	1.78, s	NMe-Ala-2			Phe-2, 2-NH,<br>NMe-Ala-2, 3
Phe-1	170.9, C		Lys-6-NH			
Phe-2<br><br>*Phe-2*	55.1, CH<br><br>*54.9, CH*	4.34, m<br><br>*4.33, m*	Phe-2-NH	Phe-3a, 3b, NH<br><br>*Phe-3a, 3b, NH*	Phe-3a, 3b, NH<br><br>*Phe-3a, 3b, NH*	Phe-2-NH, 3a,<br>5,5' NMe-Ala-<br>Me
Phe-2-NH<br><br>*Phe-2-NH*		8.69, d (8.9)<br><br>*8.66, d (8.6)*		4.34	Phe-2, 3a, 3b<br><br>*Phe-2*	Lys-6-NH, Phe-<br>3b, NMe-Ala-<br>Me
Phe-3a<br>Phe-3b	37.7, CH_2	3.23, dd (13.8, 3.0)<br>2.81, m	Phe-5,5'	Phe-2, 3b<br>Phe-2, 3a		Phe-2<br>Phe-2-NH, 5,5'

Phe-3a<br>*Phe-3b*	*37.6, CH_2*	*3.20, m*<br>*2.76, m*		*Phe-2*<br>*Phe-2*		
Phe-4	138.5, C		Phe-3b, 6,6’			
Phe-5,5’<br>*Phe-5,5’*	129.1, CH x2	7.06, d (7.2)<br>*7.05, d (7.2)*	Phe-3a, 3b, 5’,5			Phe-2, 3b<br>*Val-5*
Phe-6,6’	128.5, CH x2	7.19, t (7.2)	Phe-6’,6			
Phe-7	126.3, CH	7.14, t (7.2)	Phe-5,5’			

[a]500 MHz for ^{1}H and 125 MHz for ^{13}C, ca. 2:*1* major rotamer, *minor rotamer*. [b]From HSQC correlations. [c]J_{C-H}=8 Hz.

Figure S12. LCMS Chromatogram and Mass spectrum of Hydanto-anabaenopeptin A (**1**)

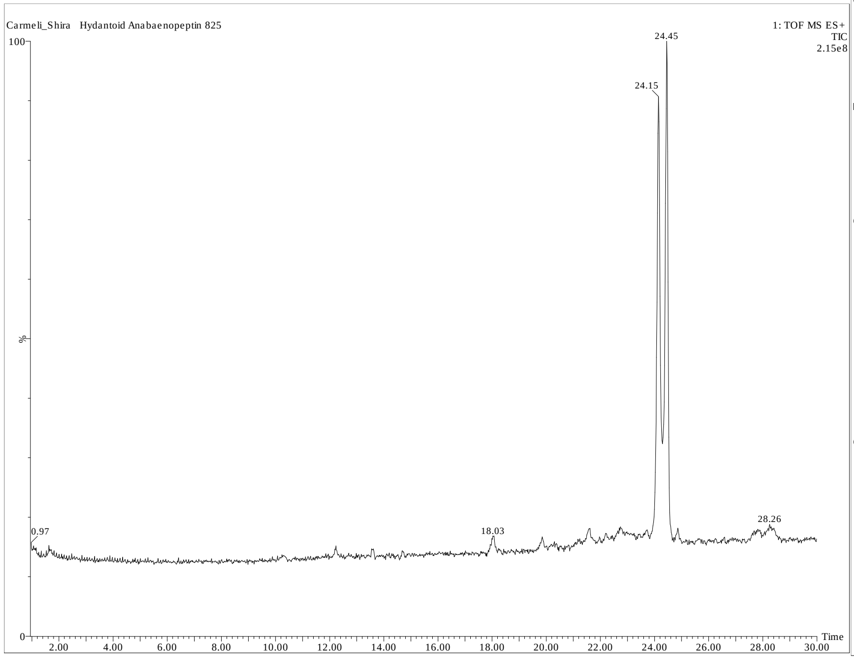

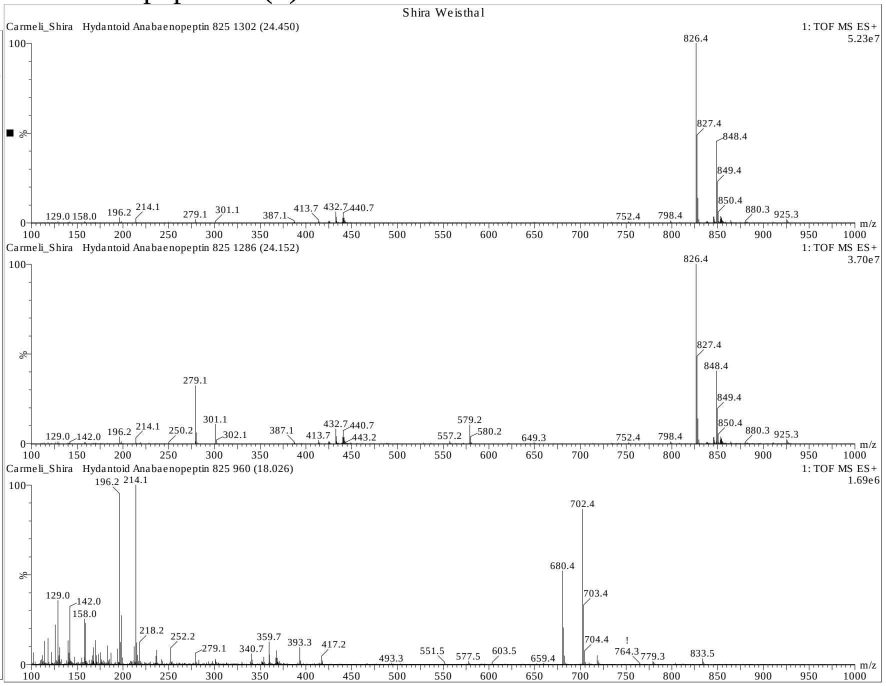

Figure S13. ^{1}H NMR Spectrum of Hydanto-anabaenopeptin B (**2**) in DMSO-d_6

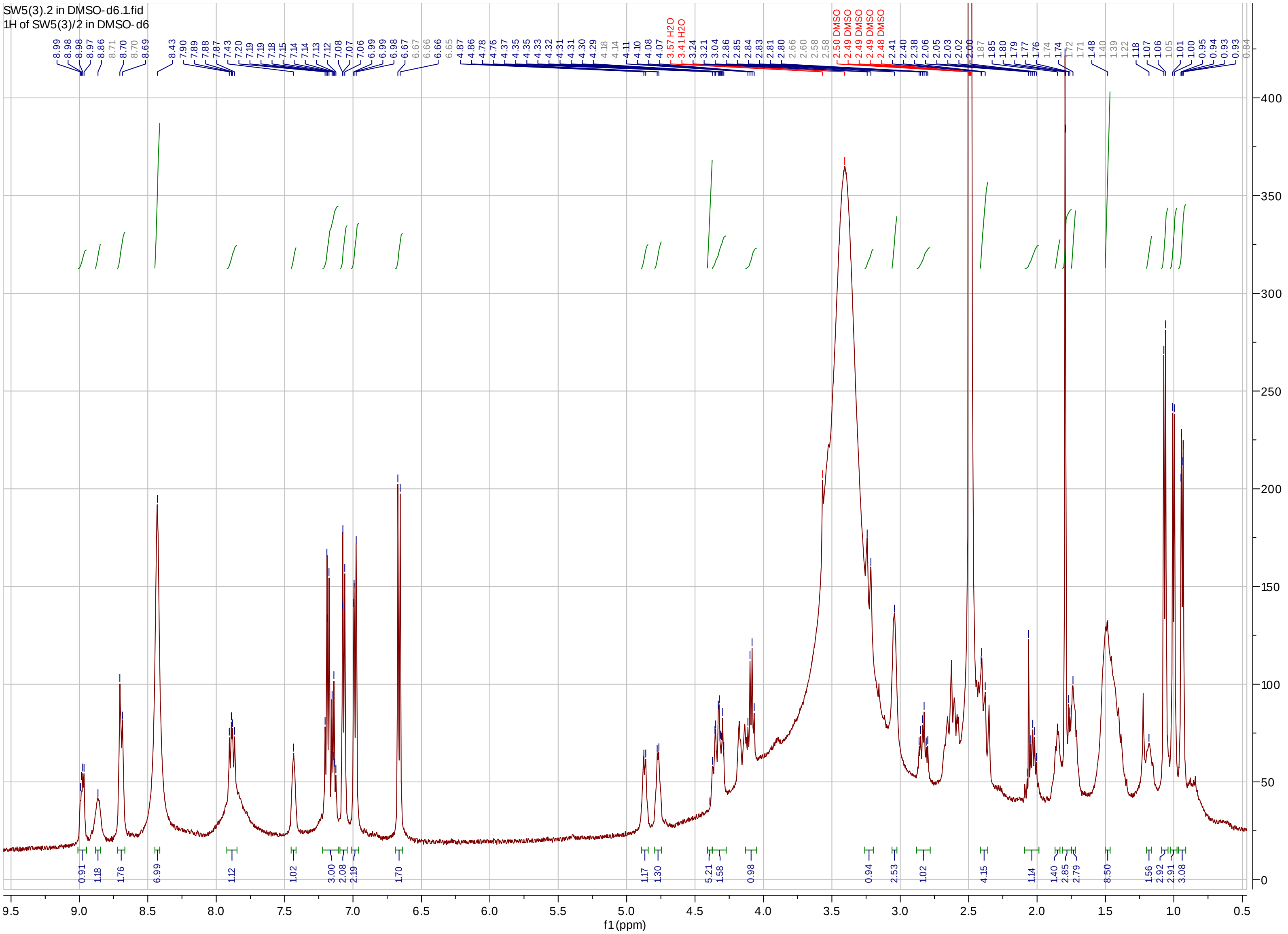

Figure S14. ^{13}C NMR Spectrum of Hydanto-anabaenopeptin B (**2**) in DMSO-d_6

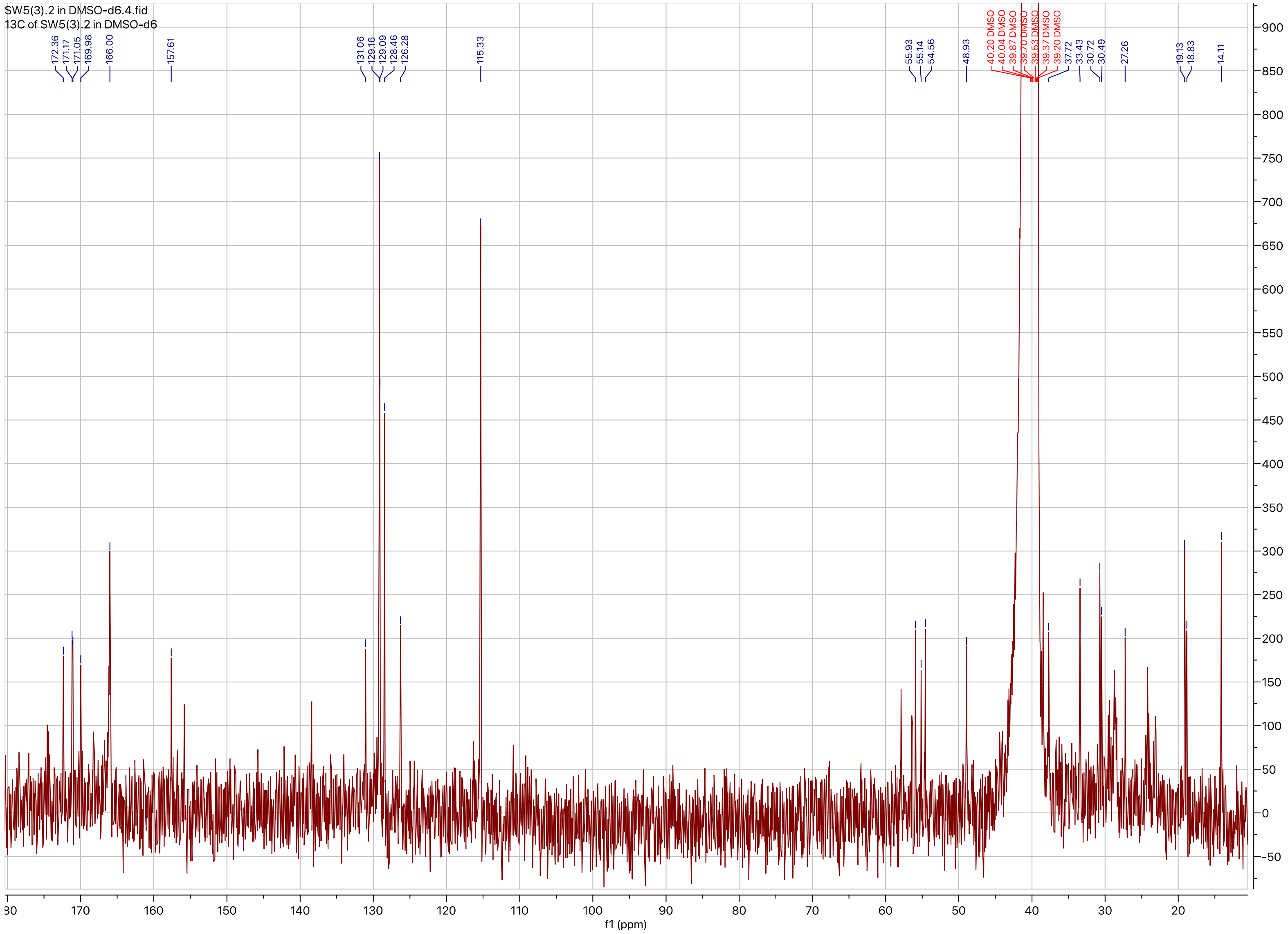

Figure S15. HSQC Spectrum of Hydanto-anabaenopeptin B (**2**) in DMSO-d_6

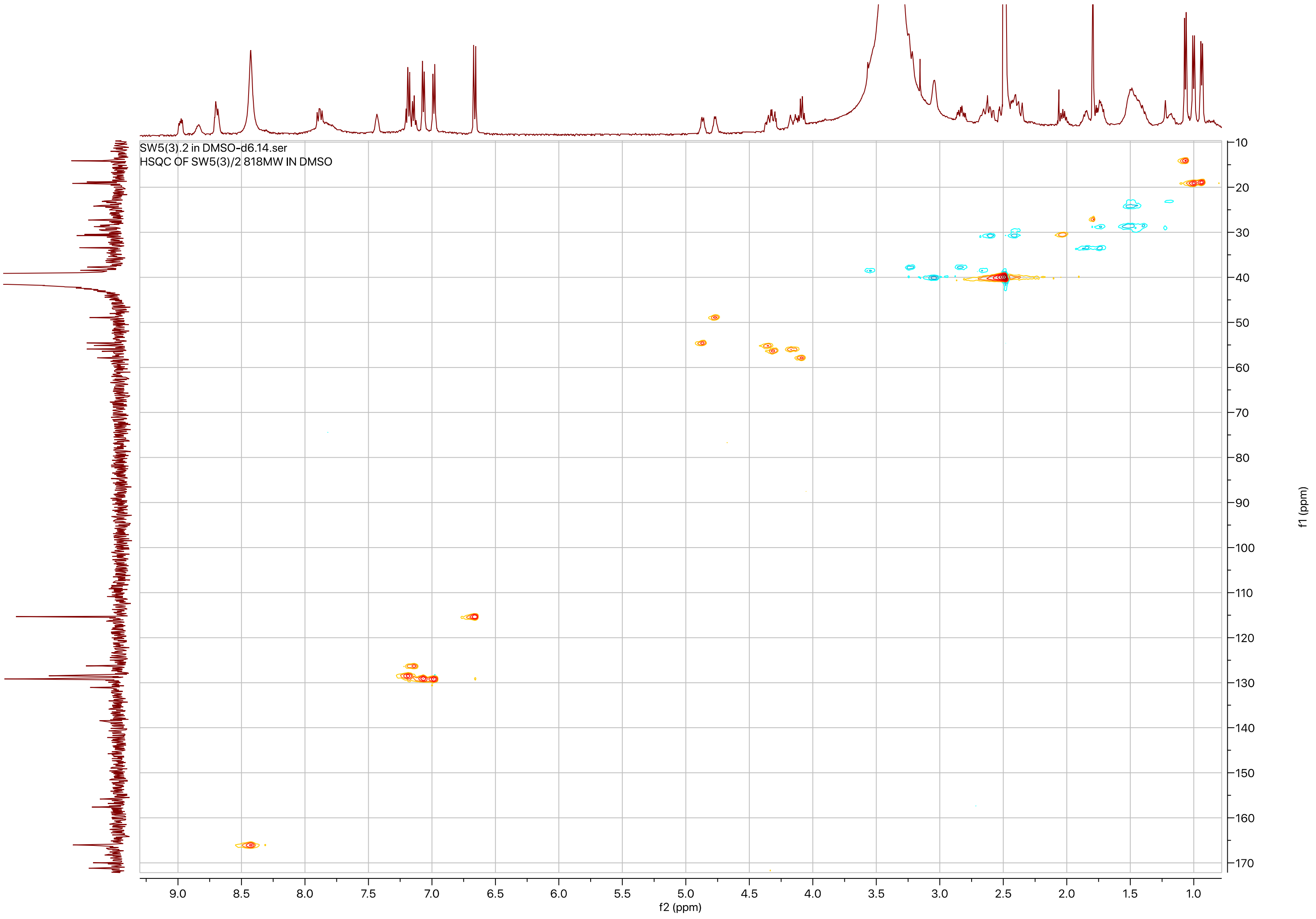

Figure S16. HMBC Spectrum of Hydanto-anabaenopeptin B (**2**) in DMSO-d_6

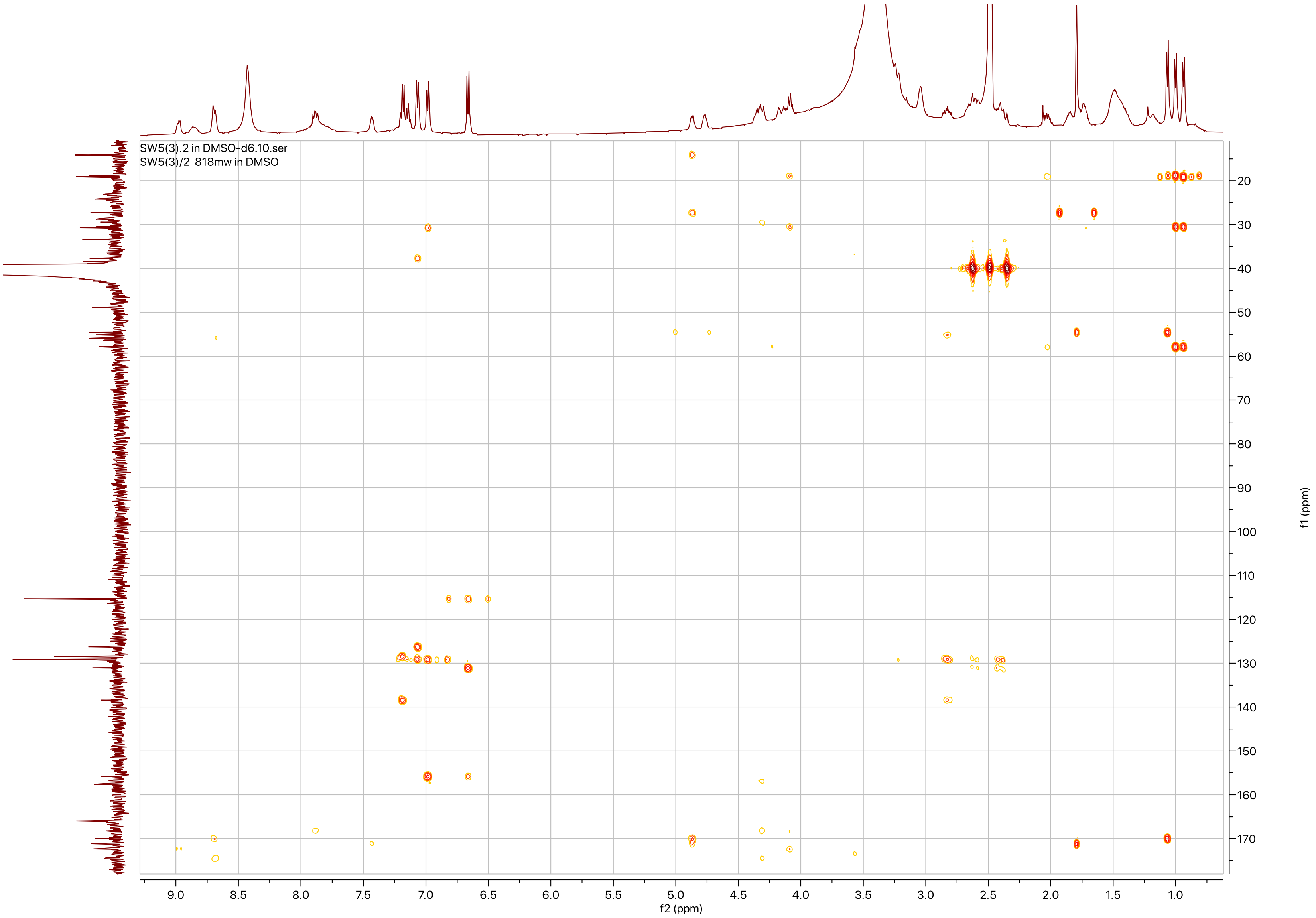

Figure S17. COSY Spectrum of Hydanto-anabaenopeptin B (**2**) in DMSO-d_6

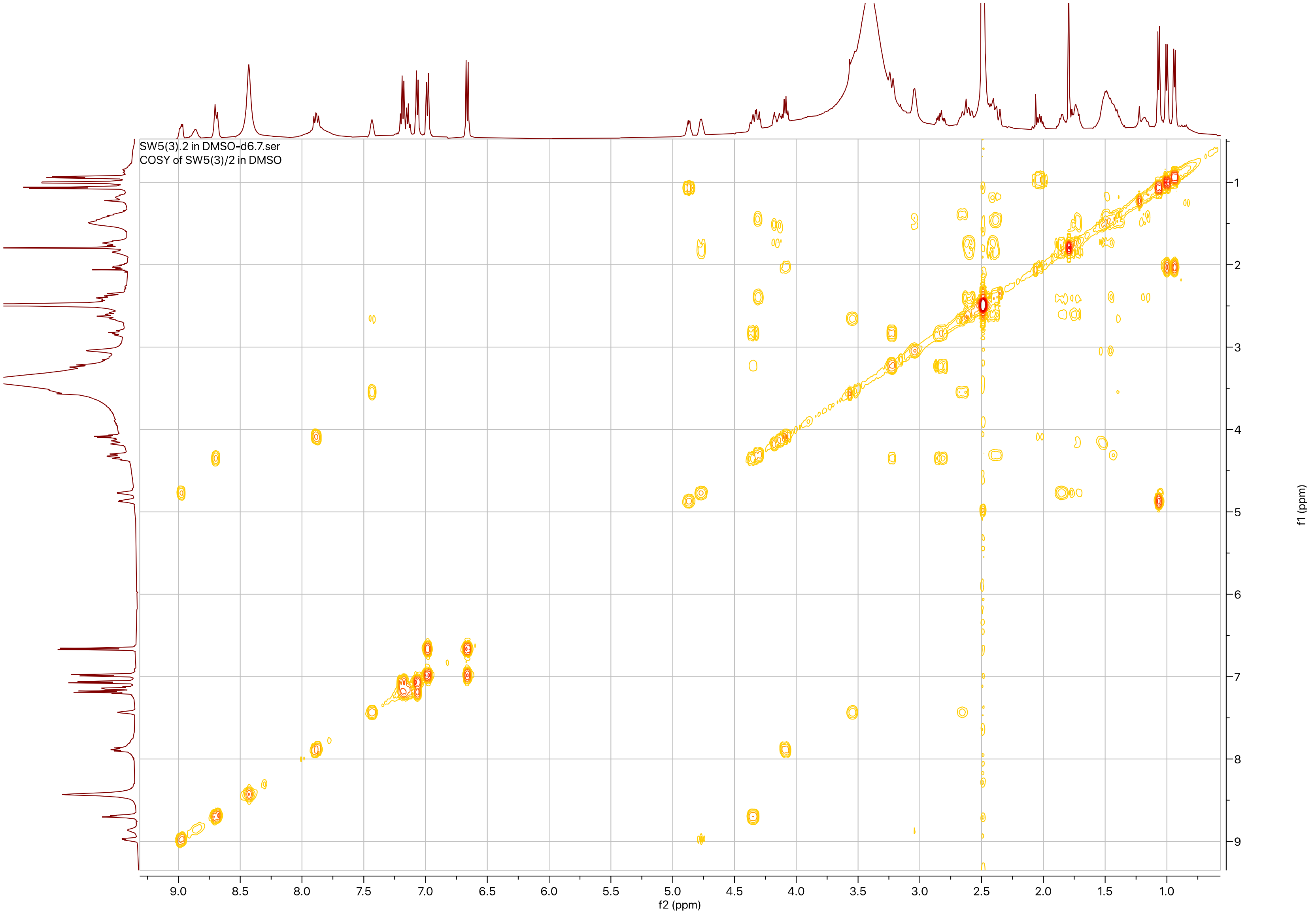

Figure S18. TOCSY Spectrum of Hydanto-anabaenopeptin B (**2**) in DMSO-d_6

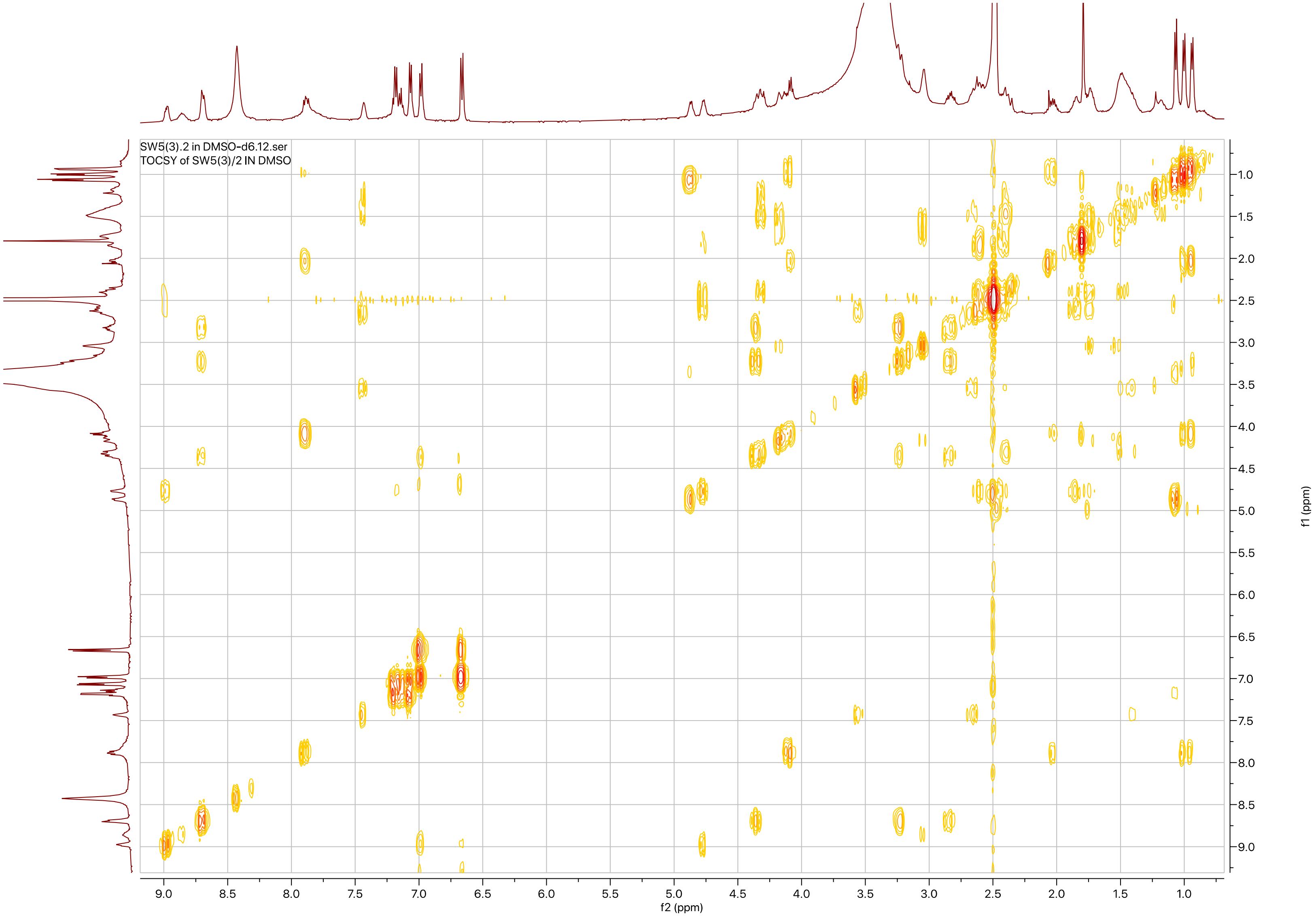

Figure S19. ROESY Spectrum of Hydanto-anabaenopeptin B (**2**) in DMSO-d_6

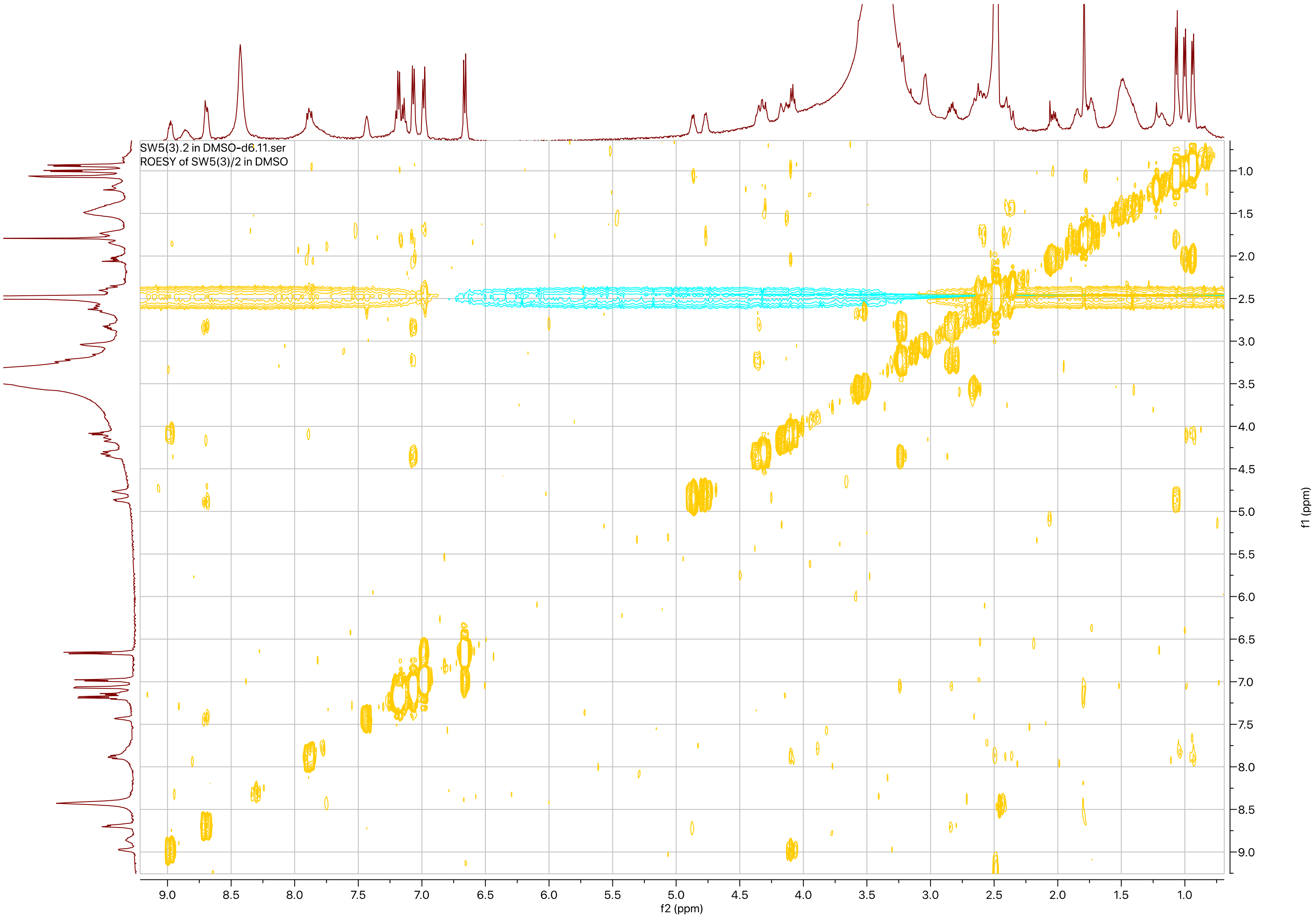

Figure S20. Negative HR ESI MS of Hydanto-anabaenopeptin B (**2**)

Single Mass Analysis
Tolerance = 3.0 PPM / DBE: min = -1.5, max = 50.0
Element prediction: Off
Number of isotope peaks used for i-FIT = 3
Monoisotopic Mass, Even Electron Ions
657 formula(e) evaluated with 3 results within limits (all results (up to 1000) for each mass)
Elements Used:

Mass	Calc. Mass	mDa	PPM	DBE	Formula	i-FIT	i-FIT Norm	Fit Conf %	C	H	N	O
817.4367	817.4361	0.6	0.7	18.5	C41 H57 N10 O8	543.1	1.694	18.38	41	57	10	8
	817.4374	-0.7	-0.9	23.5	C42 H53 N14 O4	545.6	4.141	1.59	42	53	14	4
	817.4347	2.0	2.4	13.5	C40 H61 N6 O12	541.6	0.223	80.03	40	61	6	12

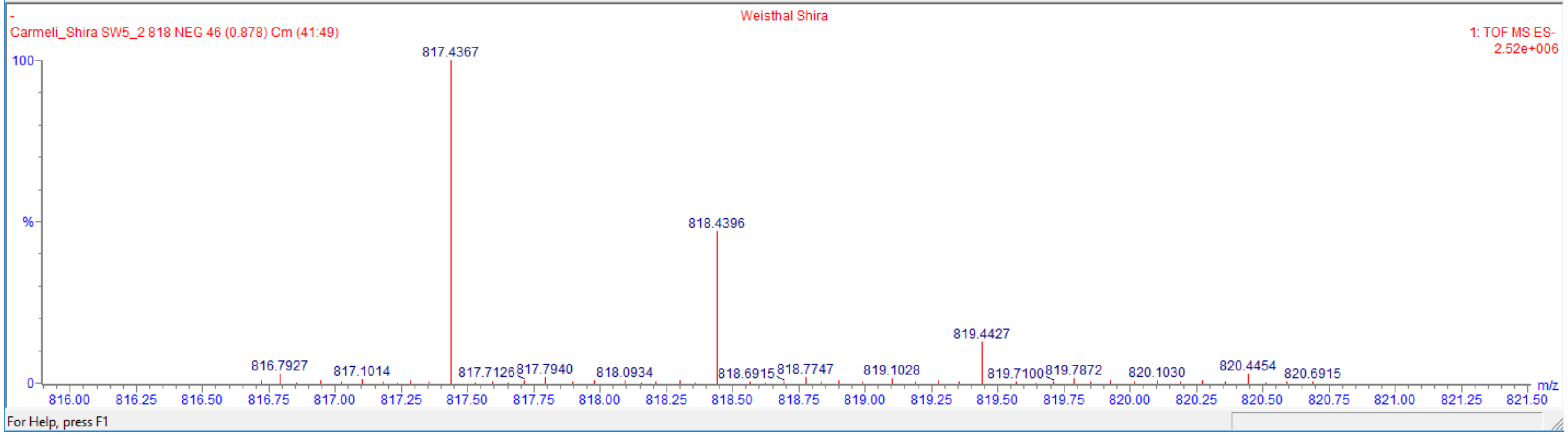

Figure S21. Positive HR ESI MS/MS Spectrum of Hydanto-anabaenopeptin B (**2**)

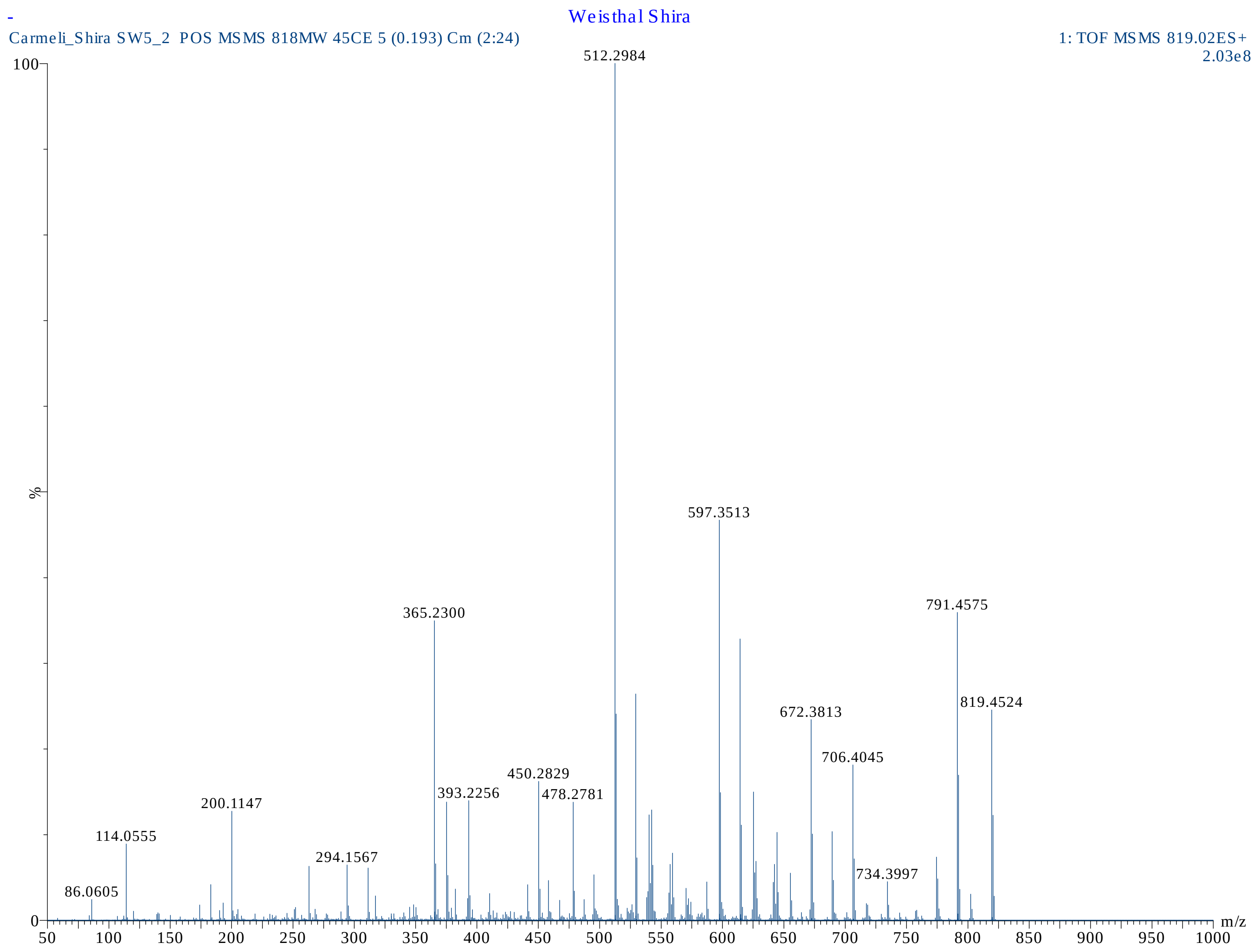

Figure S22. Scheme of Fragmentation in the Positive HR ESI MS/MS Spectrum of Hydanto-anabaenopeptin B (**2**)

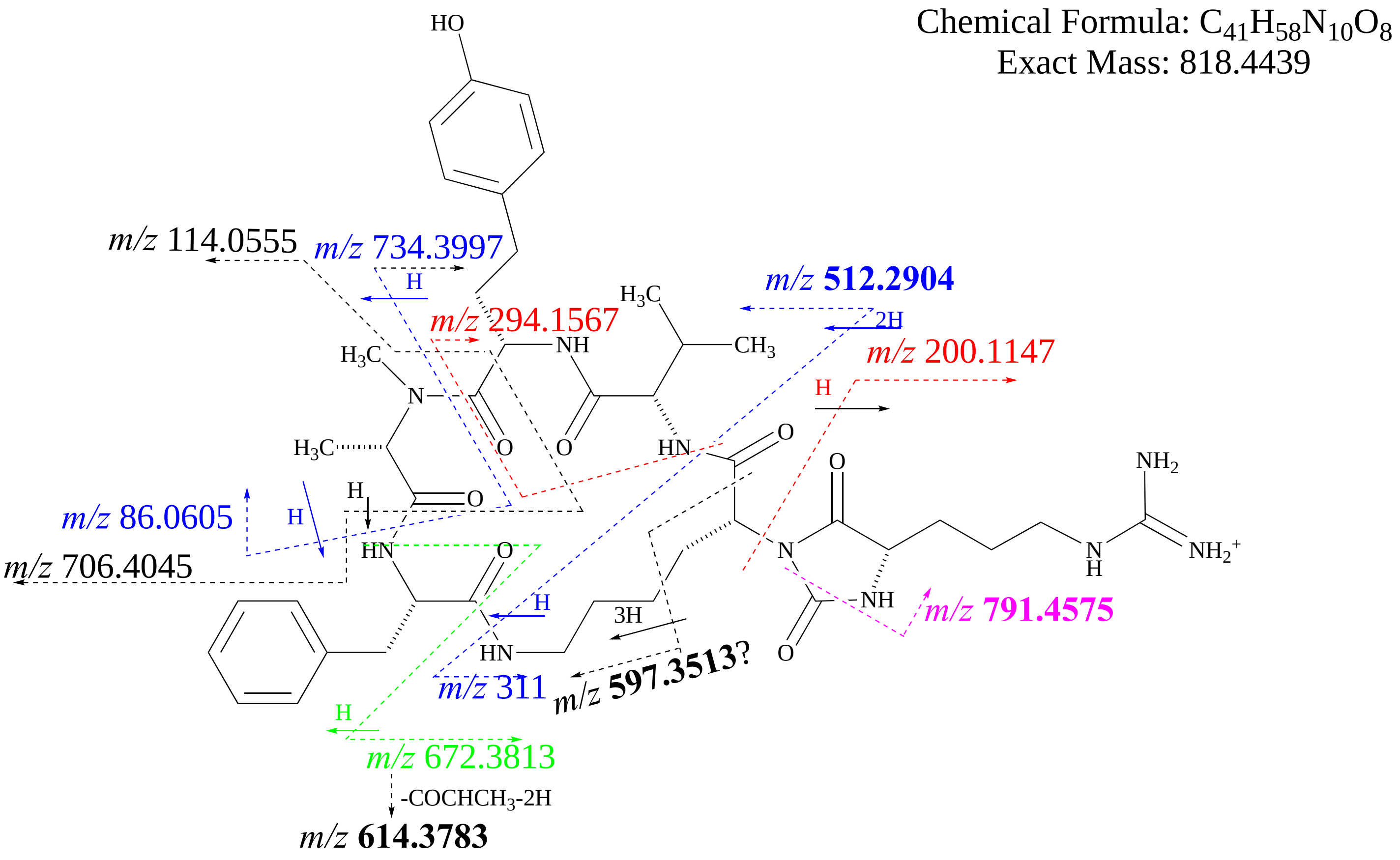

Figure S23. LCMS Traces of Marfey's Method Amino Acids Analysis of Hydanto-anabaenopeptin B (**2**)

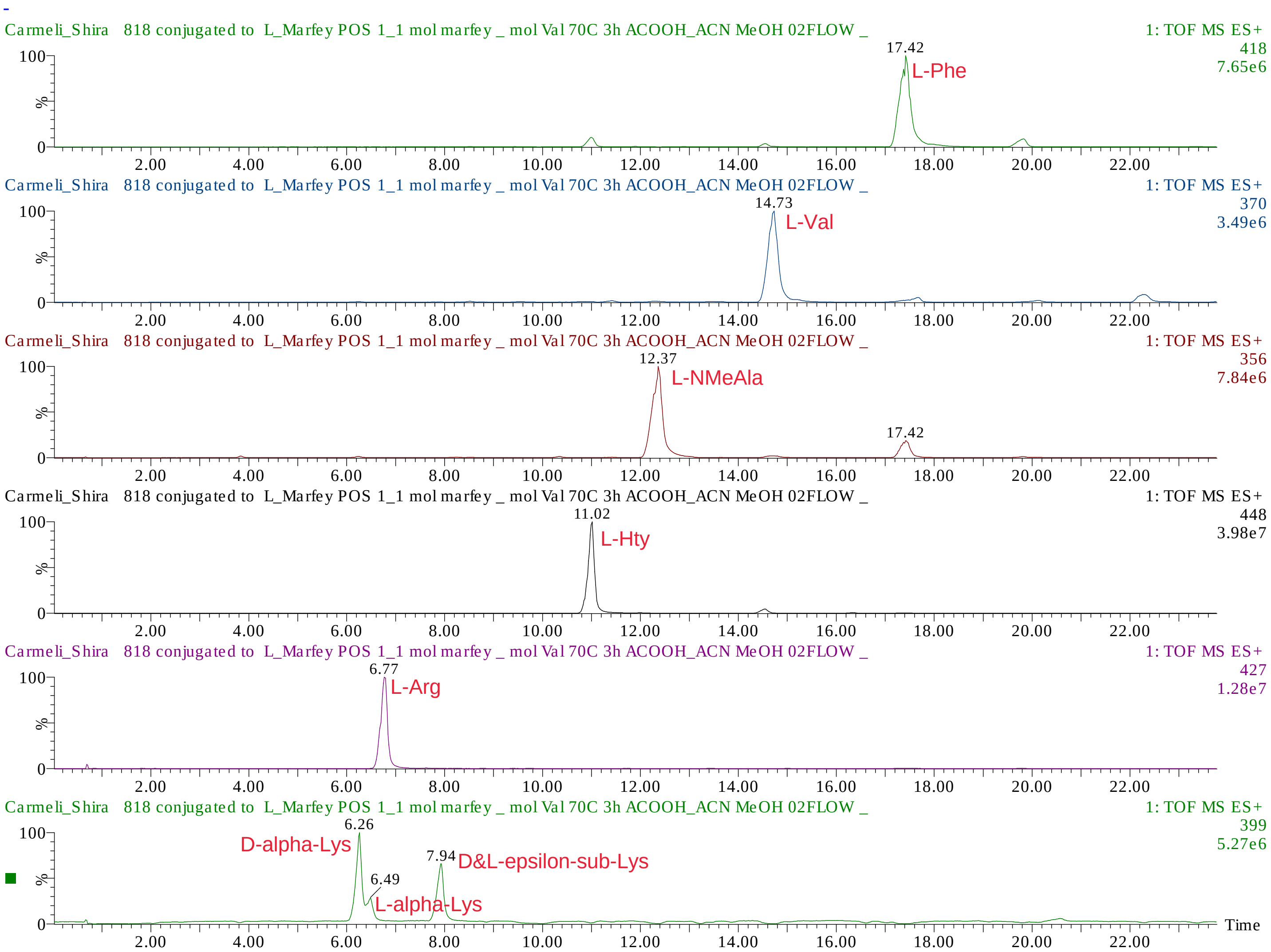

Table S2. NMR Data of Hydanto-anabaenopeptin B (**2**) in DMSO-d_6[a]

Position	δ_C, Mult.	δ_H, Mult. (J in Hz)[b]	HMBC correlations[c]	COSY correlations	TOCSY correlations	ROESY correlations
Arg-1<br><br>*Arg-1*	174.6, C<br><br>*174.3, C*		Arg-2, 2-NH, Lys-6-NH,<br>*Arg-2*			
Arg-2<br>*Arg-2*	55.9, CH<br>*55.7, CH*	4.17, t<br>*4.14, t*	Arg-2-NH<br>*Arg-2-NH*	Arg-2-NH, 3a, 3b<br>*Arg-2-NH, 3a, 3b*	Arg-3a, 3b, 5	Arg-2-NH
Arg-2-NH<br>*Arg-2-NH*		8.69, brs<br>*8.70, brs*		Arg-2		Arg-2
Arg-3a<br>Arg-3b<br>*Arg-3*	$28.8, CH_2$<br><br>*$28.6, CH_2$*	1.73, m<br>1.52, m		Arg-2, 3b, 4a, 4b<br>Arg-2, 3a, 4a, 4b	Arg-2, 3b, 4a, 4b<br>Arg-2, 3a, 4a, 4b	
Arg-4a<br>Arg-4b	$24.2, CH_2$	1.54, m<br>1.45, m		Arg-5		
Arg-5	$40.1, CH_2$	3.04, brm		Arg-4a, 4b, 5-NH	Arg-3a, 3b, 4a, 4b, 5-NH	
Arg-6-NH		8.86, brs		Arg-5	Arg-5, 4a, 4b	
Arg-7	156.7, C		Arg-4a, 4b, 6-NH			
Arg-7-NH_2, NH_2^+		8.43, brs				
CO<br><br>*CO*	156.9, C<br><br>*156.8, C*		Arg-2-NH, Lys-6-NH			
Lys-1	168.2, C		Val-2, NH Lys-2			
Lys-2<br><br>*Lys-2*	56.4, CH<br><br>*56.3, CH*	4.30, t (4.1)<br><br>*4.32, t (4.1)*		Lys-3a, 3b	Lys-3a, 3b, 4a, 4b, 5a, 5b	
Lys-3a<br>Lys-3b<br>*Lys-3*	$29.6, CH_2$<br><br>*$29.4, CH_2$*	2.40, m<br>1.45, m	Lys-2	Lys-2, 3b, 4b<br>Lys-2, 3a, 4b	Lys-2, 3b, 4a, 4b<br>Lys-2, 3a, 4a, 4b	Val-NH
Lys-4a	$23.1, CH_2$	1.51, m		Lys-3a, 3b, 4b<br>Lys-3a, 3b, 4a	Lys-6-NH	

Lys-4b		1.18, m			Lys-6-NH	
Lys-5a<br>Lys-5b<br>*Lys-5*	28.5, CH_2<br><br>*28.4, CH_2*	1.50, m<br>1.40, m		Lys-4b, 6a, 6b,<br>Lys-5b, 6b	Lys-6-NH<br>Lys-6-NH	
Lys-6a<br><br>Lys-6b	38.5, CH_2	3.55, m<br><br>2.66, m	Lys-4a	Lys-5a, 5b, 6b, 6-NH<br>Lys-5b, 6a, 6-NH	Lys-5a, 5b, 6b, 6-NH<br>Lys-5a, 5b, 6a, 6-NH	Lys-6b<br><br>Lys-6a
Lys-6-NH		7.43, m		Lys-6a, 6b	Lys-4a, 4b, 5a, 5b, 6a, 6b	Phe-NH
Val-1	172.4, C		Hty-NH, Val-2, 3			
Val-2	57.9, CH	4.09, q (7.5)	Val-3, 4, 5	Val-3, NH	Val-3, 4, 5, NH	Hty-NH, Val-NH
Val-NH<br>*Val-NH*		7.87, d (7.5)<br>*7.89, d (7.5)*		Val-2	Val-2, 3, 4, 5	Val-2, 5, Lys-3a
Val-3	30.5, CH	2.03, dqq (7.5, 6.6, 6.5)	Val-4, 5	Val-2, 4, 5	Val-NH	Val-4, 5, Phe-5,5'
Val-4	19.1, CH_3	1.00, d (6.6)	Val-2, 3, 5	Val-3	Val-3	
Val-5<br>*Val-5*	18.9, CH_3<br>*18.8, CH_3*	0.94, d (6.5)<br>*0.94, d (6.5)*	Val-2, 3, 4	Val-3		Val-3
Hty-1	171.1, C		NMeAla-2, NMe, Hty-2,3a, 3b			
Hty-2	48.9, CH	4.77, brdt (4.7, 6.5)	Hty-3b	Hty-3a, 3b, NH	Hty-3a, 3b, 4a, 4b, NH	
Hty-NH		8.98, d (4.7)<br>*8.98, d (4.7)*		Hty-2	Hty-2, 4a	Val-2
Hty-3a<br>Hty-3b	33.4, CH_2	1.85, m<br>1.74, m	Hty-2, 2-NH, 4b	Hty-2, 3b, 4a, 4b<br>Hty-2, 3a, 4a, 4b	Hty-2, 3b, 4a, 4b<br>Hty-2, 3a, 4a, 4b	
Hty-4a<br>Hty-4b	30.7, CH_2	2.61, m<br>2.42, m	Hty-6,6'	Hty-3a, 3b, 4b<br>Hty-3a, 3b, 4a	Hty-2, 3a, 3b, 4b<br>Hty-2, 3a, 3b, 4a	Hty-6,6'
Hty-5	131.1, C		Hty-4a,4b,7,7'			
Hty-6,6'	129.2, CH x2	6.98, d (8.5)	Hty-4a, 4b, 6',6, 7,7'			Hty-3a, 7,7'

Hty-7,7’	115.3, CH x2	6.66, d (8.5)	Hty-6,6’, 7’,7			Hty-6,6’
Hty-8	155.8, C		Hty-6,6’, 7,7’			
NMeAla-1	170.0, C		NMeAla-2, 2-NH, 3			
NMeAla-2	54.6, CH	4.87, dq (2.7, 6.7)	NMeAla-3, NMe	NMeAla-3		NMeAla-3
NMeAla-NMe	27.3, CH_3	1.79, s	NMeAla-2			NMeAla-3
NMeAla-3	14.1, CH_3	1.07, d (6.7)	NMeAla-2	NMeAla-2	NMeAla-2	NMeAla-2, NMe
Phe-1	171.2, C		Lys-6-NH, Phe-3b			
Phe-2	55.1, CH	4.36, m	Phe-3b	Phe-3a, 3b, NH	Phe-3a, 3b, NH	Phe-3a, 3b, 5,5’
Phe-NH		8.70, (9.0)<br>*8.69, (8.0)*		Phe-2	Phe-2, 3a, 3b	Lys-6-NH, Phe-3b
Phe-3a<br><br>Phe-3b	37.7, CH_2	3.23, brd (13.2)<br>2.83, dd (13.2, 5.6)	Phe-5,5’	Phe-2, 3b<br><br>Phe-2, 3a	Phe-NH<br><br>Phe-NH	Phe-2, 3b, 5,5’<br>Phe-3a, 5,5’
Phe-4	138.4, C		Phe-3a, 3b, 6,6’			
Phe-5,5’	129.0, CH x2	7.07, d (7.1)	Phe-3a, 3b, 5’,5, 7	Phe-6,6’		NMeAla-NMe, Val-3, 5, Phe-2, 3a, 3b,
Phe-6,6’	128.5, CH x2	7.19, t (7.1)	Phe-6’,6	Phe-5,5’, 7		NMeAla-NMe
Phe-7	126.3, CH	7.14, t (7.1)	Phe-5,5’	Phe-6,6’		

[a]500 MHz for ^{1}H and 125 MHz for ^{13}C, ca. 1:*1* major rotamer, *minor rotamer*. [b]From HSQC correlations. [c]J_{C-H}=8 Hz.

Figure S24. LCMS Chromatogram and Mass spectrum of Hydanto-anabaenopeptin B (**2**)

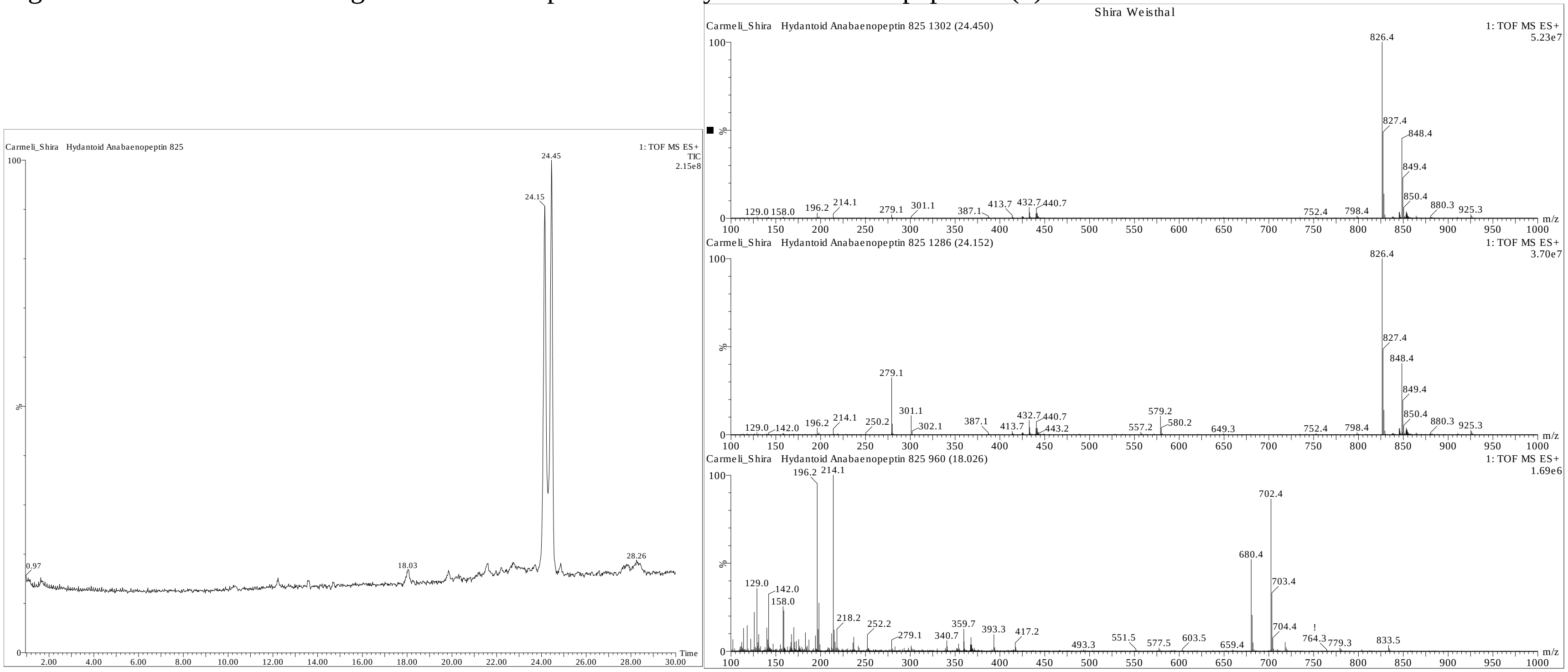

Figure S25. ^{1}H NMR Spectrum of Hydanto-anabaenopeptin F (**3**) in DMSO-d_6

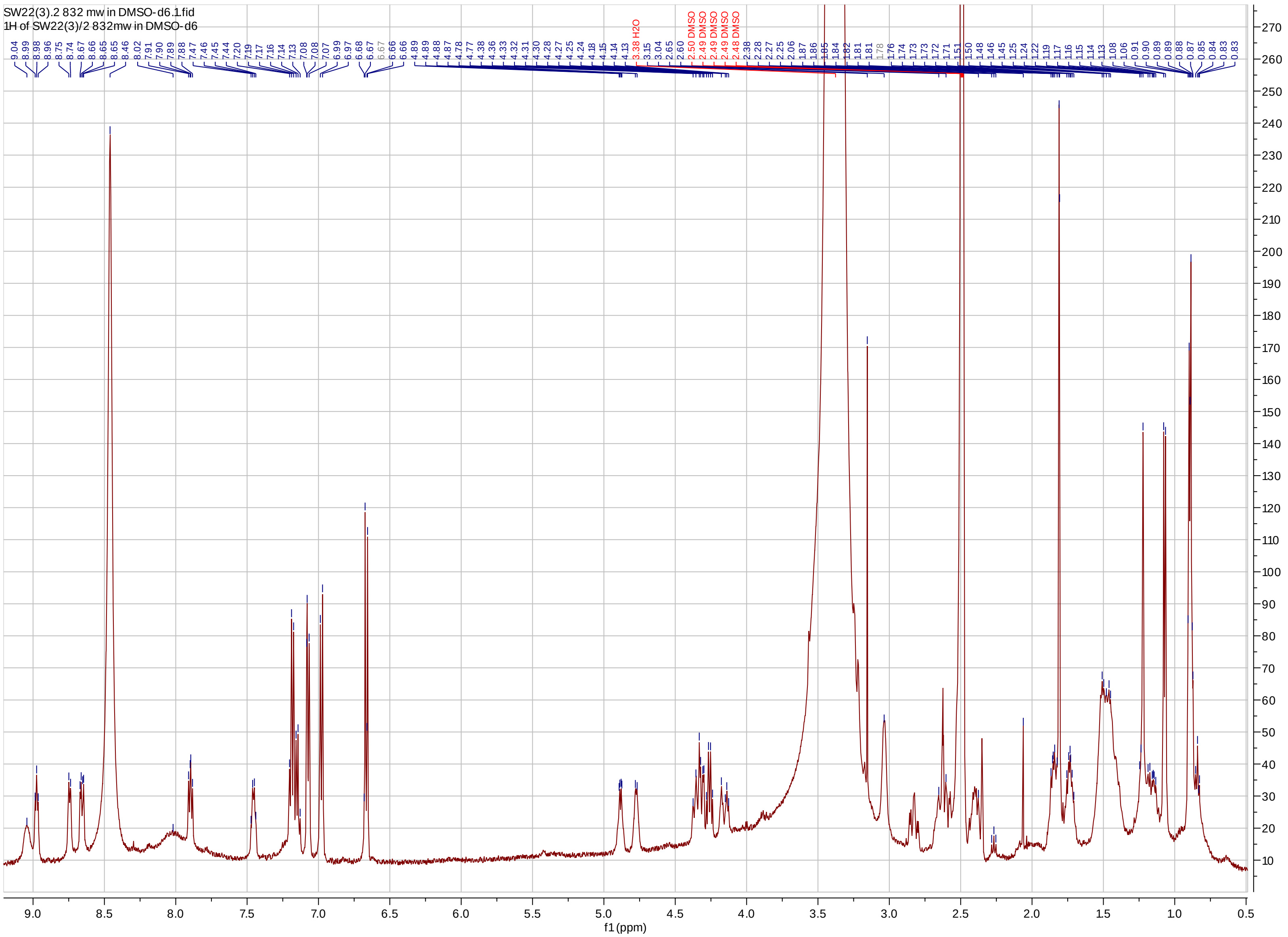

Figure S26. ^{13}C NMR Spectrum of Hydanto-anabaenopeptin F (**3**) in DMSO-d_6

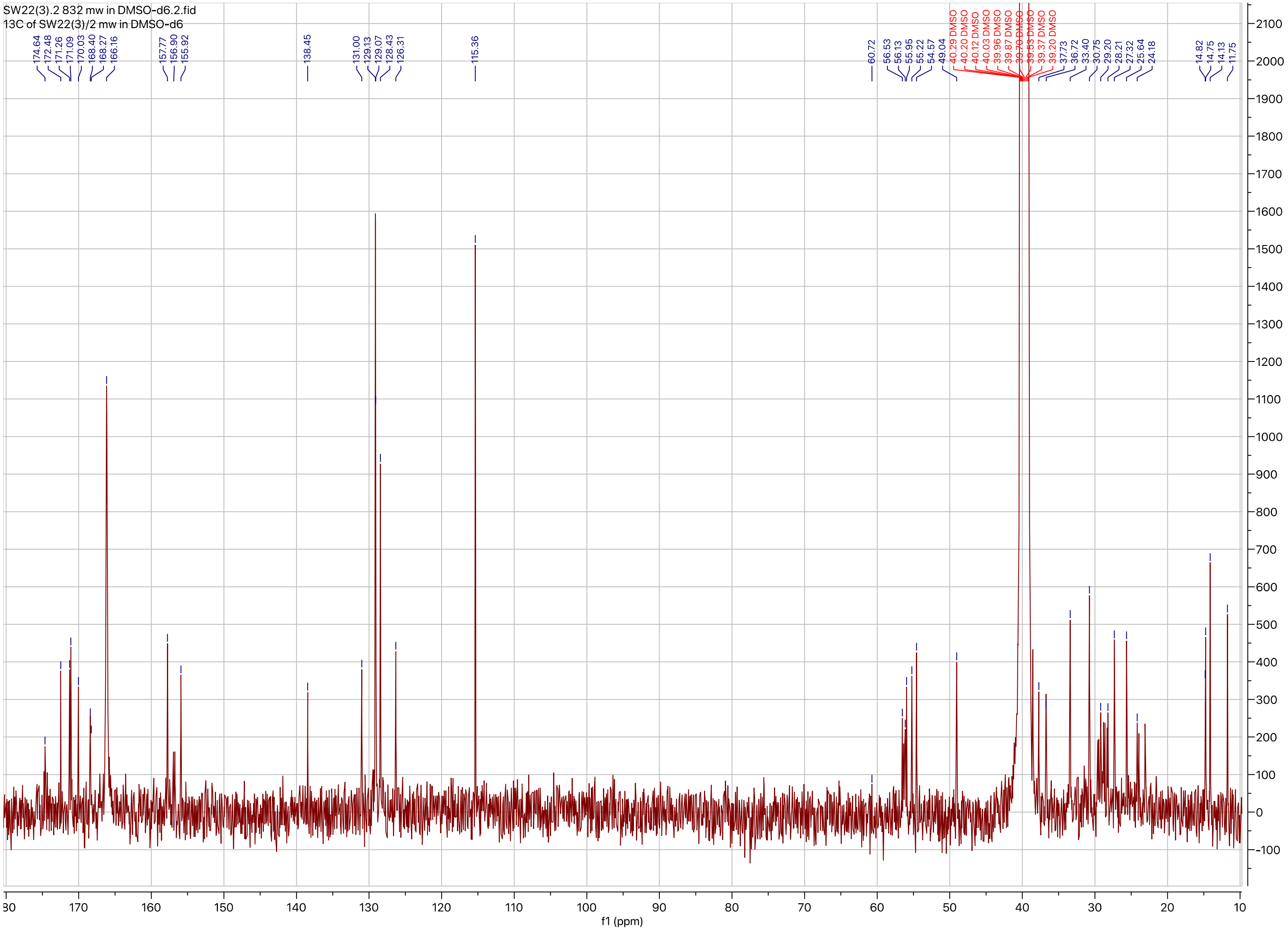

Figure S27. HSQC Spectrum of Hydanto-anabaenopeptin F (**3**) in DMSO-d_6

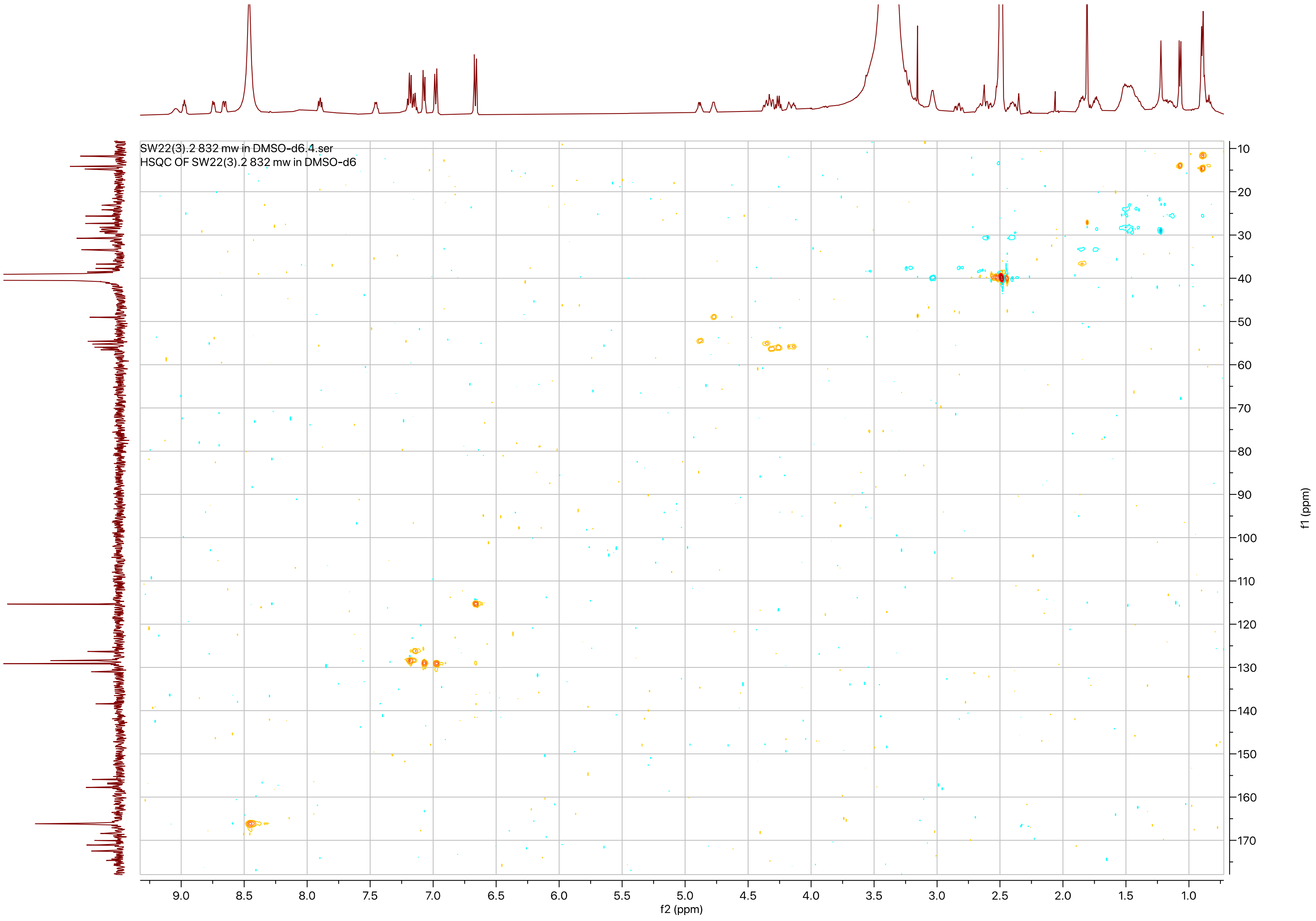

Figure S28. HMBC Spectrum of Hydanto-anabaenopeptin F (**3**) in DMSO-d_6

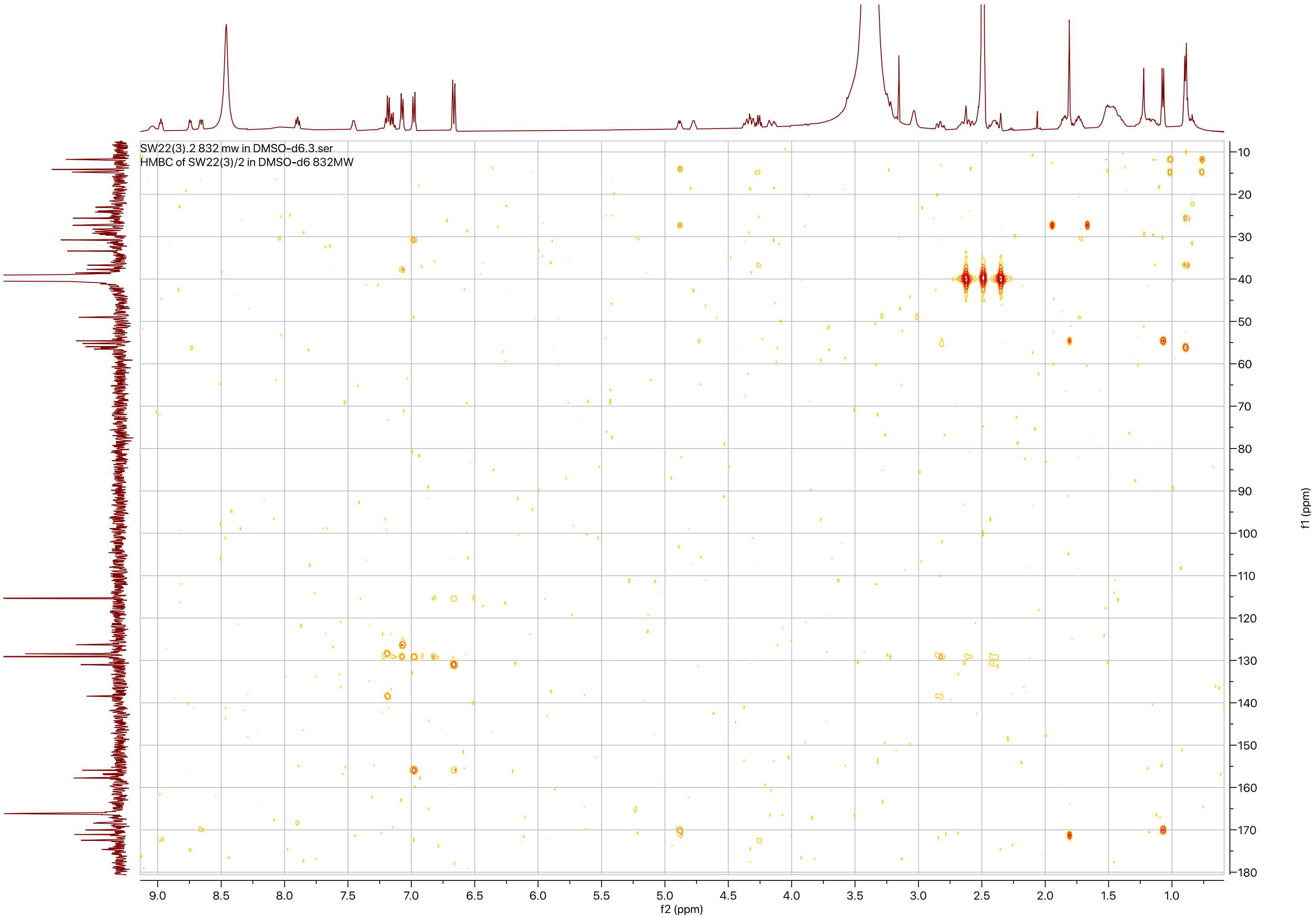

Figure S29. COSY Spectrum of Hydanto-anabaenopeptin F (**3**) in DMSO-d_6

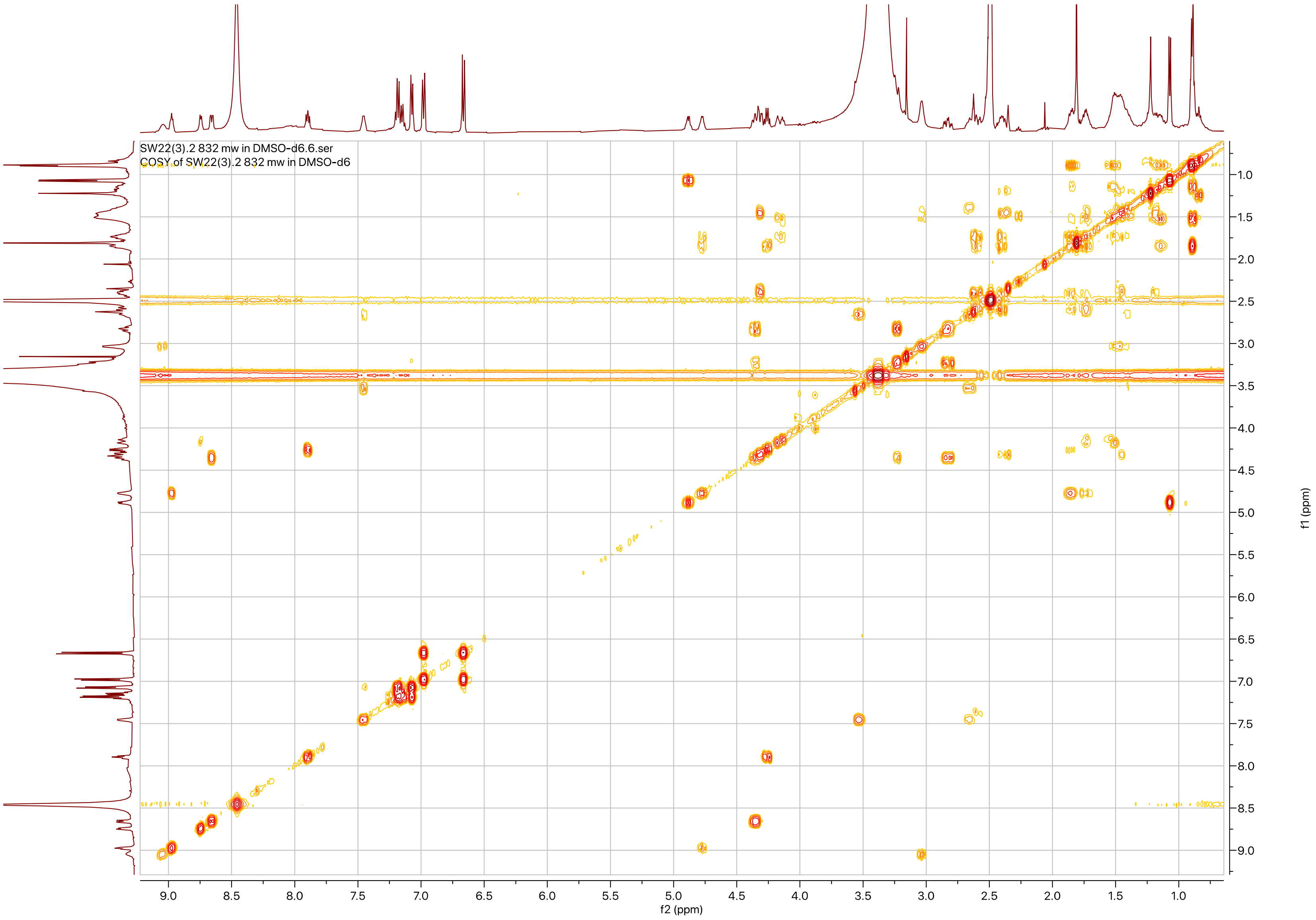

Figure S30. TOCSY Spectrum of Hydanto-anabaenopeptin F (**3**) in DMSO-d_6

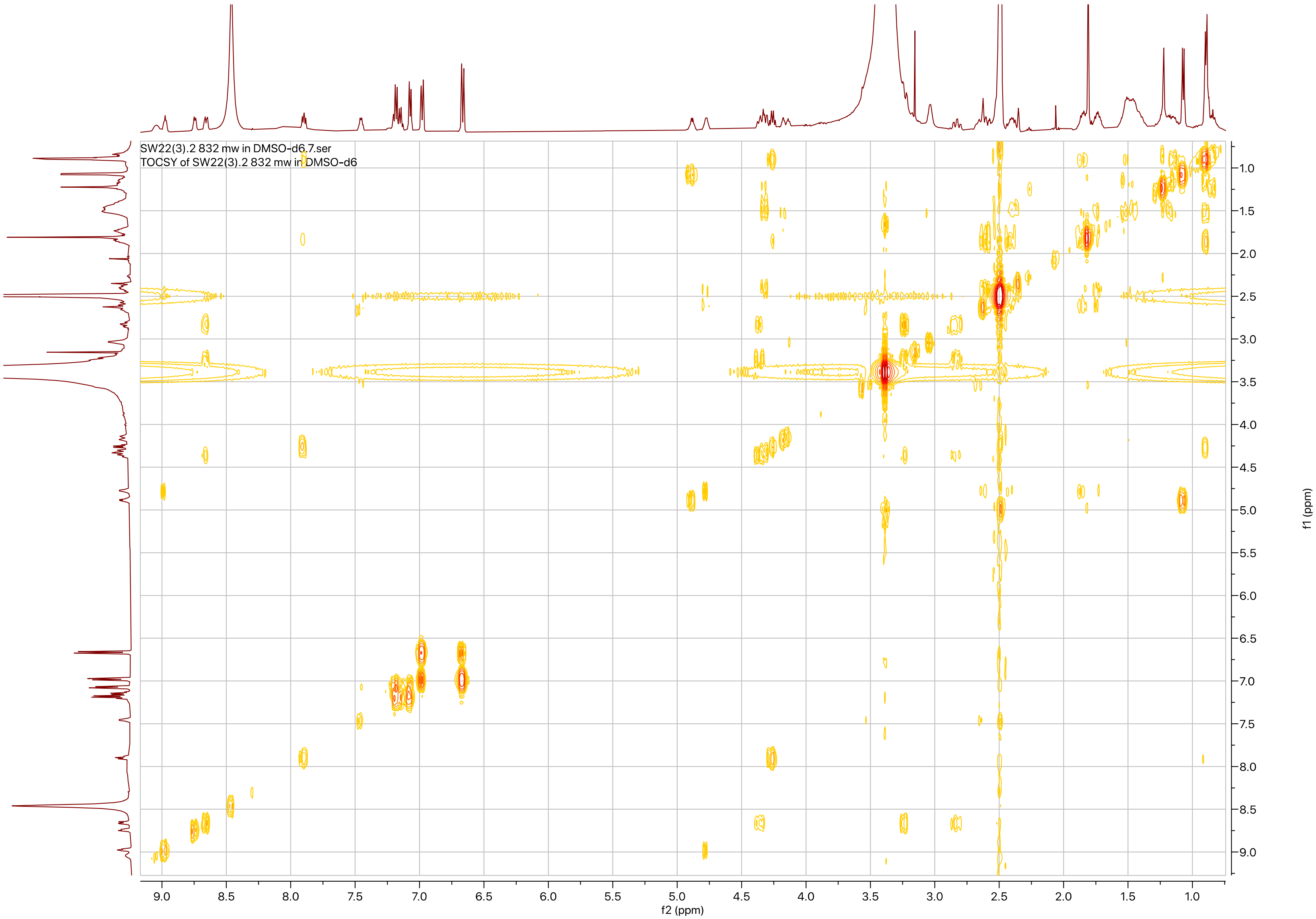

Figure S31. ROESY Spectrum of Hydanto-anabaenopeptin F (**3**) in DMSO-d_6

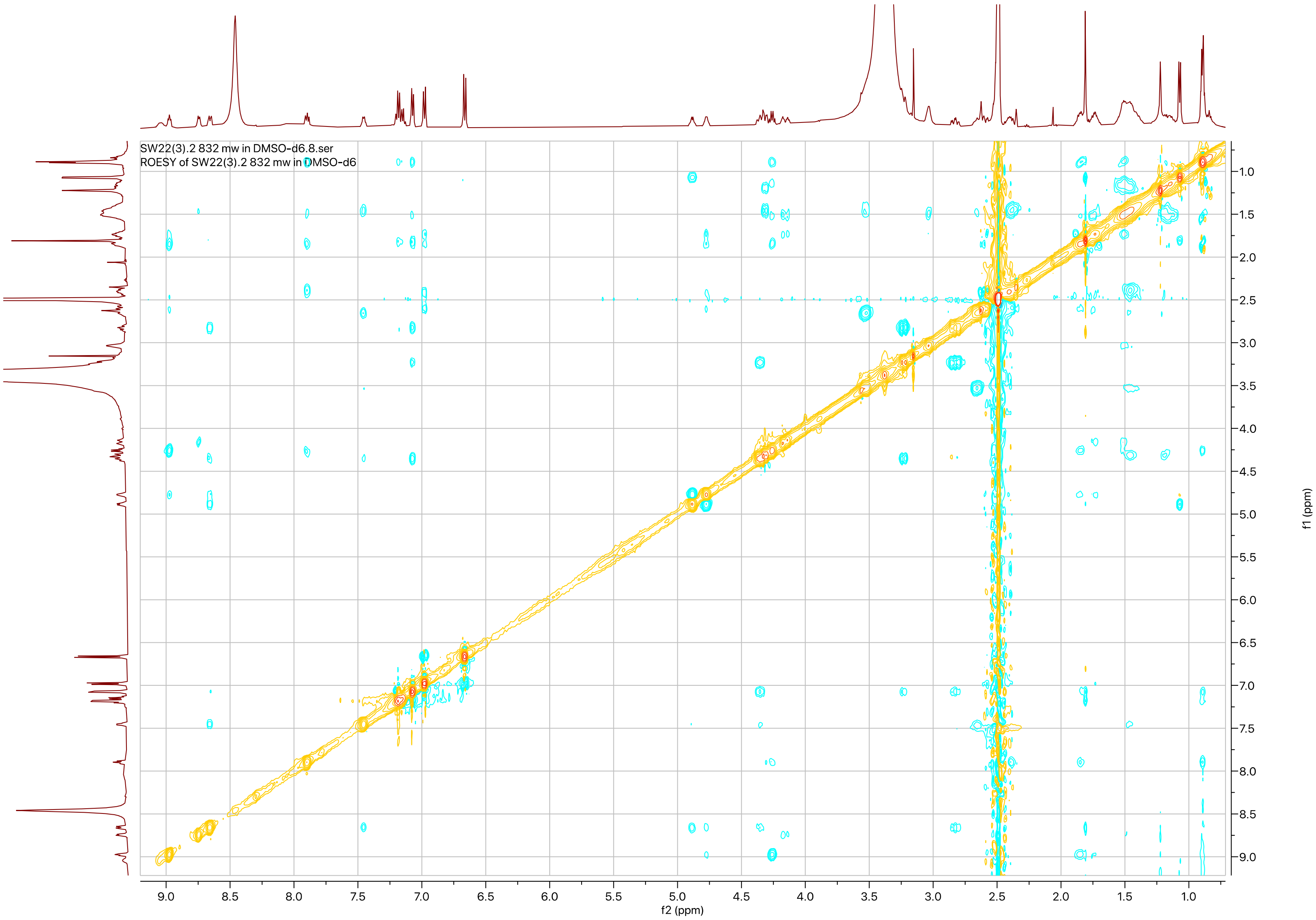

Figure S32. Negative HR ESI MS of Hydanto-anabaenopeptin F (**3**)

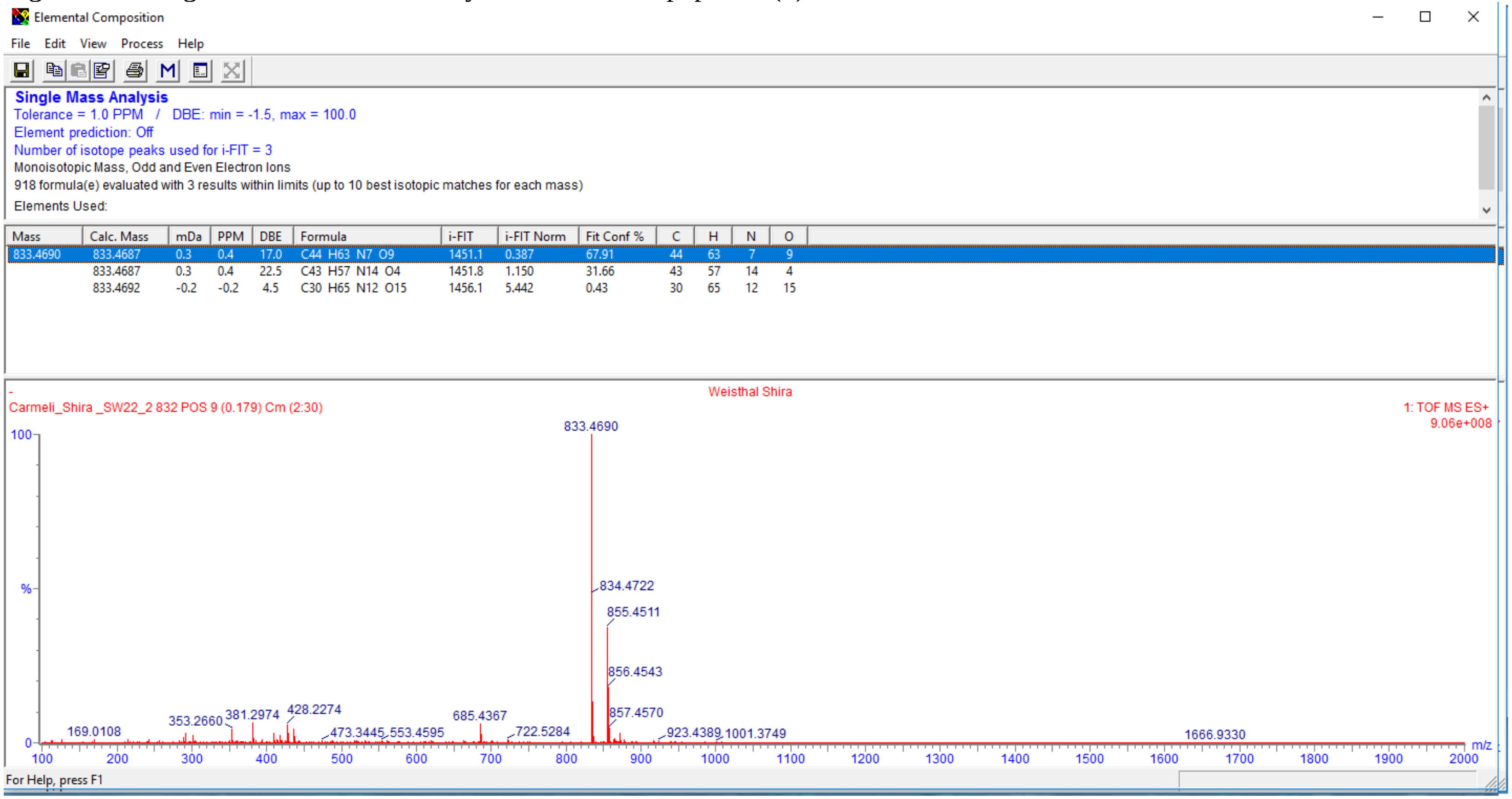

Figure S33. Positive HR ESI MS/MS Spectrum of Hydanto-anabaenopeptin F (**3**)

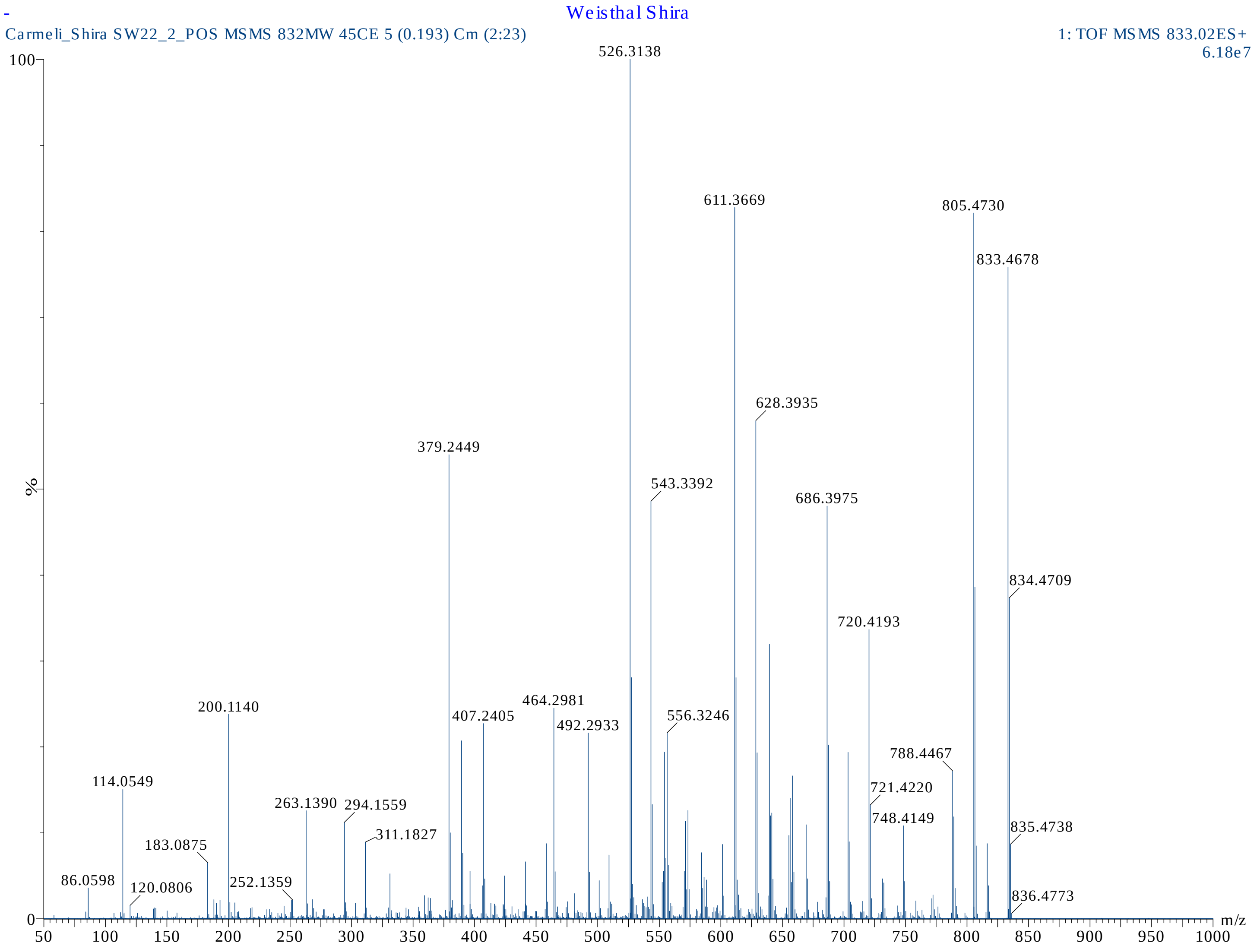

Figure S34. Scheme of Fragmentation in the Positive HR ESI MS/MS Spectrum of Hydanto-anabaenopeptin F (**3**)

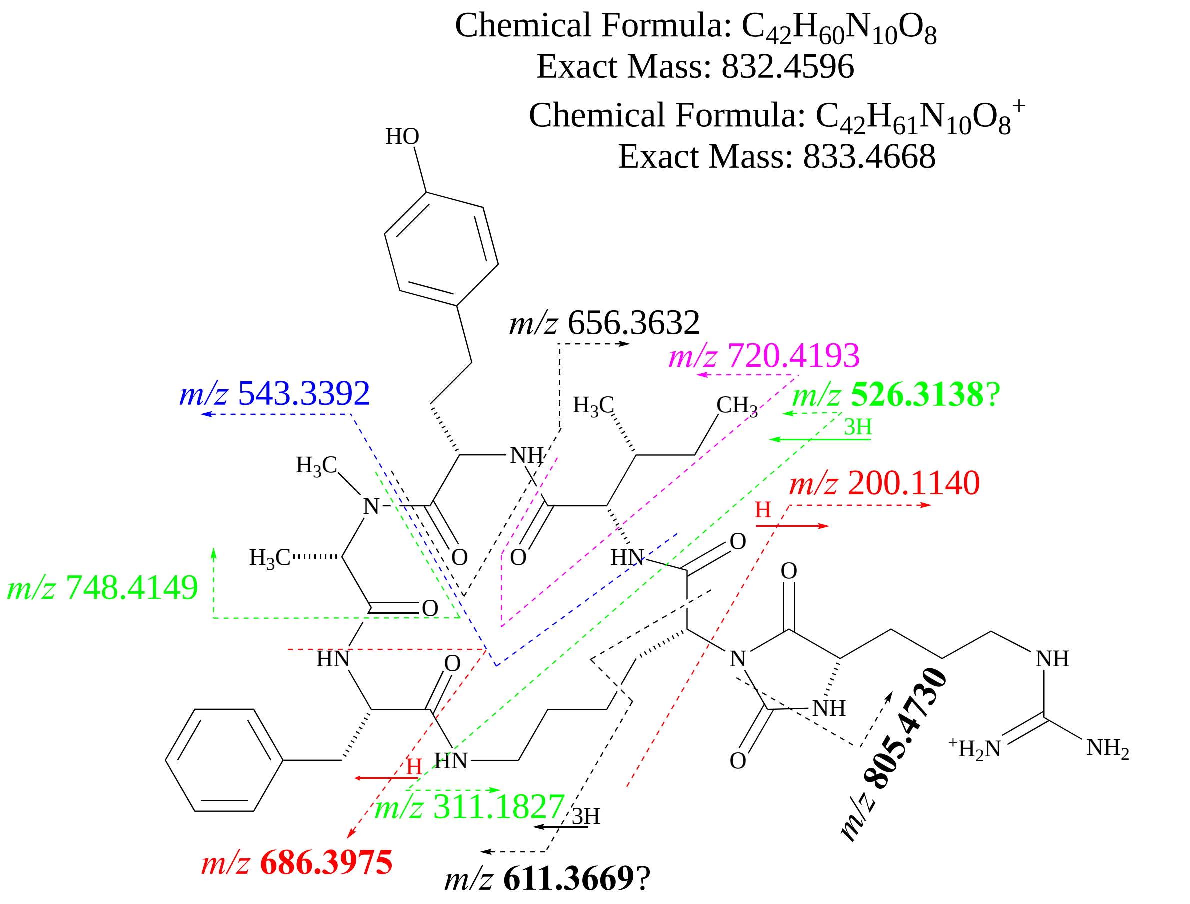

Figure S35. LCMS Traces of Marfey's Method Amino Acids Analysis of Hydanto-anabaenopeptin F (**3**)

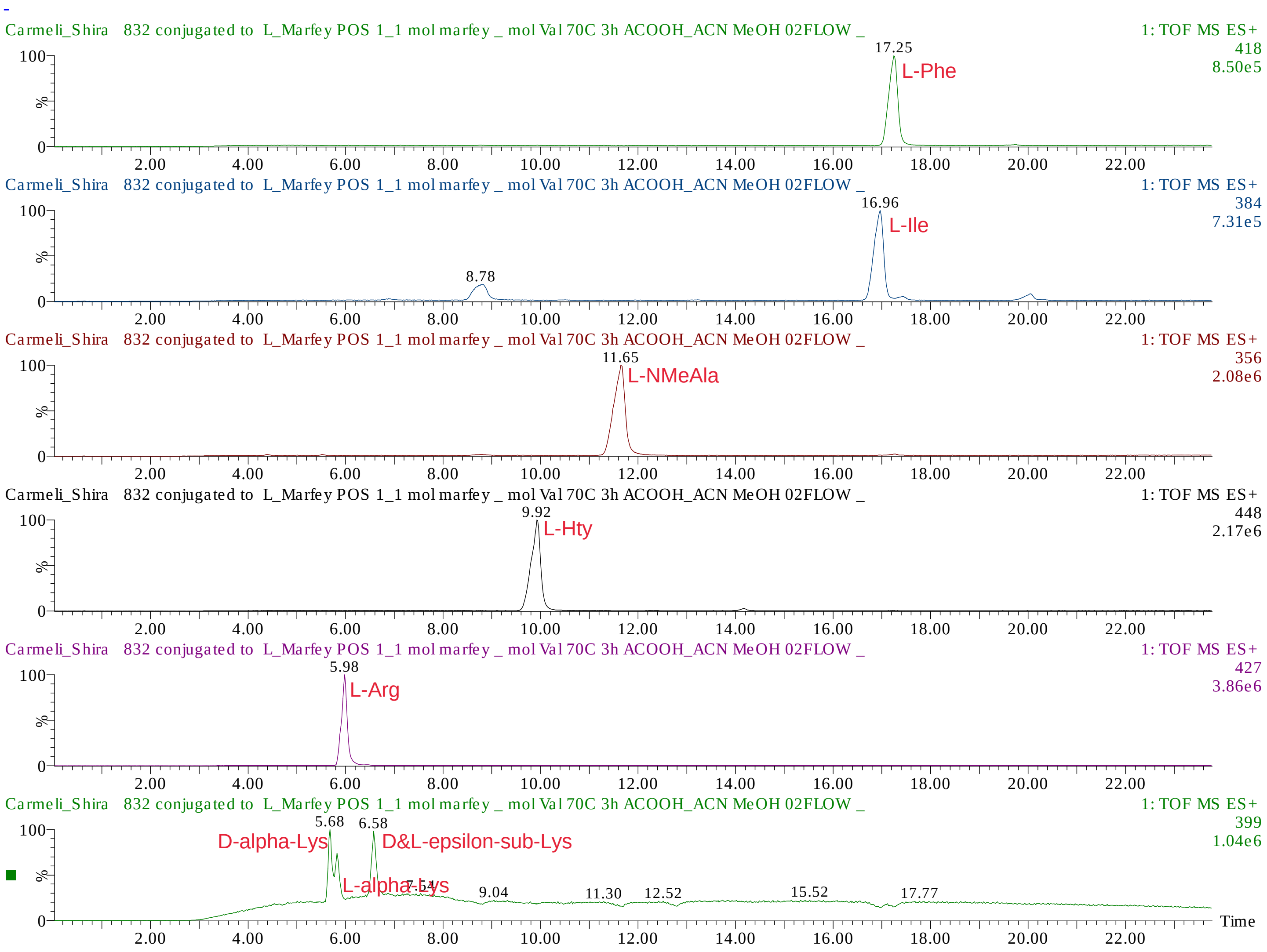

Table S3. NMR Data of Hydanto-anabaenopeptin F (**3**) in DMSO-d_6[a]

Position	δ_C, Mult.	δ_H, Mult. (J in Hz)[b]	HMBC correlations[c]	COSY correlations	TOCSY correlations	ROESY correlations
Arg-1	174.8, C<br>*174.7, C*		Arg-2, 2-NH, Lys-2			
Arg-2<br><br>*Arg-2*	56.0, CH	4.14, dd<br>4.9, 5.80<br>*4.18, dd*<br>*(4.9, 5.8)*		Arg-3a, 3b		Arg-2-NH, 3a, 3b
Arg-2-NH<br>*Arg-2-NH*		8.74, s<br>*8.75, s*				Arg-2, 4b
Arg-3a<br>Arg-3b<br>*Arg-3*	28.9, CH_2<br><br>*28.6, CH_2*	1.74, m<br>1.52, m	Arg-5	Arg-2	Arg-2, 5	Arg-2<br>Arg-2
Arg-4a<br>Arg-4b<br>*Arg-4*	24.2, CH_2<br><br>*23.9, CH_2*	1.50, m<br>1.42, m		Arg-6<br>Arg-6		
Arg-5	40.0, CH_2	3.05, brdt<br>(4.8, 6.2)		Arg-4a, 4b, 6-NH	Arg-4a, 4b	
Arg-6-NH		9.05, brs		Arg-5		
Arg-7	157.8, C					
Arg-NH_2, NH		8.03, brs				
CO<br><br>*CO*	156.9, C<br><br>*156.8, C*		Lys-2, Arg-2-NH			
Lys-1	168.4, C<br>*168.3, C*		Ile-2-NH, Lys-2			
Lys-2<br><br>*Lys-2*	56.5, CH<br><br>*56.3, CH*	4.32, dd<br>(11.4, 3.8)<br>*4.32, dd*	Arg-2-NH	Lys-3a, 3b		Lys-4a, 4b
Lys-3a<br><br>Lys-3a	29.6, CH_2	2.39, m<br><br>1.45, m	Lys- 4b	Lys-2, 4a, 4b<br>Lys-2, 4a, 4b	Lys-2, 4a, 4b,<br>Lys-2, 4a, 4b	Ile-2-NH<br><br>Ile-2-NH

Lys-3	*29.5, CH_2*					
Lys-4a<br>Lys-4b<br>*Lys-4*	23.1, CH_2<br><br>*23.0, CH_2*	1.45, m<br>1.19, m		Lys- 4b<br>Lys- 4a		Lys-2<br>Lys-2
Lys-5a<br>Lys-5b<br>*Lys-5*	28.3, CH_2<br><br>*28.2, CH_2*	1.51, m<br>1.46, m				Lys-6a
Lys-6a<br>Lys-6b	38.6, CH_2	3.53, m<br>2.66, m		Lys-6b, ε-NH<br>Lys-4b, 6a, ε-NH		Lys-4a, 6b, ε-NH,<br>Lys-6a, ε-NH
Lys-ε-NH<br><br>*Lys-ε-NH*		7.46, t<br>(5.3)<br>*7.45, t*<br>*(5.0)*		Lys-6a, 6b	Lys-5b, 6a, 6b	NMeAla-2, Phe-2,<br>Lys-5b, 6a, 6b
Ile-1	172.5, C		Hty-2-NH,<br>Ile-2			
Ile-2<br><br>*Ile-2*	56.4, CH<br><br>*56.2, CH*	4.26, dt<br>(8.0, 6.8)	Ile-6	Ile-2-NH, 3		Hty-2-NH,<br>Ile-2-NH, 3
Ile-2-NH<br><br>*Ile-2-NH*		7.89, d<br>(8.0)<br>*7.90, d*<br>*(8.0)*		Ile-2	Ile-2, 3, 6	Ile-2, 3, 6<br>Lys-3a, 4a
Ile-3	36.7, CH	1.85, m	Ile-2, 4b, 6	Ile-2, 4a, 4b, 6		Ile-2
Ile-4	25.6, CH_2	1.53, m<br>1.15, m	Ile-6	Ile-3, 4b, 6 Ile-3,<br>4a, 6		
Ile-5<br><br>*Ile-5*	11.8, CH_3<br><br>*11.7, CH_3*	0.89, t<br>(7.4)<br>*0.89, t*<br>*(7.4)*	Ile-4a,4b<br><br>*Ile-4a, 4b*	Ile-4a,4b<br><br>*Ile-4a, 4b*		
Ile-6<br>*Ile-6*	14.8, CH_3<br>*14.8, CH_3*	0.89, d<br>*0.89, d*	Ile-4a<br>*Ile-4a*			Ile-2-NH, Phe-6,6'<br>*Ile-2-NH, Phe-5,5', 6,6'*

Hty-1	171.1, C		NMeAla-2, NMe, Hty-3a, 3b			
Hty-2	49.0, CH	4.77, brdt (5.3, 6.5)	Hty-3b	Hty-2-NH, 3a, 3b,	Hty-2-NH, 3a, 3b, 4a, 4b	Hty-2-NH, 3a, 3b, Phe-2-NH, NMeAla-2
Hty-2-NH<br><br>*Hty-2-NH*		8.98, d (5.3)<br>*8.96, d (5.3)*		Hty-2	Hty-2	Hty-2, 3a, 3b, 4a, Ile-2
Hty-3a<br><br>Hty-3b	33.4, CH_2	1.85, m<br><br>1.74, m		Hty-2, 3b, 4a, 4b<br><br>Hty-2, 3b, 4a, 4b		Ile-2-NH, Hty-2, NH, 6,6’<br>Hty-2, NH, 6,6’
Hty-4a<br><br>Hty-4b	30.8, CH_2	2.62, m<br><br>2.42, m	Hty-3b, 6,6’	Hty-3a, 3b, 4b<br>Hty-3a, 3b, 4a		Hty-2-NH, 6,6’<br>Hty-6,6’
Hty-5	131.0, C		Hty-4b, 7,7’			
Hty-6,6’	129.1, CH x2	6.98, d x2 (8.5)	Hty-6’,6, 7,7’	Hty-7,7’		Hty-3a, 3b, 4a, 4b, NMeAla-3
Hty-7,7’	115.4, CH x2	6.66, d x2 (8.5)	Hty-7’,7	Hty-6,6’		
Hty-8	155.9, C		Hty-6,6’, 7,7’			
NMeAla-1	170.0, C		Phe-2-NH, NMeAla-2, 3			
NMeAla-2	54.6, CH	4.88, dq (2.6, 7.0)	NMeAla-3, NMe	NMeAla-3		Phe-2-NH, Lys-ε-NH, NMeAla-3, NMe, Hty-2
NMeAla-3	14.1, CH_3	1.07, d (7.0)	NMeAla-2	NMeAla-2		Hty-6,6’
NMeAla-NMe<br><br>*NMeAla-NMe*	27.3, CH_3	1.81, s<br><br>*1.81, s*	NMeAla-2			Phe-2-NH, 5,5’, 6,6’
Phe-1	171.3, C		Lys-ε-NH, Phe-3b			

Phe-2	55.2, CH	4.35, m	Phe-3b	Phe-2-NH, 3a, 3b	Phe-2-NH, 3a, 3b	Phe-2-NH, 3a, 5, 5’, Lys-ε-NH
Phe-2-NH<br><br>*Phe-2-NH*		8.65, d (8.6)<br>*8.66, d (8.6)*		Phe-2	Phe-2, 3a, 3b	NMeAla-2, NMe, Phe-2, 3a, 3b<br>Hty-2
Phe-3a<br><br>Phe-3b<br><br>*Phe-3b*	37.7, CH_2	3.23, dd (2.6, 13.2)<br>2.84, dd (4.8, 13.2)<br>*2.81, dd (4.8, 13.2)*		Phe-3b<br><br>Phe-3a		Phe-2, 3b, 5, 5’<br>Phe-2-NH, 3b, 5, 5’, 6, 6’
Phe-4	138.5, C		Phe-3b, 6, 6’			
Phe-5,5’	129.1, CH x2	7.07, d x2 (7.2)	Phe-5’, 5, 7	Phe-6,6’		Phe-2, 3a, 3b, NMeAla- NMe, Ile-4a, 6
Phe-6,6’	128.4, CH x2	7.19, dd x2 (6.9, 7.2)	Phe-6’,6	Phe-5’,5, 7		Phe-3b, NMeAla-NMe, Ile-4a, 6
Phe-7	126.3, CH	7.14, t (6.9)	Phe-5’,5	Phe-6,6’		

[a]500 MHz for ^{1}H and 125 MHz for ^{13}C, ca. 1:*1* major rotamer, *minor rotamer*. [b]From HSQC correlations. [c]J_{C-H}=8 Hz.

Figure S36. LCMS Chromatogram and Mass spectrum of Hydanto-anabaenopeptin F (**3**)

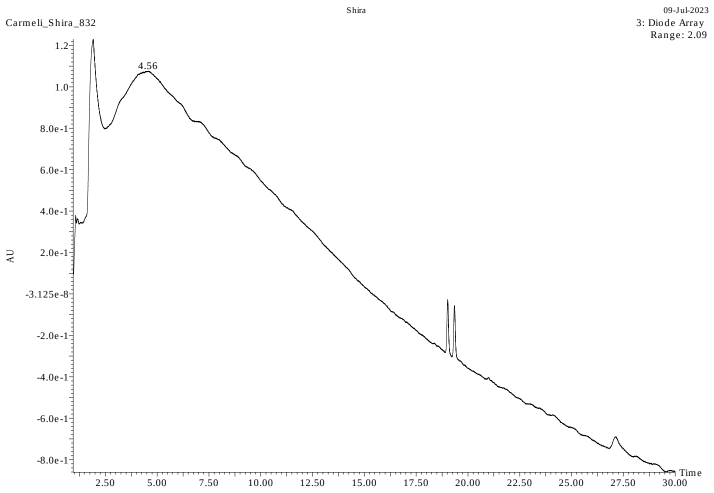

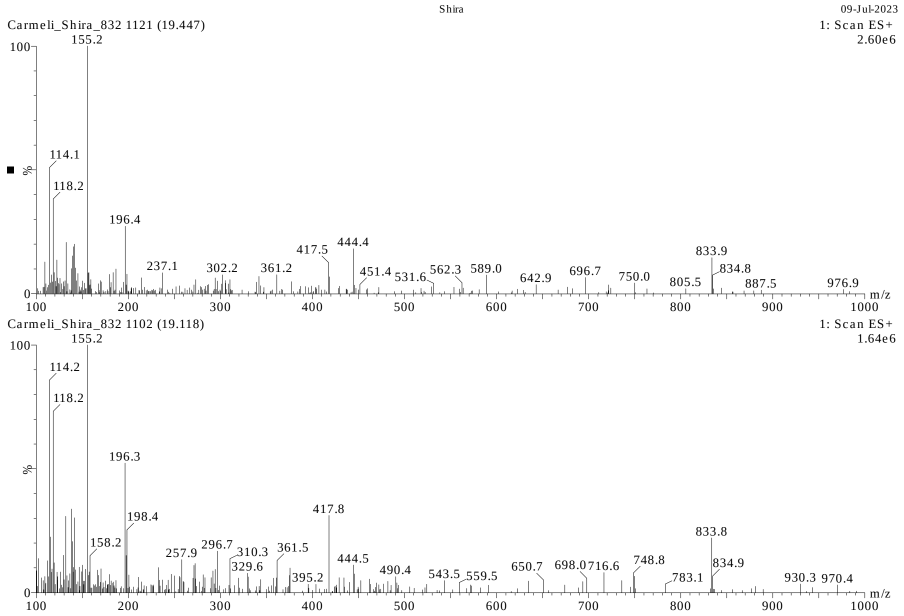

Figure S37. ^{1}H NMR Spectrum of Hydanto-oscillamide Y (**4**) in DMSO-d_6

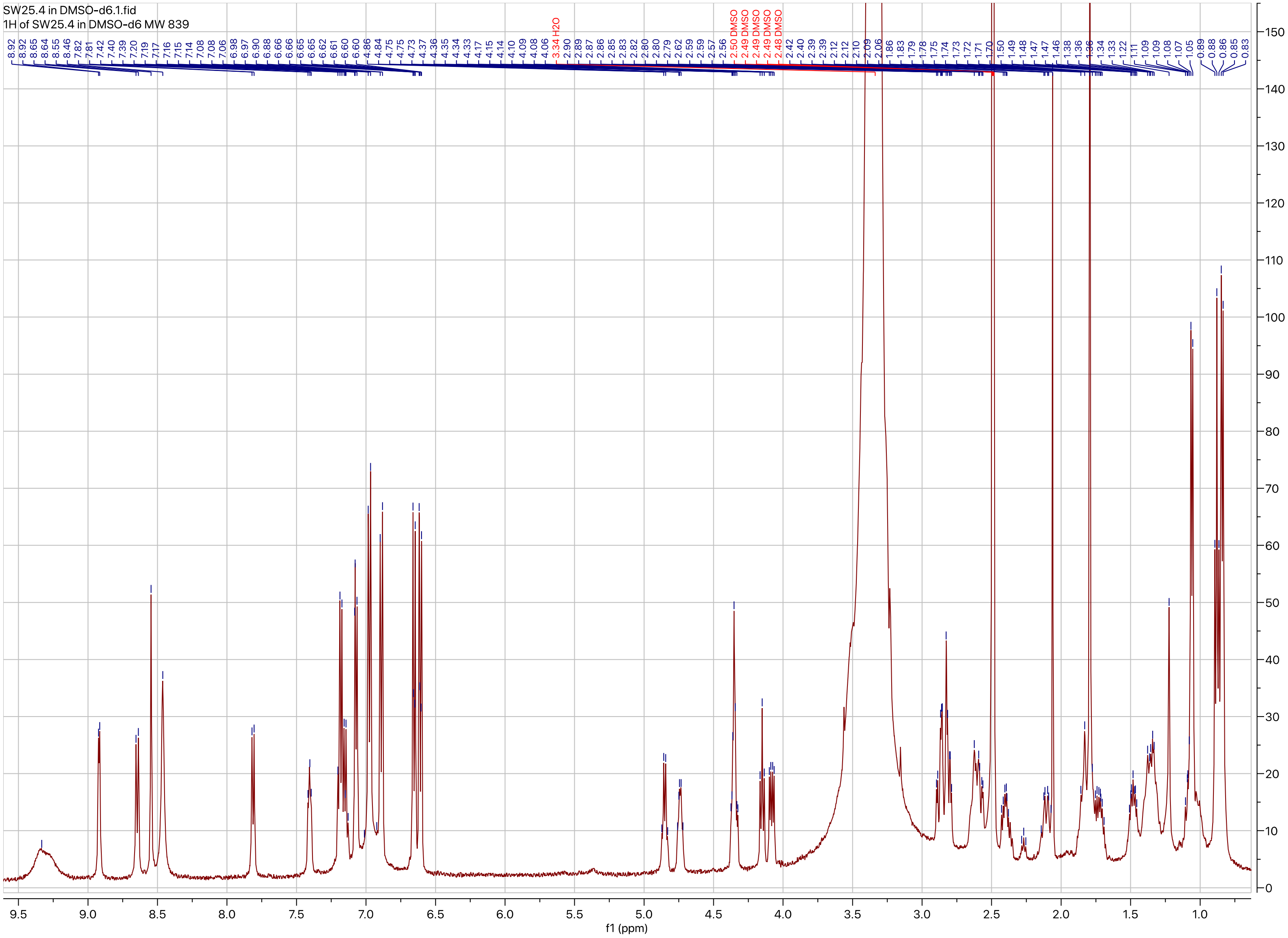

Figure S38. ^{13}C NMR Spectrum of Hydanto-oscillamide Y (**4**) in DMSO-d_6

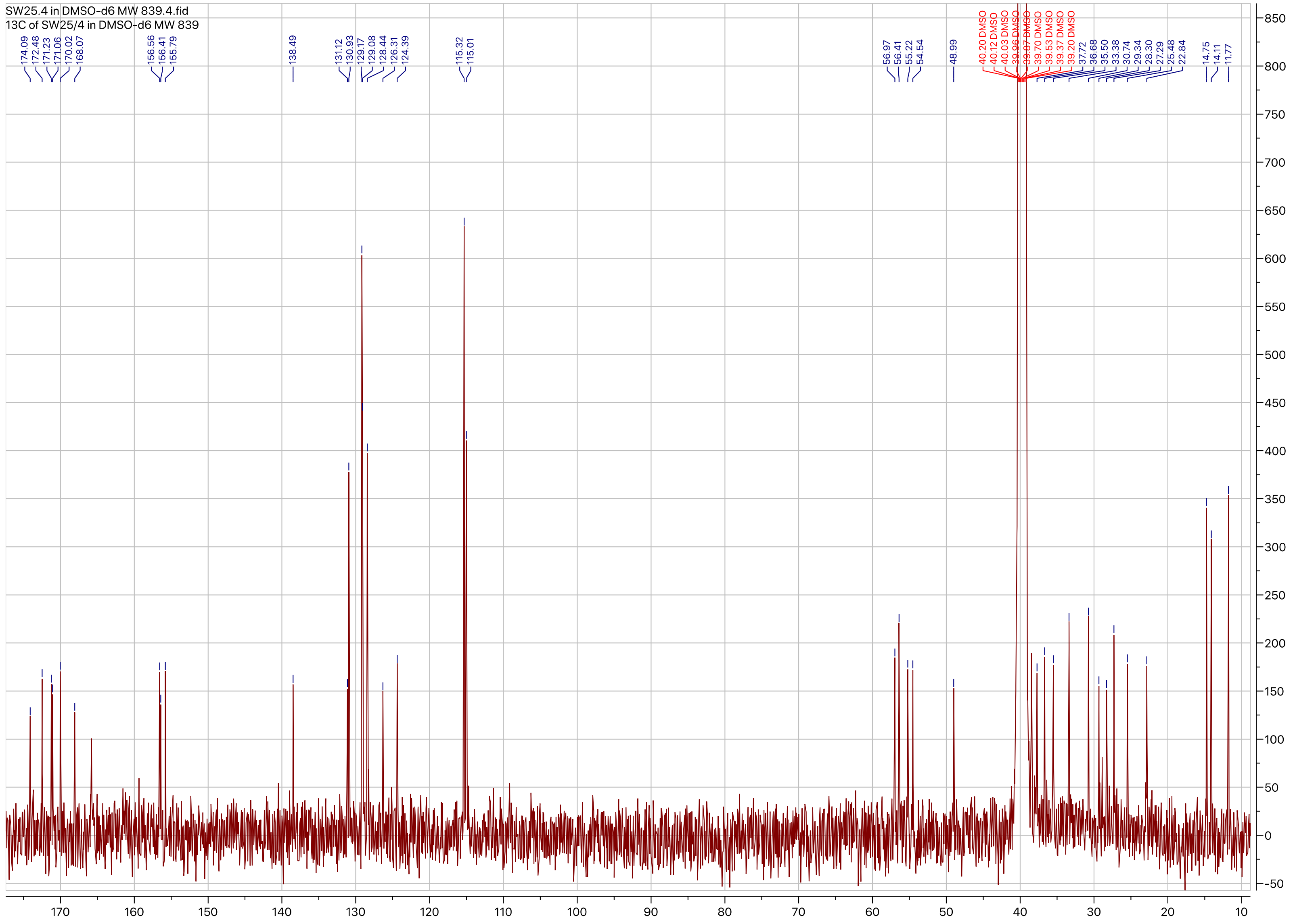

Figure S39. HSQC Spectrum of Hydanto-oscillamide n Y (**4**) in DMSO-d_6

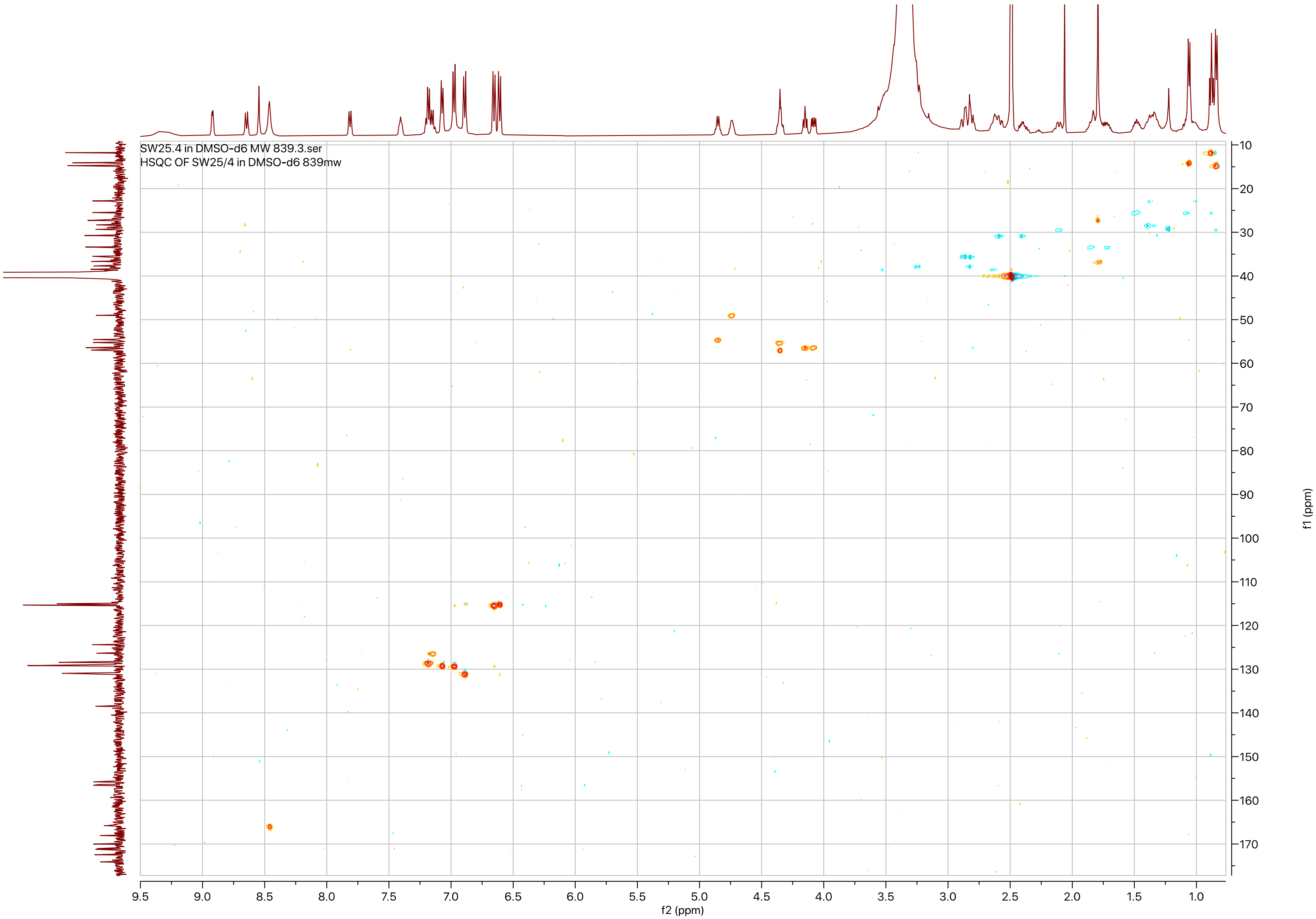

Figure S40. HMBC Spectrum of Hydanto-oscillamide Y (**4**) in DMSO-d_6

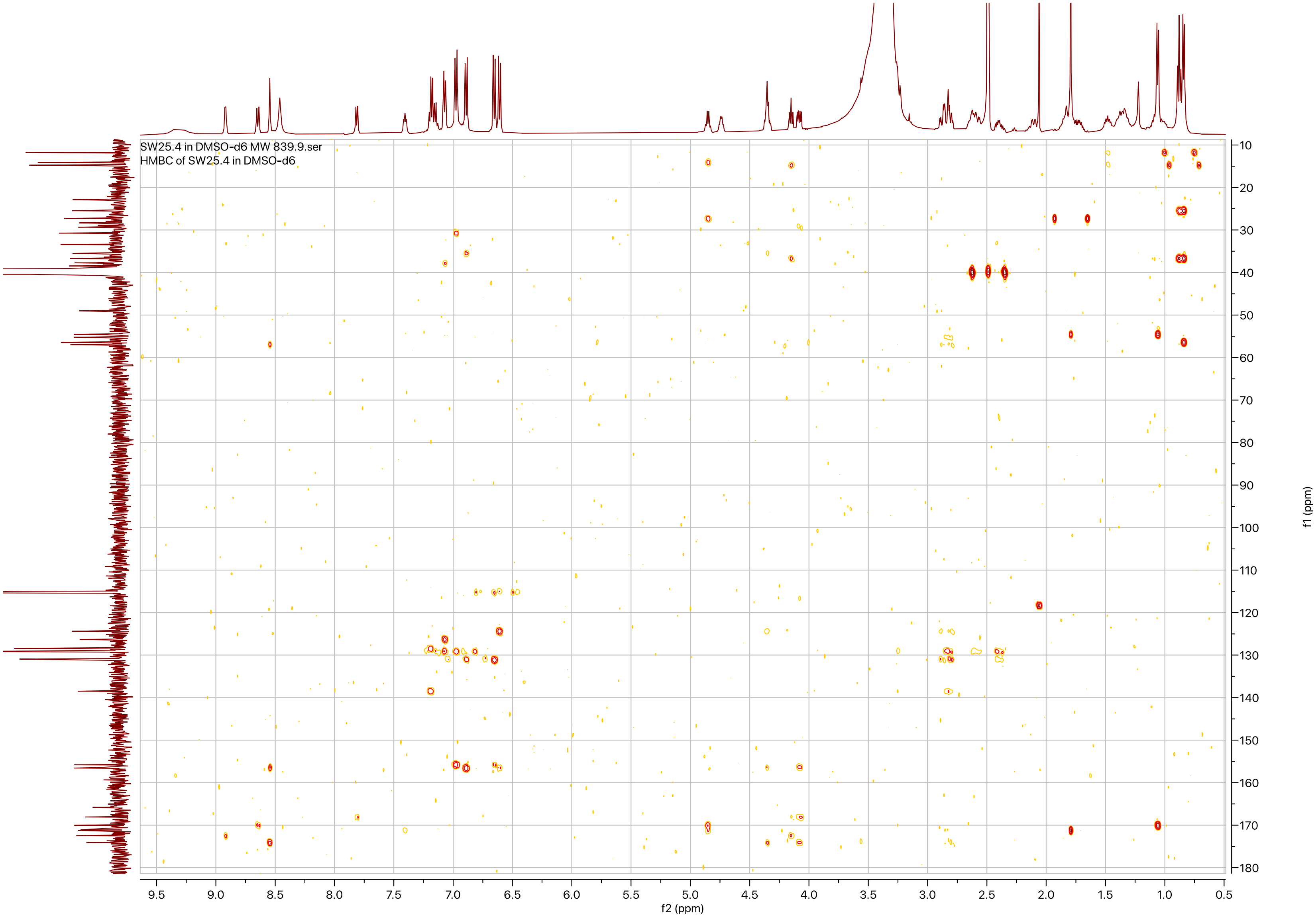

Figure S41. COSY Spectrum of Hydanto-oscillamide Y (**4**) in DMSO-d_6

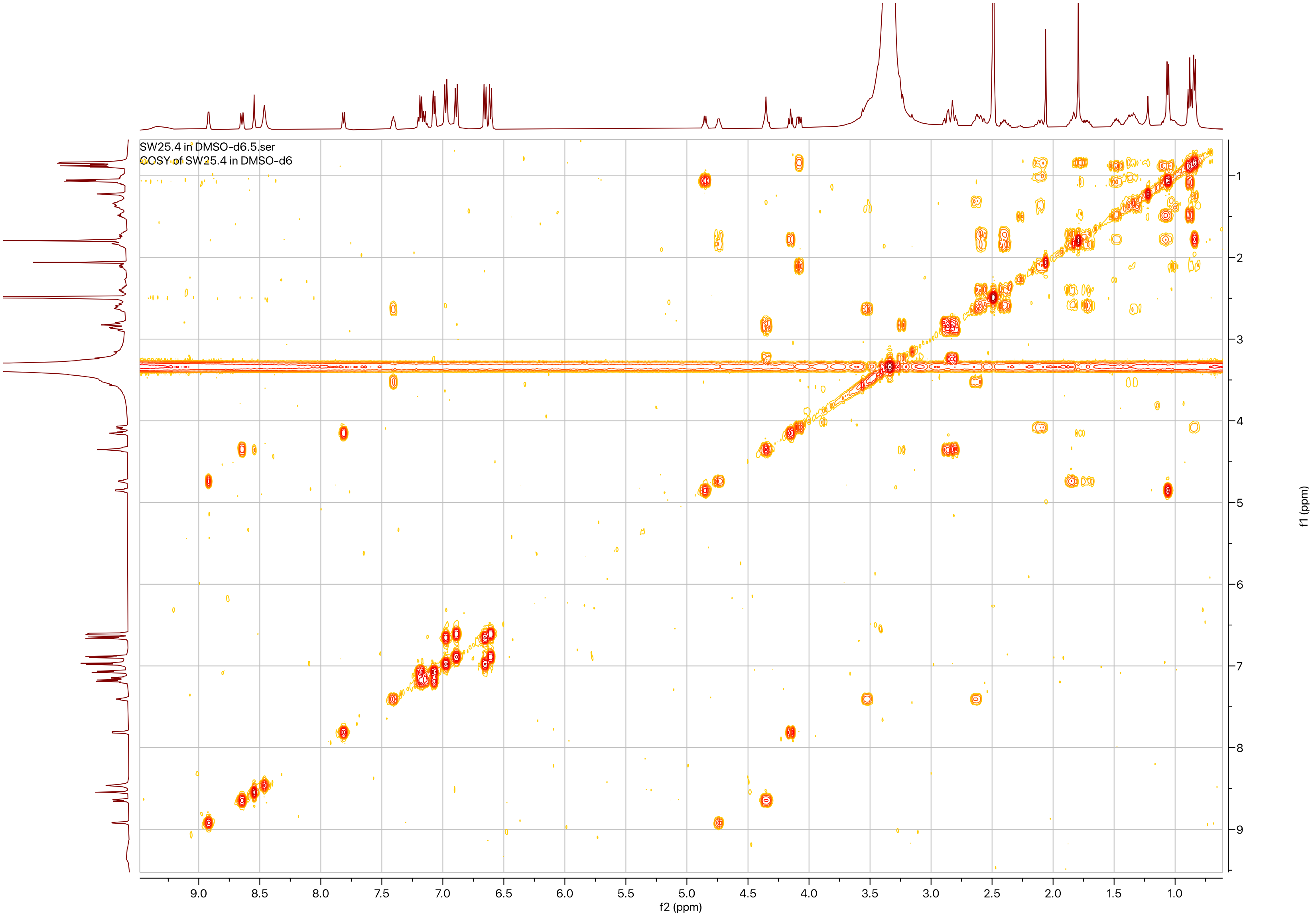

Figure S42. TOCSY Spectrum of Hydanto-oscillamide Y (**4**) in DMSO-d_6

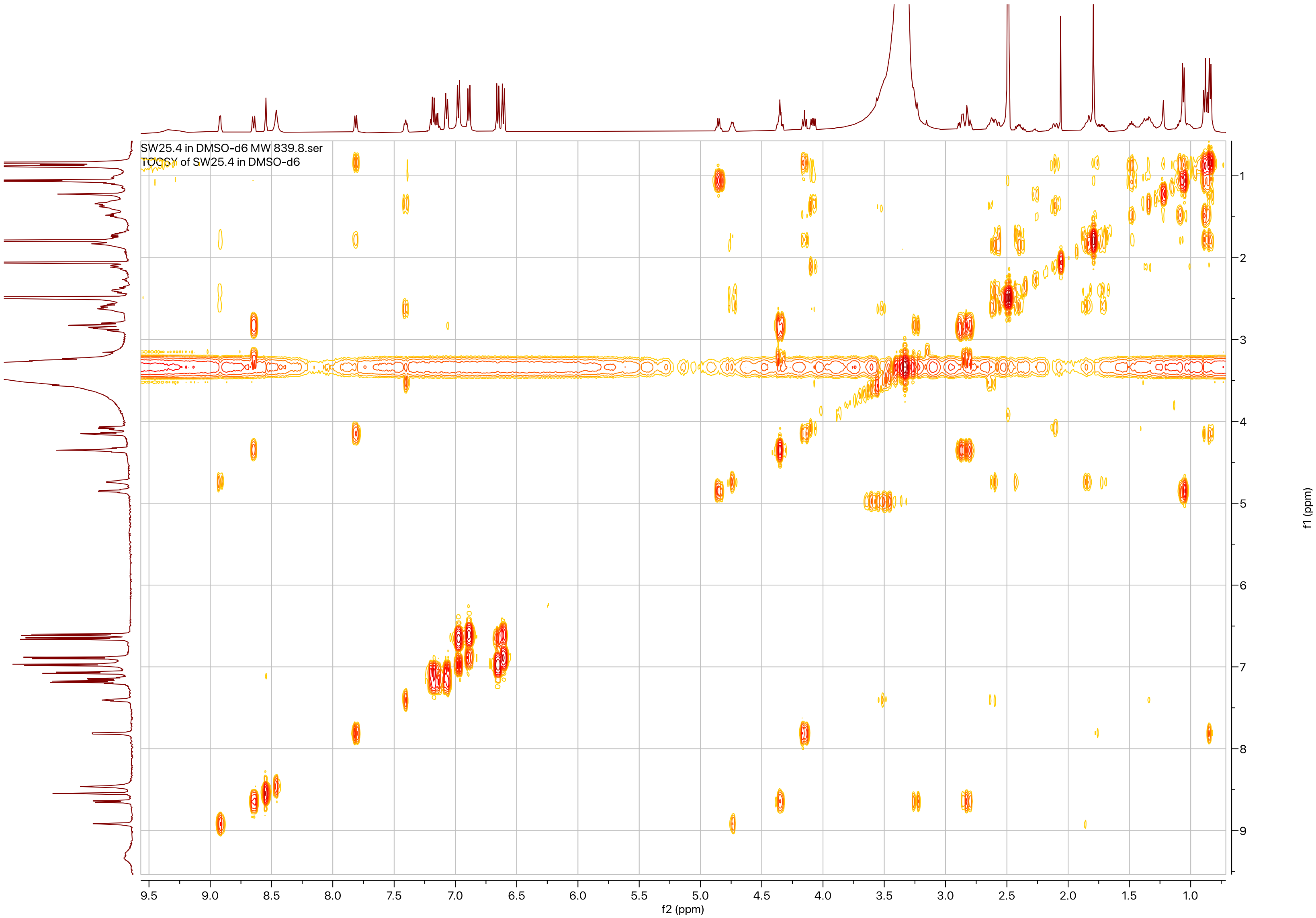

Figure S43. ROESY Spectrum of Hydanto-oscillamide Y (**4**) in DMSO-d_6

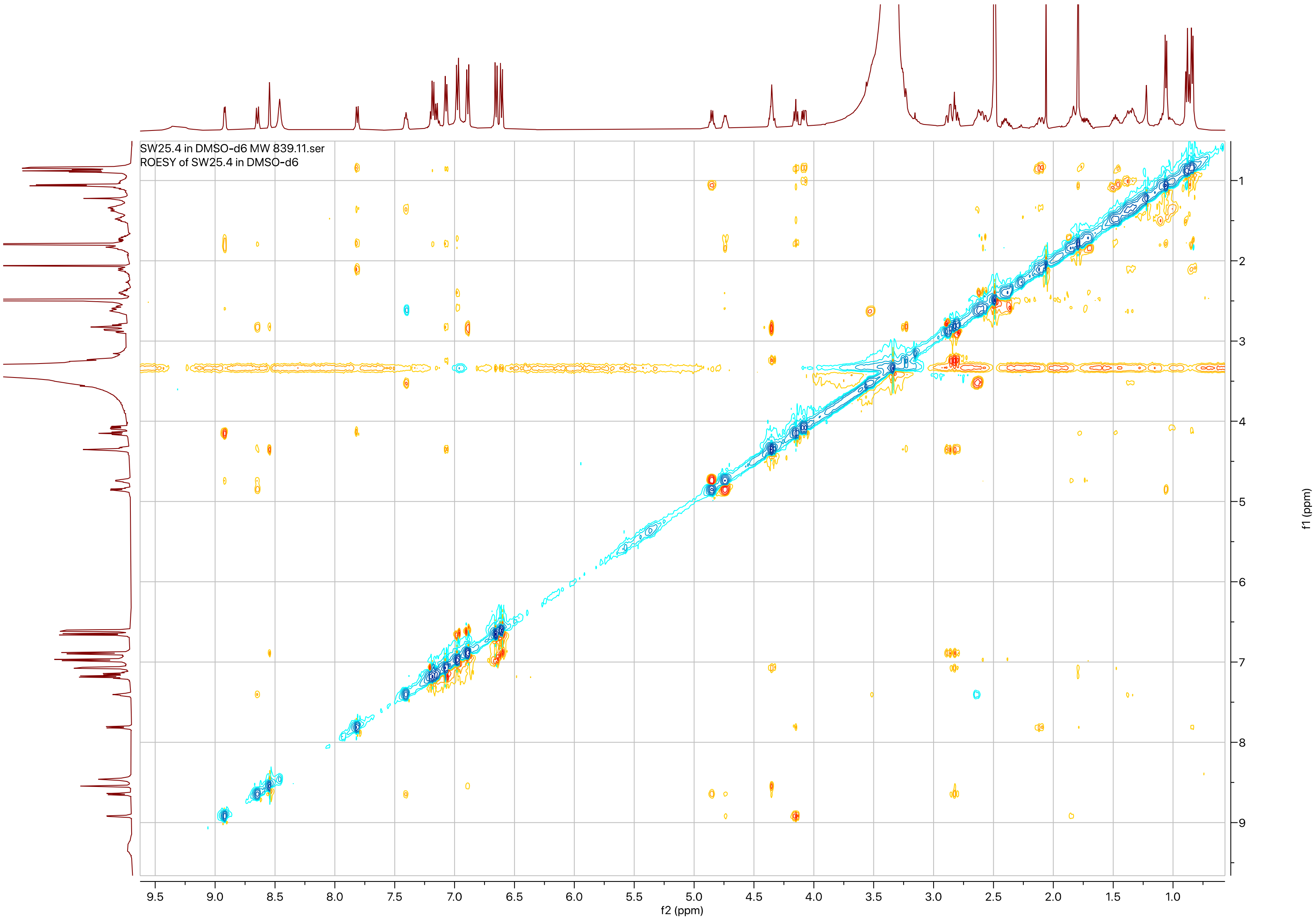

Figure S44. Negative HR ESI MS of Hydanto-oscillamide Y (**4**)

Single Mass Analysis

Tolerance = 3.0 PPM / DBE: min = -1.5, max = 50.0

Element prediction: Off

Number of isotope peaks used for i-FIT = 3

Monoisotopic Mass, Odd and Even Electron Ions

692 formula(e) evaluated with 5 results within limits (all results (up to 1000) for each mass)

Elements Used:

Mass	Calc. Mass	mDa	PPM	DBE	Formula	i-FIT	i-FIT Norm	Fit Conf %	C	H	N	O
838.4143	838.4140	0.3	0.4	21.5	C45 H56 N7 O9	506.9	0.920	39.84	45	56	7	9
	838.4139	0.4	0.5	27.0	C44 H50 N14 O4	507.7	1.736	17.61	44	50	14	4
	838.4153	-1.0	-1.2	26.5	C46 H52 N11 O5	508.5	2.580	7.58	46	52	11	5
	838.4126	1.7	2.0	22.0	C43 H54 N10 O8	507.0	1.063	34.53	43	54	10	8
	838.4166	-2.3	-2.7	26.0	C48 H54 N8 O6	511.4	5.426	0.44	48	54	8	6

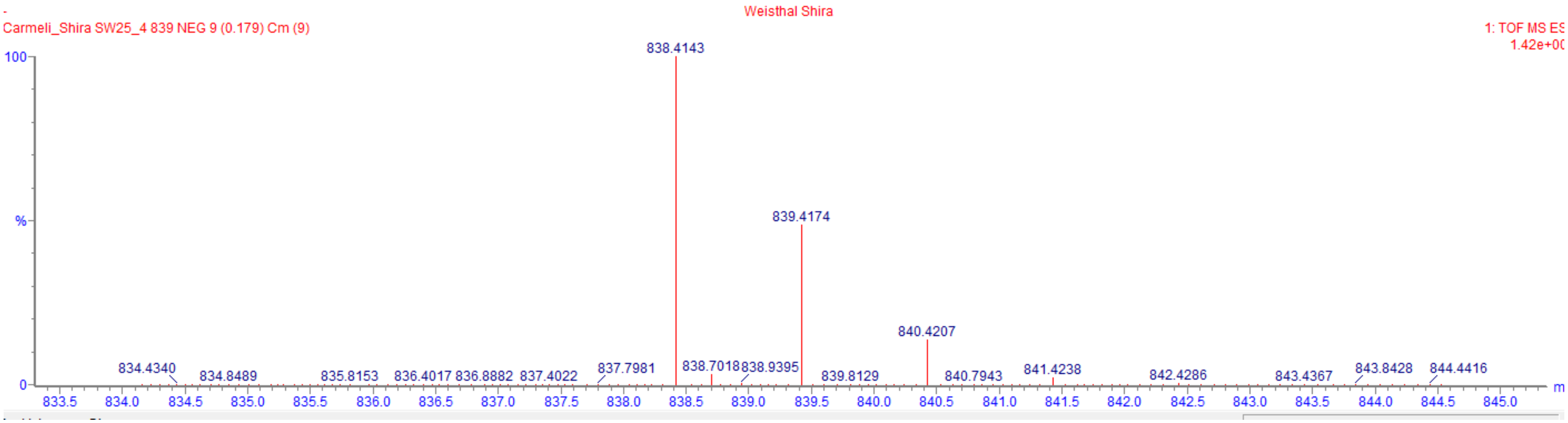

Figure S45. Positive HR ESI MS/MS Spectrum of Hydanto-oscillamide Y (**4**)

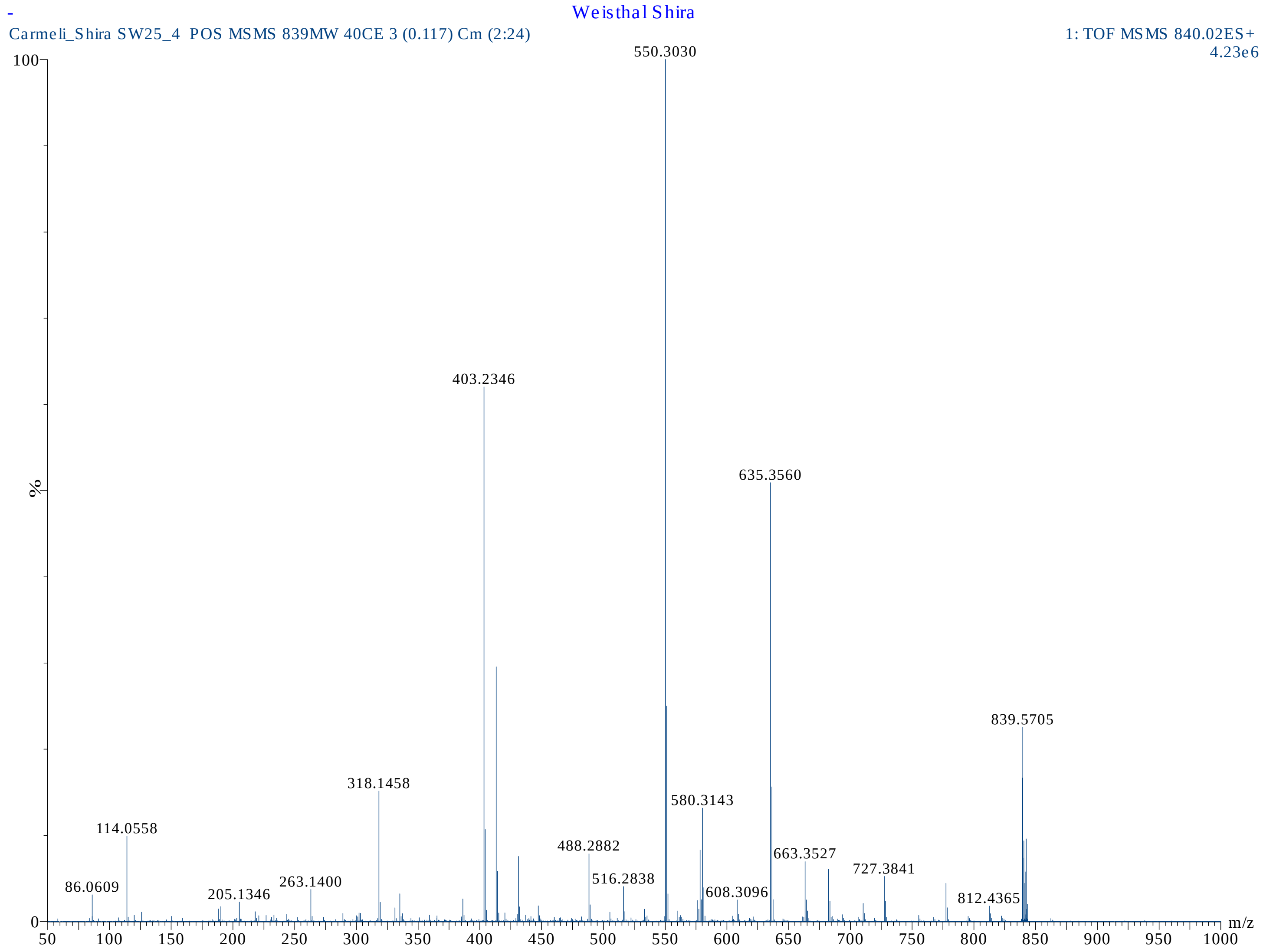

Figure S46. Scheme of Fragmentation in the Positive HR ESI MS/MS Spectrum of Hydanto-oscillamide Y (**4**)

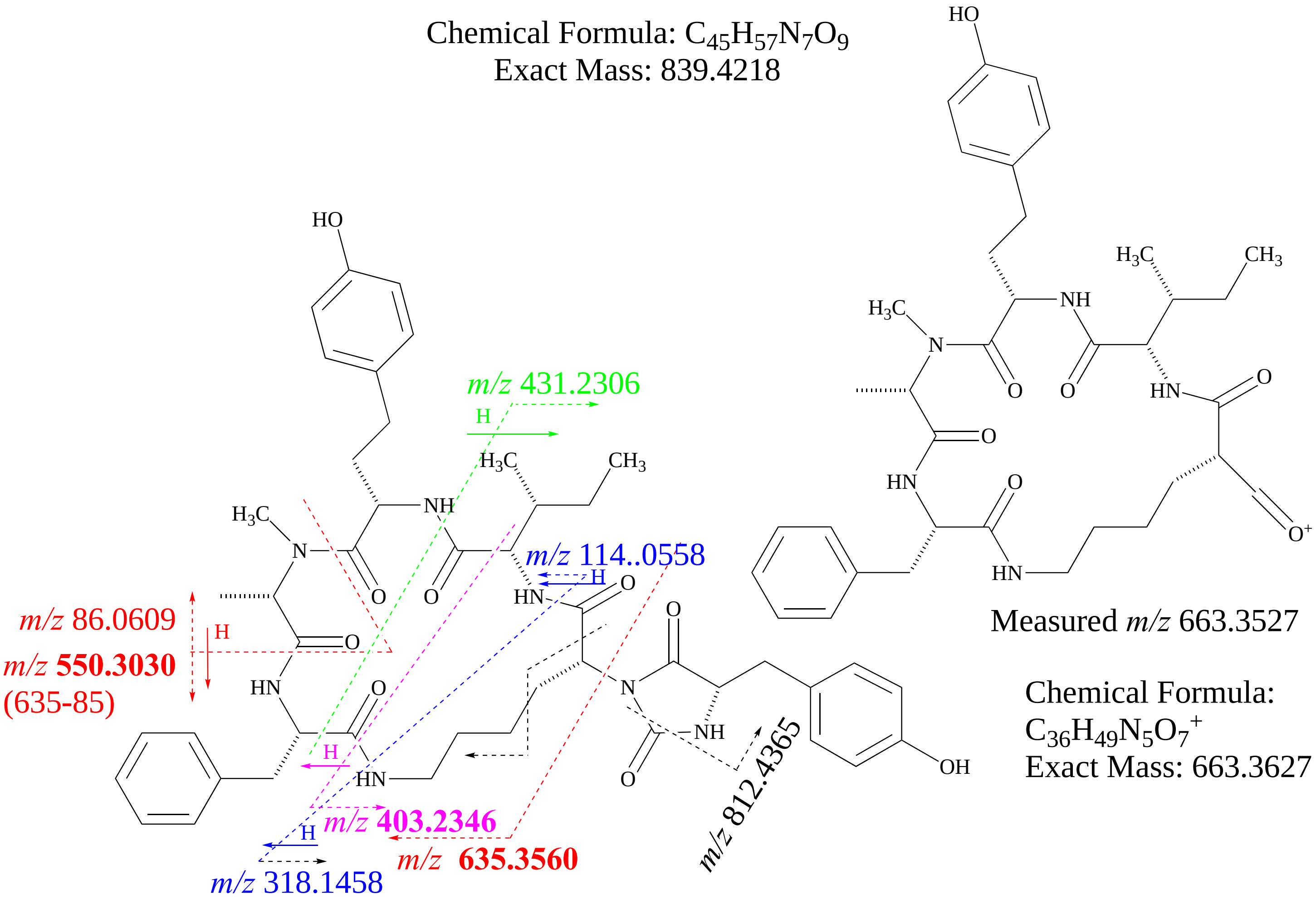

Figure S47. LCMS Traces of Marfey’s Method Amino Acids Analysis of Hydanto-oscillamide Y (**4**)

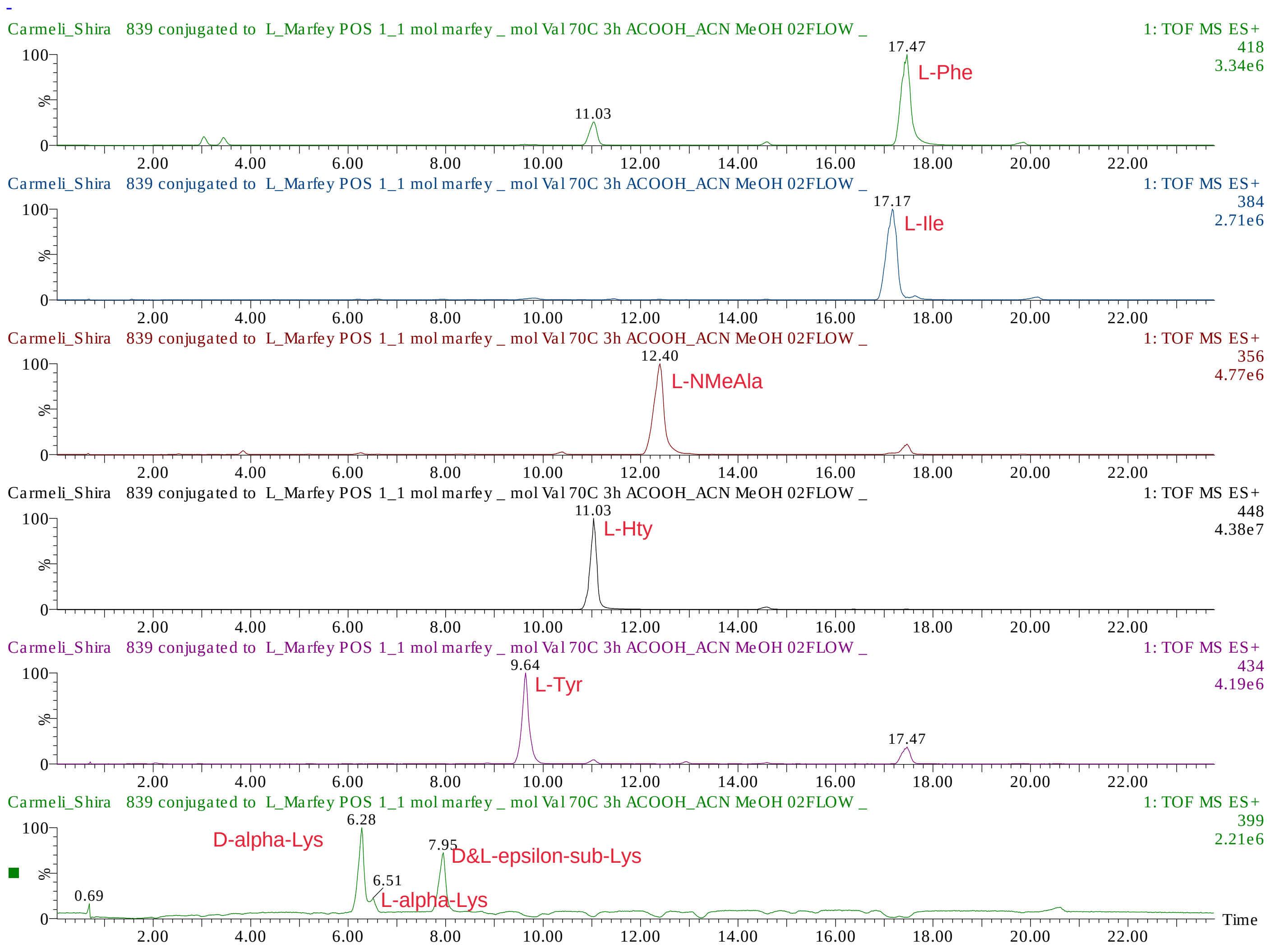

Table S4. NMR Data of Hydanto-oscillamide Y (**4**) in DMSO-d_6[a]

Position	δ_C, Mult.	δ_H, Mult. (J in Hz)[b]	HMBC correlations[c]	COSY correlations	TOCSY correlations	ROESY correlations
Tyr-1	174.1, C		Tyr-2,2-NH, 3b, Lys-2			
Tyr-2	57.0, CH	4.35, brt (4.5)	Tyr-2-NH,3a,3b	Tyr-2-NH,3a,3b	Tyr-2-NH,3a,3b	Tyr-2-NH,3a,3b
Tyr-2-NH		8.55, s		Tyr-2	Tyr-2,3a, 3b	Tyr-2,3b, 5,5'
Tyr-3a<br><br>Try-3b	35.5, CH_2	2.88, dd (14.4, 4.1)<br>2.82, m	6.89, 4.35	4.35, 2.82<br><br>4.35, 2.88	Tyr-2,3b<br><br>Tyr-2,3a	Tyr-2<br>Tyr-2,2-NH
Tyr-4	124.4, C		Tyr-2,3a, 3b,6,6'			
Tyr-5,5'	130.9, CH	6.89, d x2 (8.2)	Tyr-3a,3b, 5',5	Tyr-6,6'		Tyr-2-NH, 6,6'
Tyr-6,6'	115.0, CH	6.61, d x2 (8.2)	Tyr-6',6	Tyr-5,5'		Tyr-5,5'
Tyr-7	156.6, C		Tyr-5,5', 6,6'			
Tyr-7-OH*		9.33, brs				
CO	156.4, C		Tyr-2,2-NH, Lys-2			
Lys-1	168.1, C		Ile-2,2-NH, Lys-2			
Lys-2	56.4, CH	4.08, dd (11.6, 5.2)		Lys-3a, 3b	Lys-3a, 5a, 5b	Ile-2-NH
Lys-3	29.3, CH_2	2.10, brq (11.6)<br>0.86, m	Lys-2	Lys-2, 3b, 4a, 4b<br>Lys-2, 3a	Lys-2, 3b, 4a, 4b<br>Lys-2, 3a	Ile-2-NH
Lys-4	22.8, CH_2	1.36, m<br><br>1.00, m		Lys-3a, 3b, 4b<br>Lys-3a, 3b, 4a		
Lys-5	28.3, CH_2	1.39, m<br>1.34, m		Lys-6a<br>Lys-6a, 6b		Lys-6-NH<br>Lys-6-NH
Lys-6	38.5, CH_2	3.53, m<br><br>2.63, m		Lys-4a, 4b, 6b, 6-NH<br>Lys-4a,4b, 6a	Lys-5a, 6b, 6-NH<br>Lys-5a,6a, 6-NH	Lys-6-NH<br><br>Lys-6-NH
Lys-6-NH		7.41, dd (6.4,5.2)		Lys-6a,6b	Lys-3a, 3b, 6a, 6b	Lys-5a, 5b, 6a,6b(-), Phe-2, 2-NH
Ile-1	172.5, C		Hty-2-NH, Ile-2			

Ile-2	56.4, CH	4.15, t (7.4)	Ile-6	Hty-2-NH, 3	Hty-2-NH, 3, 4a, 5, 6	Ile-2-NH, Hty-2-NH
Ile-2-NH		7.82, d (7.4)		Hty-2	Hty-2, 3, 4b, 6	Lys-2, 3a, Ile-2, 3, 6
Ile-3	36.7, CH	1.79, m	Ile-2	Ile-2, 4a, 4b, 6	Ile-2, 4a, 4b, 5, 6	Ile-2-NH
Ile-4	25.5, CH_2	1.48, ddd (12.8,7.2, 4.4)<br>1.08, m	Ile-5,6	Ile-3, 4b, 5<br><br>Ile-3, 4b, 5	Ile-2, 3, 4b, 5, 6<br>Ile-2, 3, 4a, 5, 6	Phe-5,5’
Ile-5	11.8, CH_3	0.88, t (7.4)	Ile-4a	Ile-4a,4b		
Ile-6	14.8, CH_3	0.84, d (6.7)	Ile-2,4a	Ile-3		Ile-2-NH
Hty-1	171.1, C		NMe-Ala-2, NMe, Hty-3b			
Hty-2	49.0, CH	4.74, brdt (5.0,6.5)		Hty-3a,3b	Hty-3a,3b, 4a,4b	Hty-2-NH, NMe-Ala-2
Hty-2-NH		8.92, d (4.3)		Hty-2	Hty-2,3a, 3b,4a,4b	Ile-2, Phe-2-NH, Hty-2,3a,3b
Hty-3	33.4, CH_2	1.85, m<br><br>1.72, m	Hty-2	Hty-2,3b, 4a,4b<br>Hty-2,3a, 4a,4b	Hty-2,3b, 4a,4b<br>Hty-2,3a, 4a,4b	Hty-2-NH, 6,6’<br>Hty-2-NH, 6,6’
Hty-4	30.7, CH_2	2.59, ddd (13.8,12.0, 4.2)<br>2.40, ddd (13.8,11.5, 6.3)	Hty-6,6’	Hty-3a,3b, 4b<br><br>Hty-3a,3b, 4a	Hty-2,3a, 3b,4b<br><br>Hty-2,3a, 3b,4a	Hty-6,6’<br><br><br>Hty-6,6’
Hty-5	131.1, C		Hty-4b,7,7’			
Hty-6,6’	129.2, CH	6.97, d x2 (8.5)	Hty-4a,4b, 6’,6			Hty-3a,3b, 4a,4b,7,7’
Hty-7,7’	115.3, CH	6.65, d x2 (8.5)	Hty-7’,7			Hty-6,6’
Hty-8	155.8, C		Hty-6,6’, 7,7’			
OH*		9.27, brs				
NMe-Ala-1	170.0, C		Phe-2,2-NH, NMe-Ala-2,3			
NMe-Ala-2	54.5, CH	4.85, q (6.6)	NMe-Ala-3, NMe	NMe-Ala-3	NMe-Ala-3	Hty-2, Phe-2-NH
NMe-Ala-3	14.1, CH_3	1.06, d (6.6)	NMe-Ala-2	NMe-Ala-2	NMe-Ala-3	

NMe-Ala-NMe	27.3, CH_3	1.79, s	NMe-Ala-2			Phe-2-NH, 5,5’
Phe-1	171.2, C		Lys-6-NH, Phe-3			
Phe-2	55.2, CH	4.35, m	Phe-3	Phe-2-NH, 3a,3b	Phe-2-NH, 3a,3b	Phe-2-NH, 3a,3b, Lys-6-NH
Phe-2-NH		8.65, d (8.5)		Phe-2	Phe-2,3a, 3b	Lys-6-NH, NMe-Ala-2, NMe, Phe-2,3b
Phe-3	37.7, CH_2	3.24, dd (13.8,3.2)<br>2.82, m	Phe-5,5’	Phe-2,3b<br>Phe-2,3a	Phe-2,3b<br>Phe-2,3a	Phe-2,5,5’<br>Phe-2, NH, 5,5’
Phe-4	138.5, C		Phe-3a,3b<br>6,6’			
Phe-5,5’	129.1, CH	7.07, d x2 (7.0)	Phe-3a,3b, 5’,5			Phe-3a,3b, 6,6’, NMe-Ala-NMe, Ile-4
Phe-6,6’	128.4, CH	7.19, t x2 (7.0)	Phe-6’,6			Phe-5,5’
Phe-7	126.3, CH	7.16, t (7.0)	Phe-5,5’			

[a]500 MHz for ^1H and 125 MHz for ^{13}C. [b]From HSQC correlations. [c]J_{C-H}=8 Hz. *May interchange.

Figure S48. LCMS Chromatogram and Mass spectrum of Hydanto-oscillamide Y (**4**)

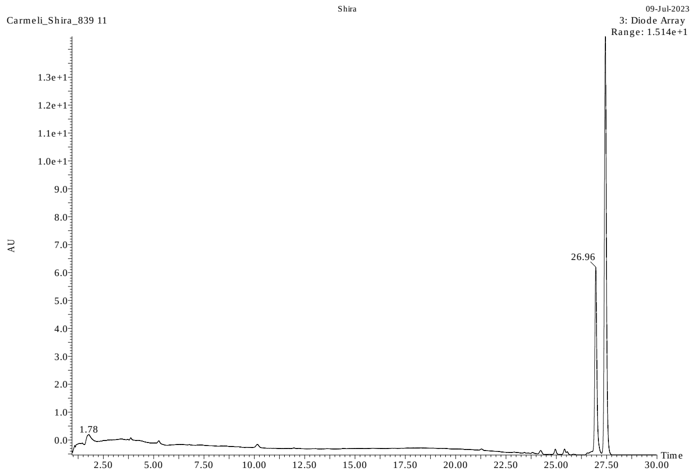

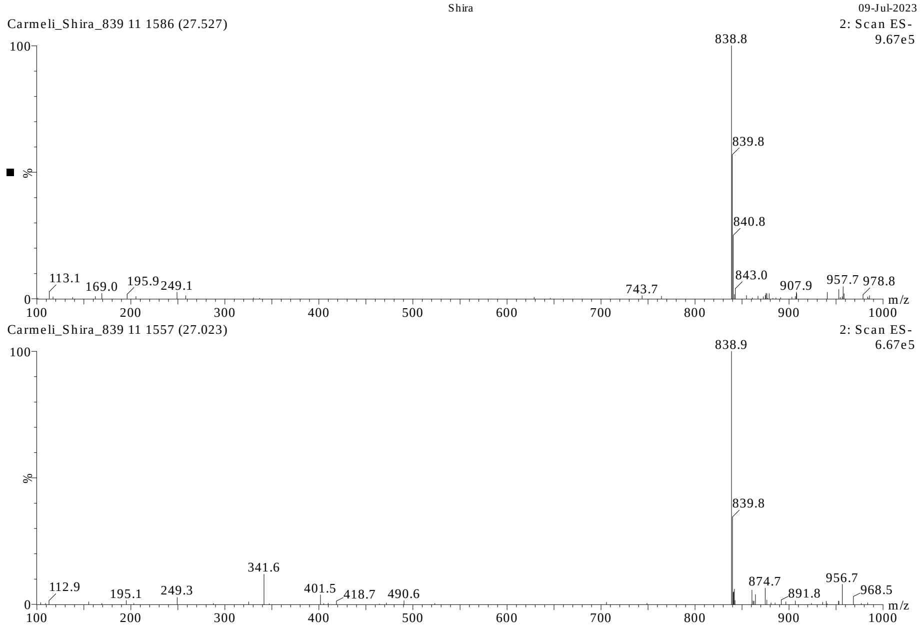

Figure S49. ^{1}H NMR Spectrum of [1][Dht]-Anabaenopeptin A (**5**) in DMSO-d_6

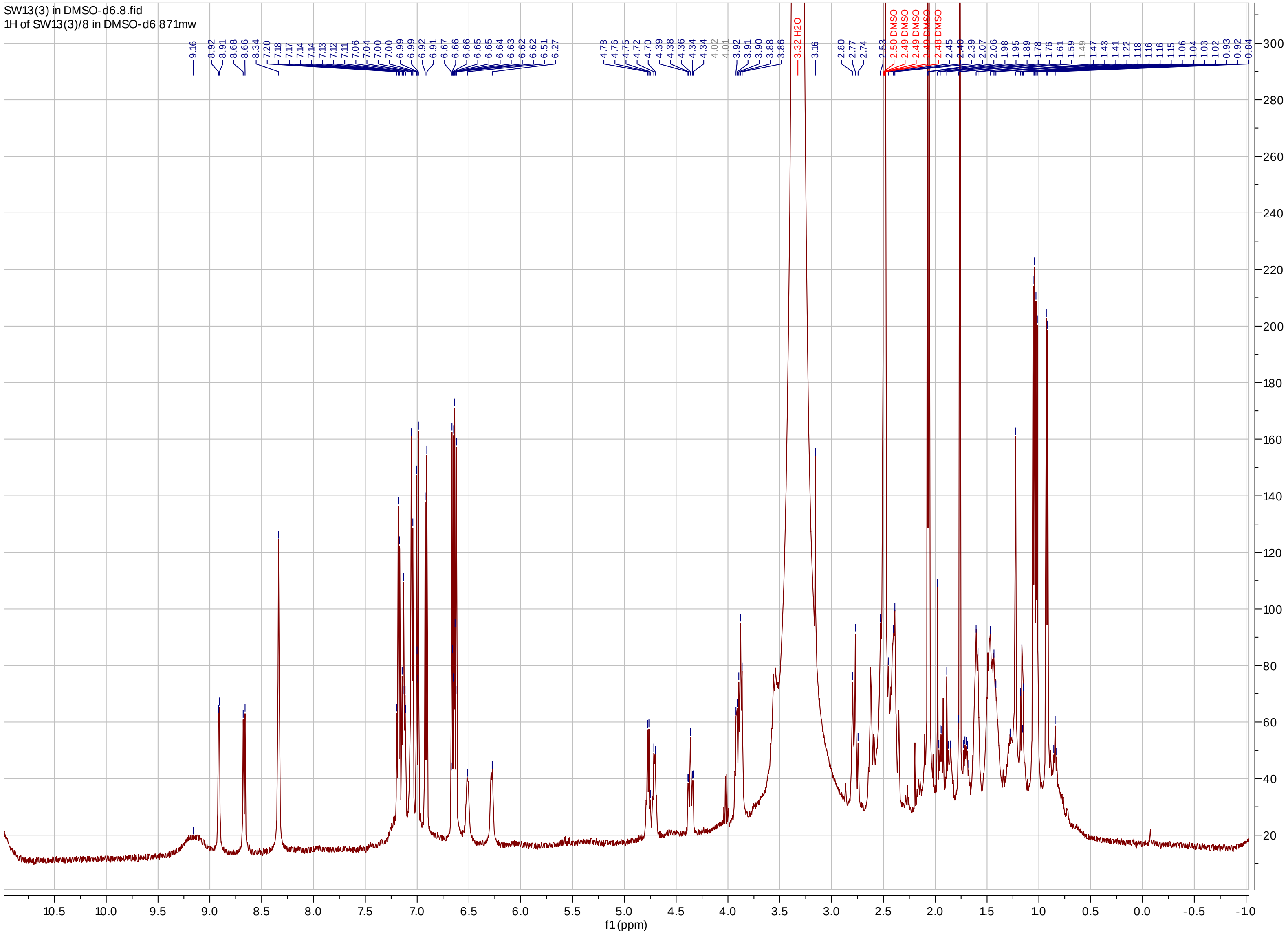

Figure S50. ^{13}C NMR Spectrum of [1][Dht]-Anabaenopeptin A (**5**) in DMSO-d_6

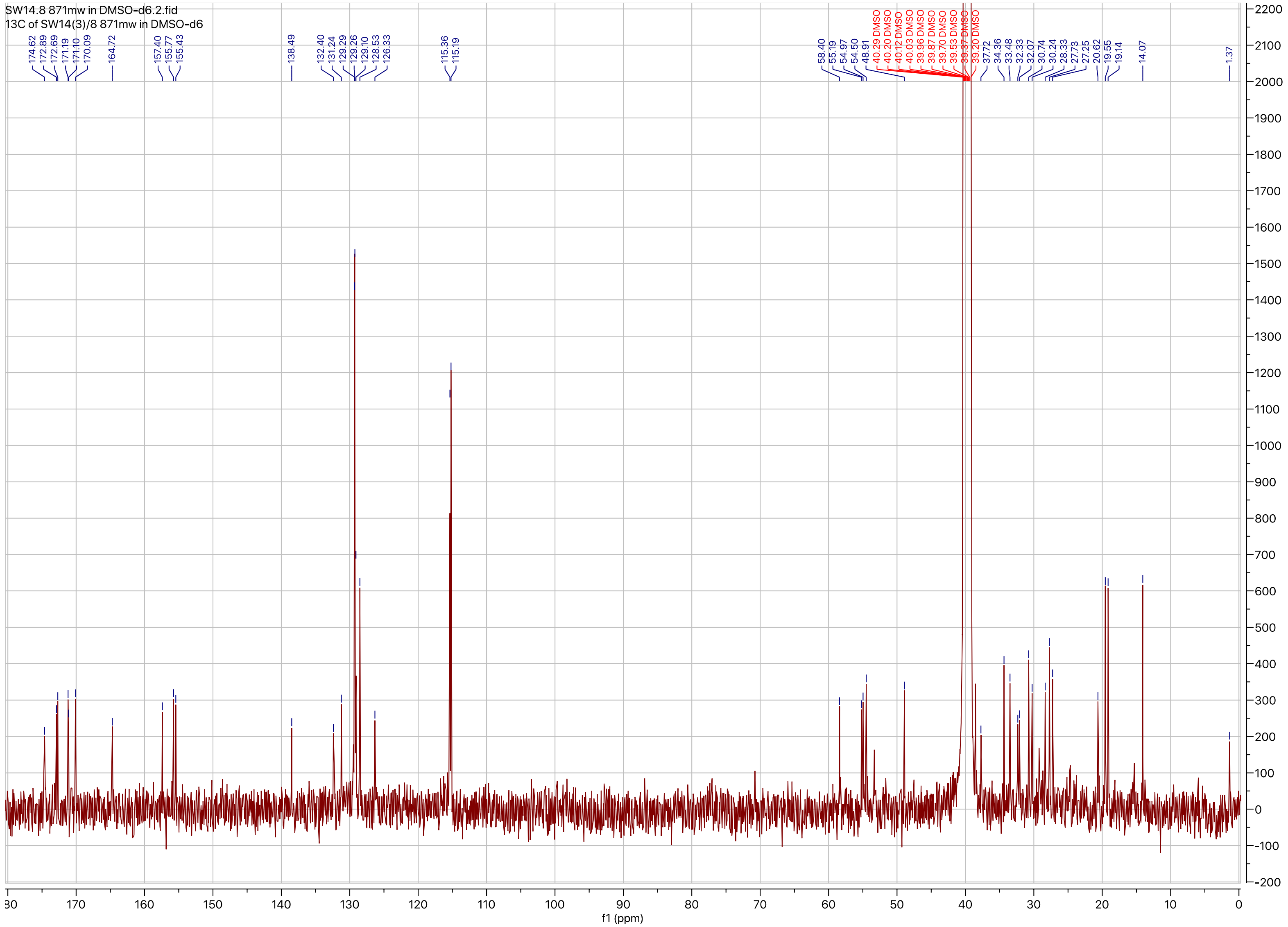

Figure S51. HSQC Spectrum of [1][Dht]-Anabaenopeptin A (**5**) in DMSO-d_6

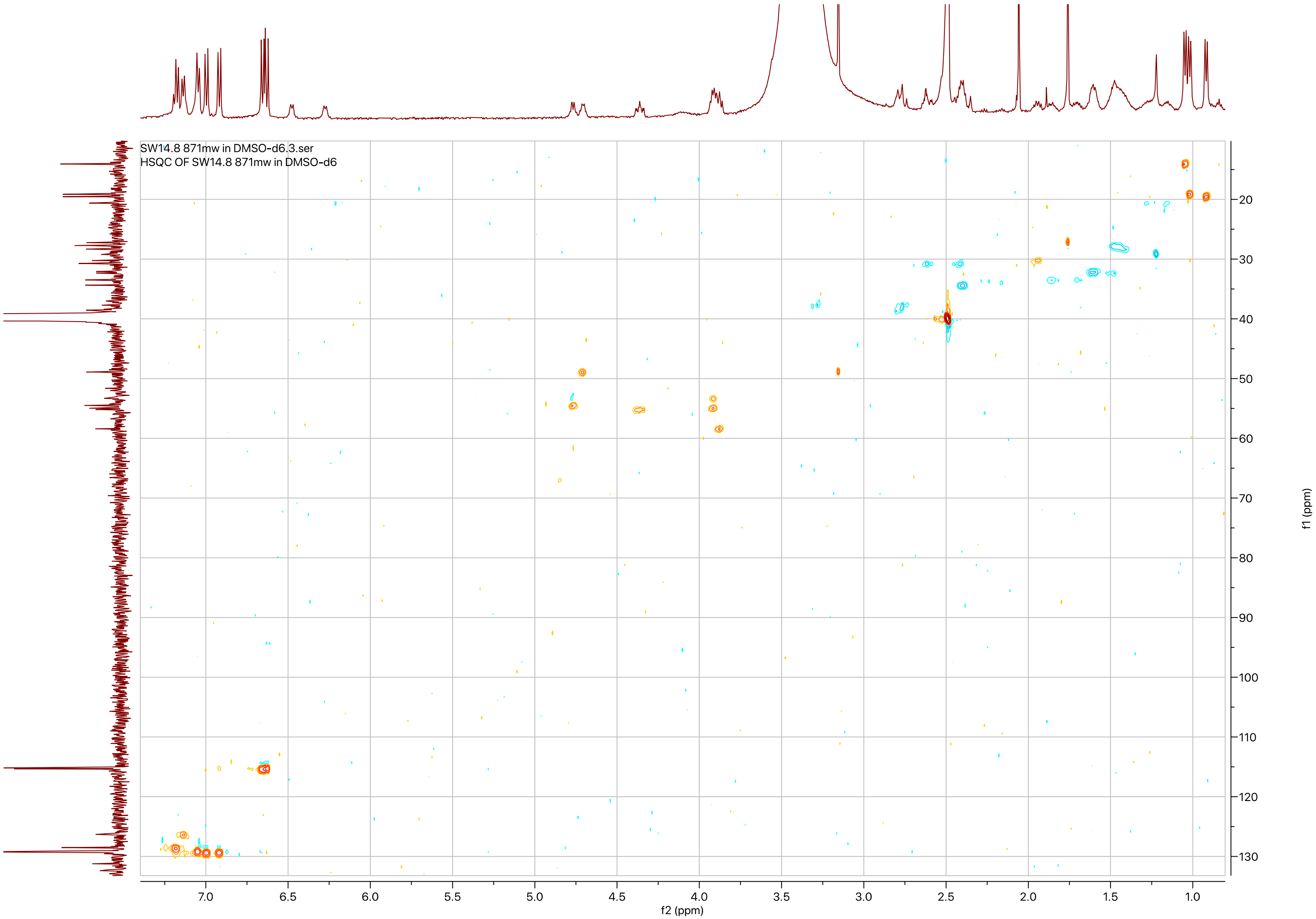

Figure S52. HMBC Spectrum of [1][Dht]-Anabaenopeptin A (**5**) in DMSO-d_6

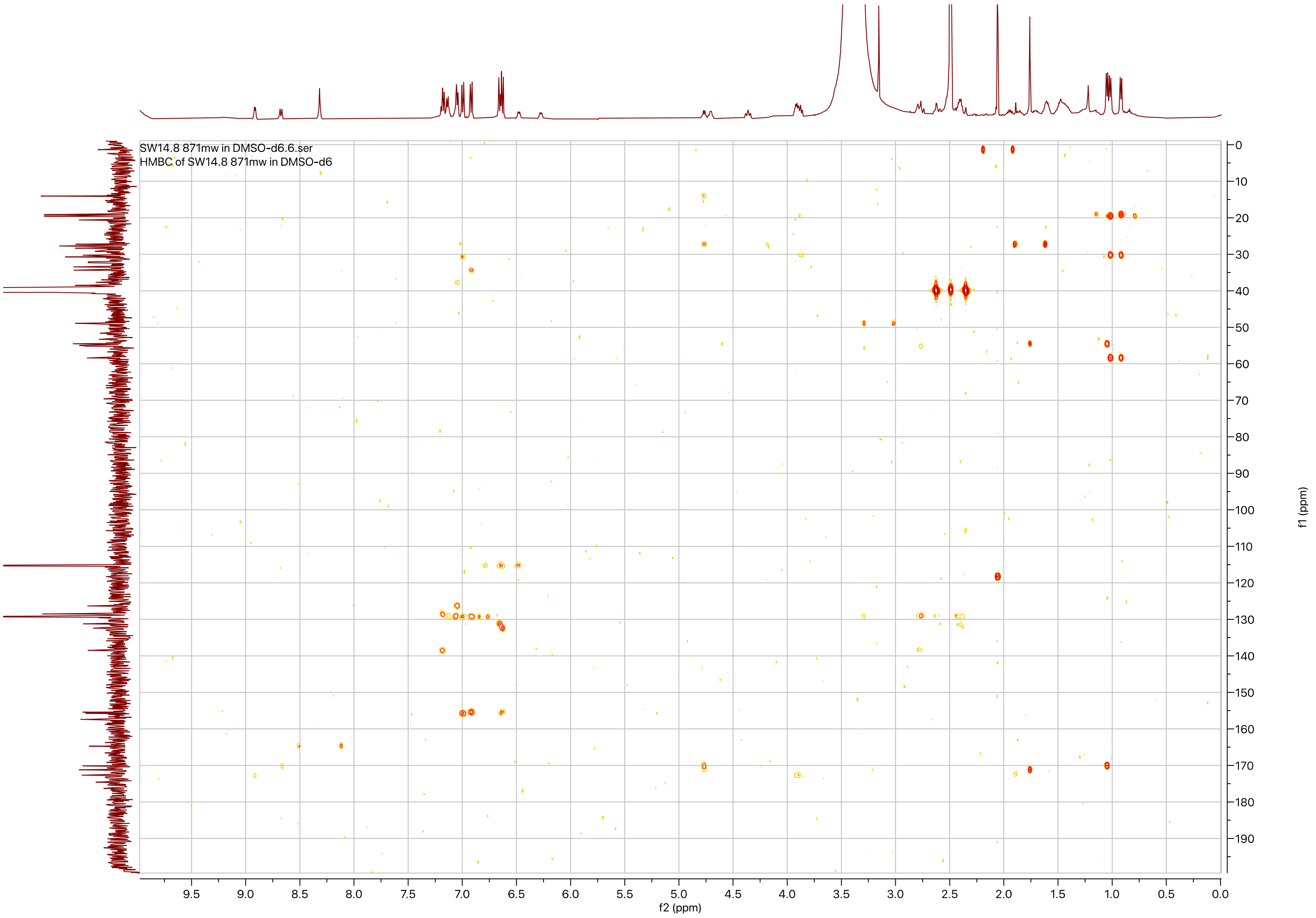

Figure S53. COSY Spectrum of [1][Dht]-Anabaenopeptin A (**5**) in DMSO-d_6

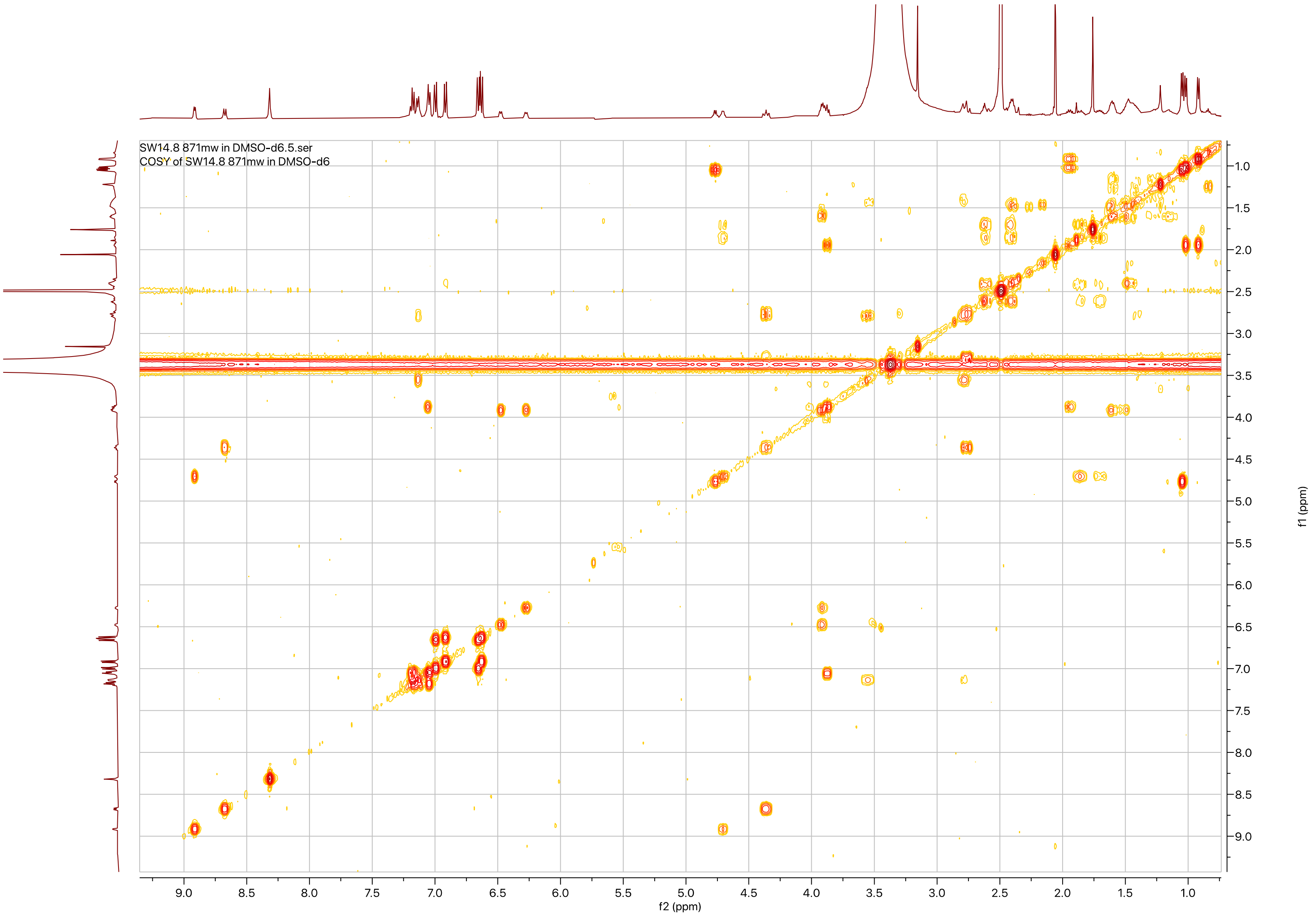

Figure S54. TOCSY Spectrum of [1][Dht]-Anabaenopeptin A (**5**) in DMSO-d_6

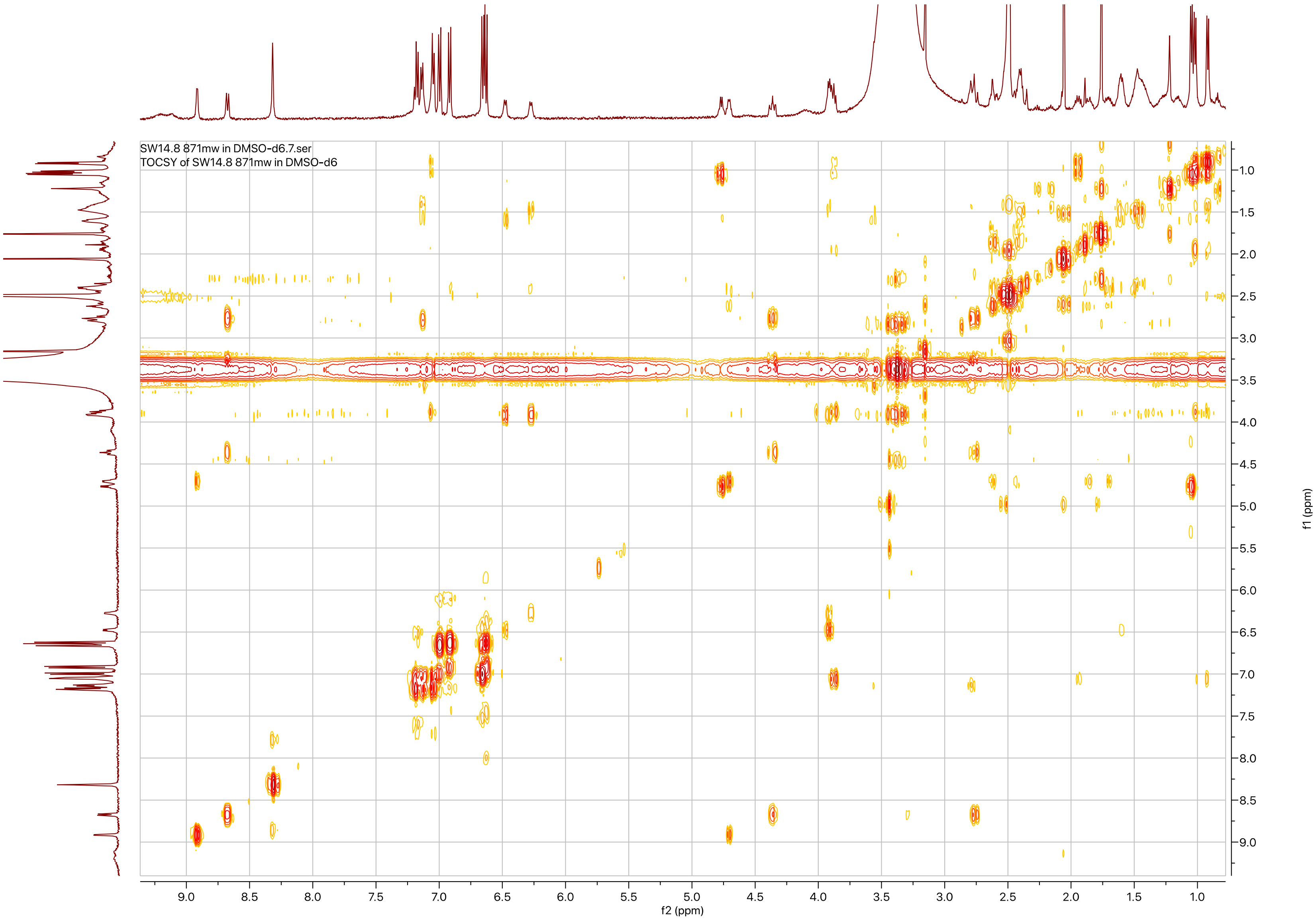

Figure S55. ROESY Spectrum of [1][Dht]-Anabaenopeptin A (**5**) in DMSO-d_6

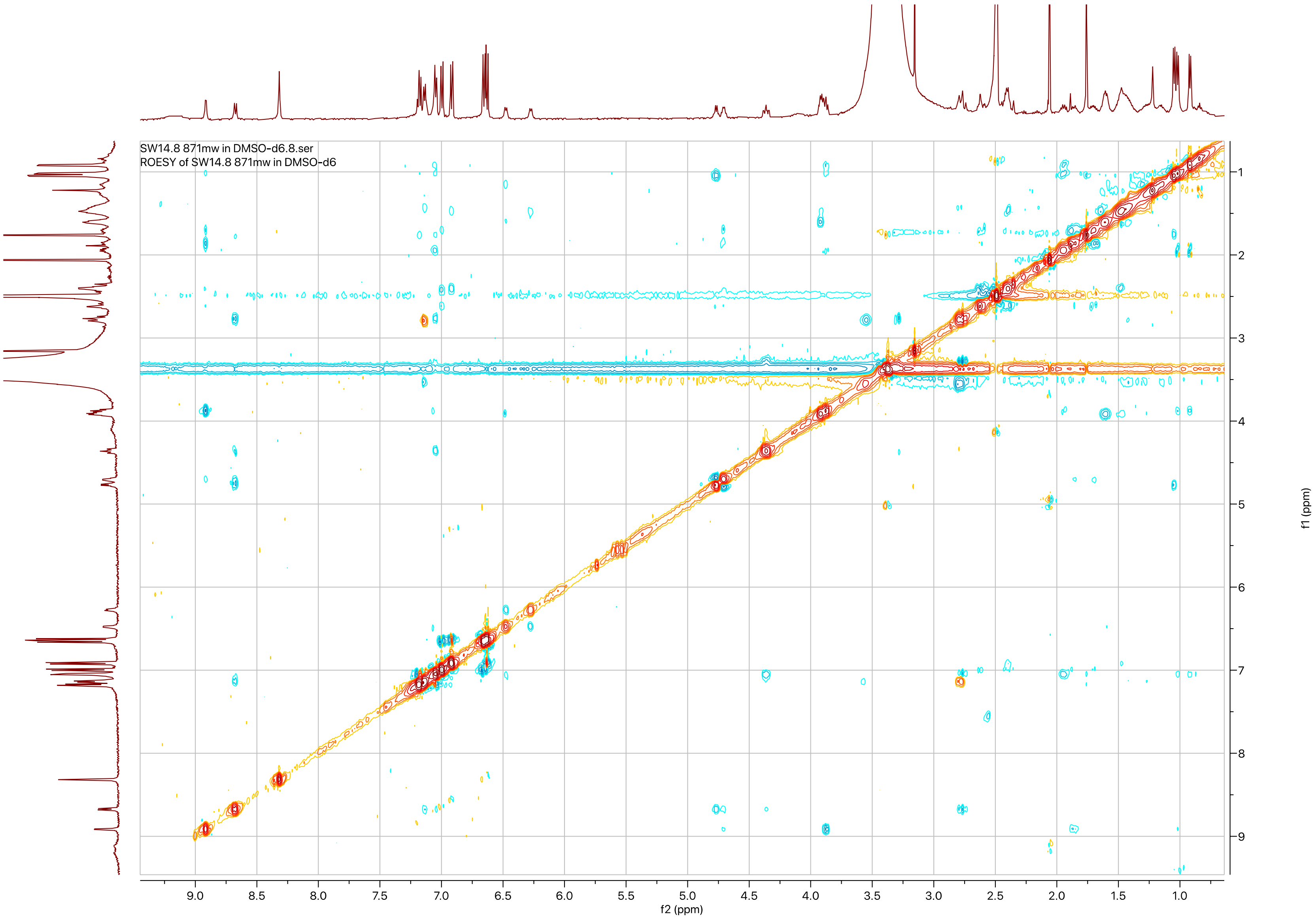

Figure S56. Positive HR ESI MS of [1][Dht]-Anabaenopeptin A (**5**)

Single Mass Analysis
Tolerance = 3.0 PPM / DBE: min = -1.5, max = 100.0
Element prediction: Off
Number of isotope peaks used for i-FIT = 3
Monoisotopic Mass, Odd and Even Electron Ions
462 formula(e) evaluated with 4 results within limits (up to 10 best isotopic matches for each mass)
Elements Used:

Mass	Calc. Mass	mDa	PPM	DBE	Formula	i-FIT	i-FIT Norm	Fit Conf %	C	H	N	O
872.4560	872.4585	-2.5	-2.9	24.0	C49 H60 N8 O7	79.0	0.964	38.16	49	60	8	7
	872.4558	0.2	0.2	19.5	C46 H62 N7 O10	79.3	1.336	26.28	46	62	7	10
	872.4572	-1.2	-1.4	24.5	C47 H58 N11 O6	79.4	1.423	24.09	47	58	11	6
	872.4545	1.5	1.7	20.0	C44 H60 N10 O9	80.2	2.165	11.47	44	60	10	9

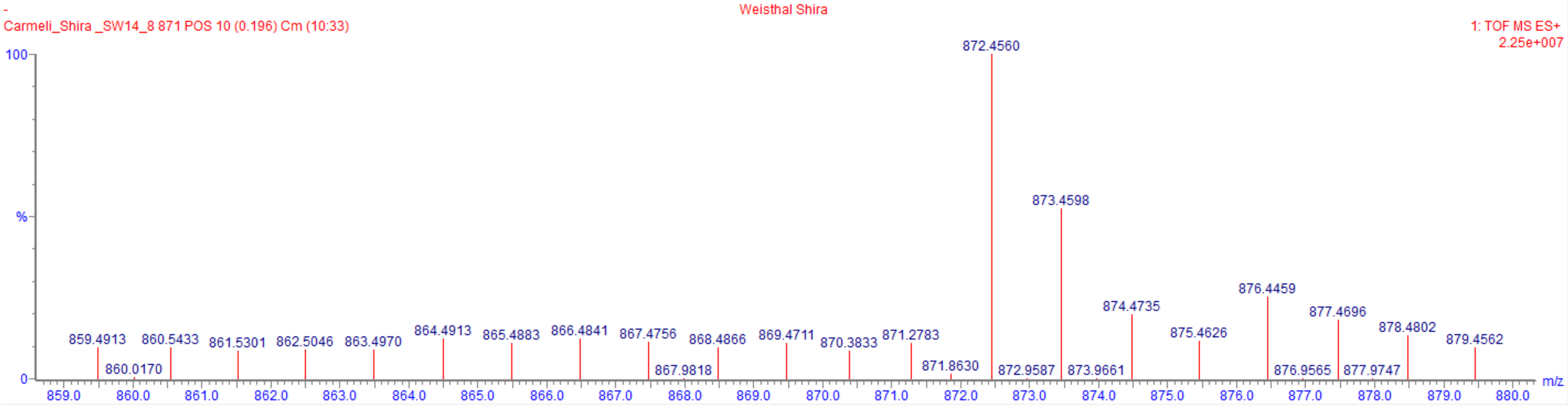

Figure S57. Positive HR ESI MS/MS Spectrum of [1][Dht]-Anabaenopeptin A (**5**)

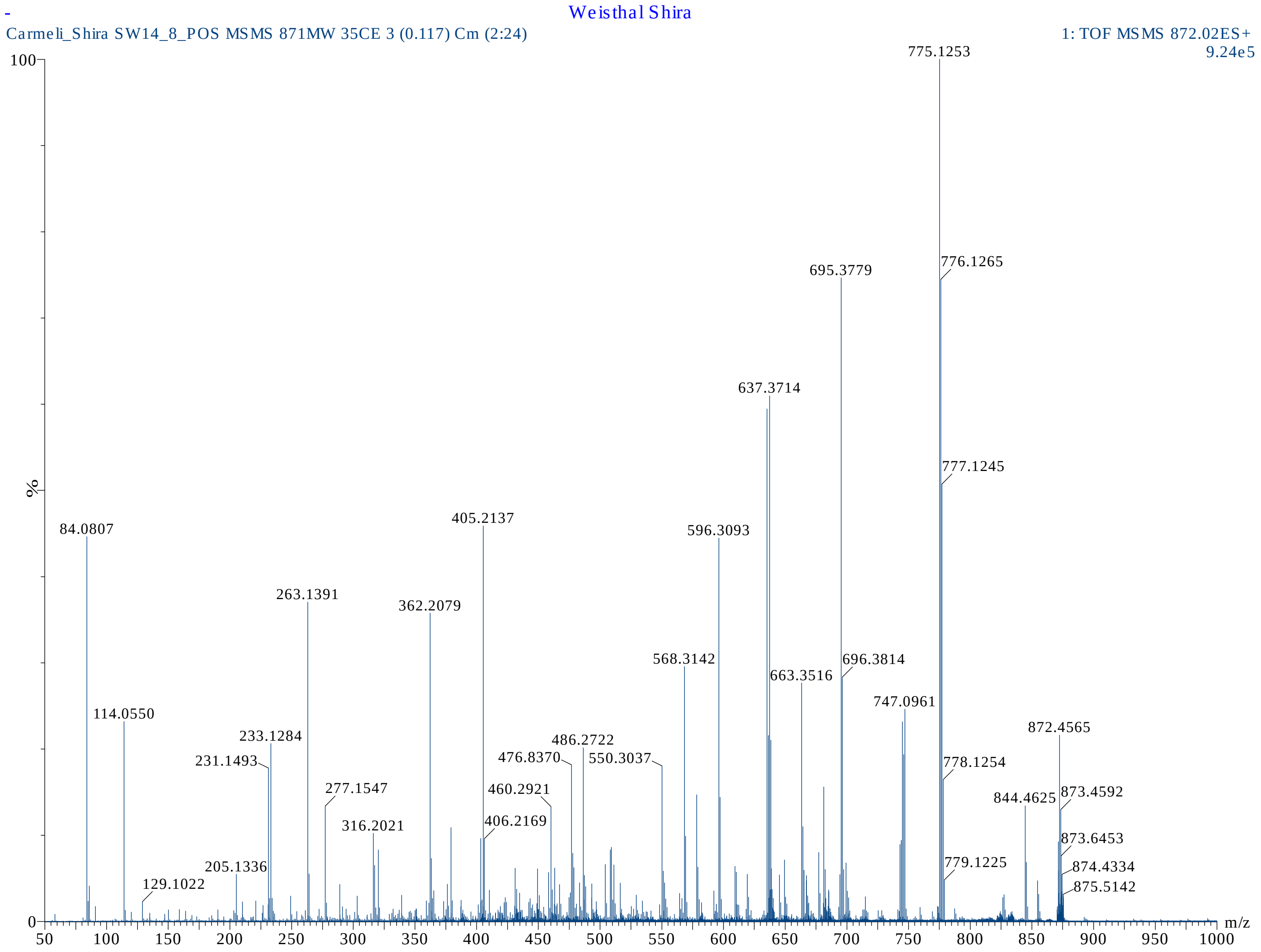

Figure S58. Scheme of Fragmentation in the Positive HR ESI MS/MS Spectrum of [1][Dht]-Anabaenopeptin A (**5**)

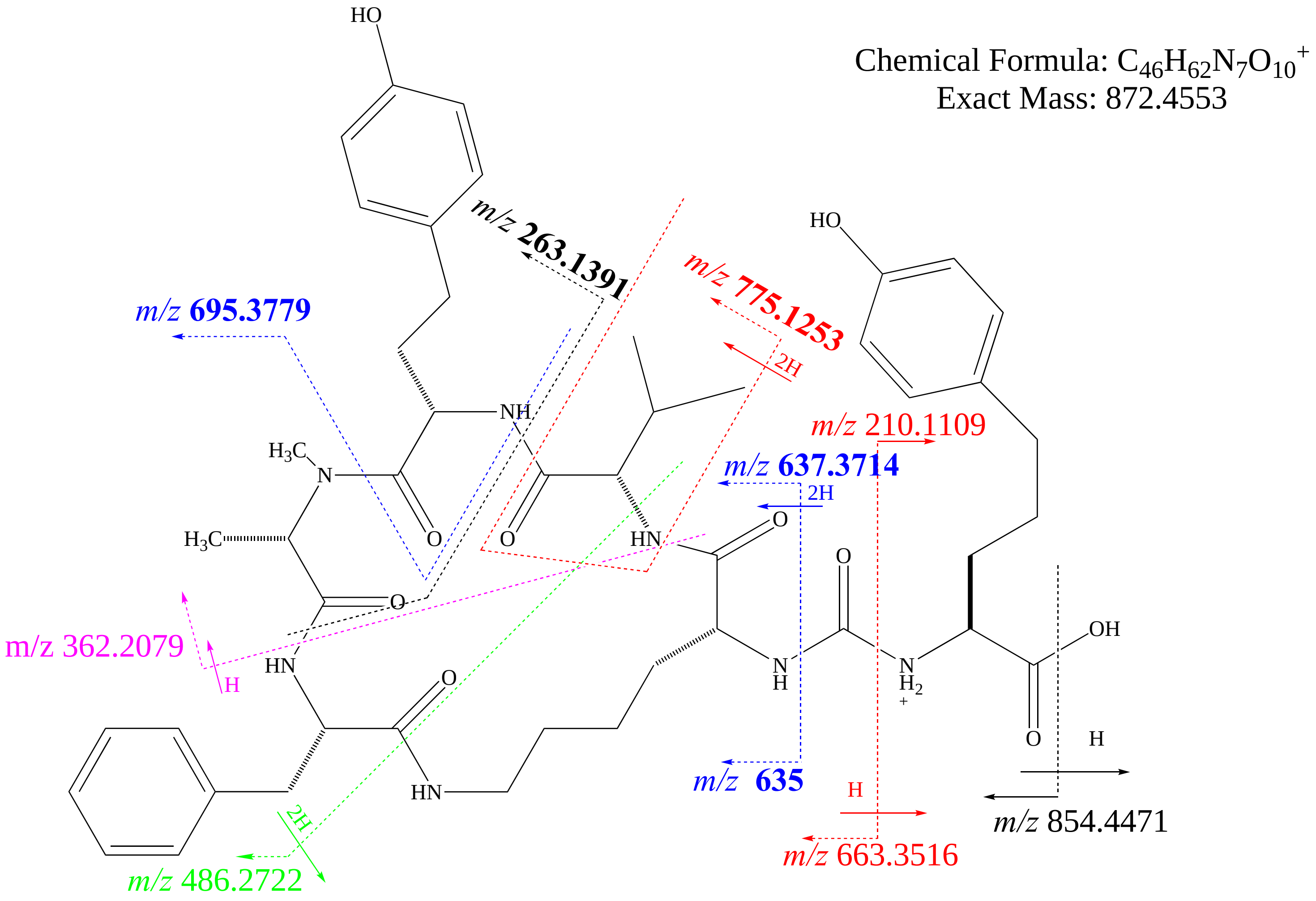

Figure S59. LCMS Traces of Marfey's Method Amino Acids Analysis of [1][Dht]-Anabaenopeptin A (**5**)

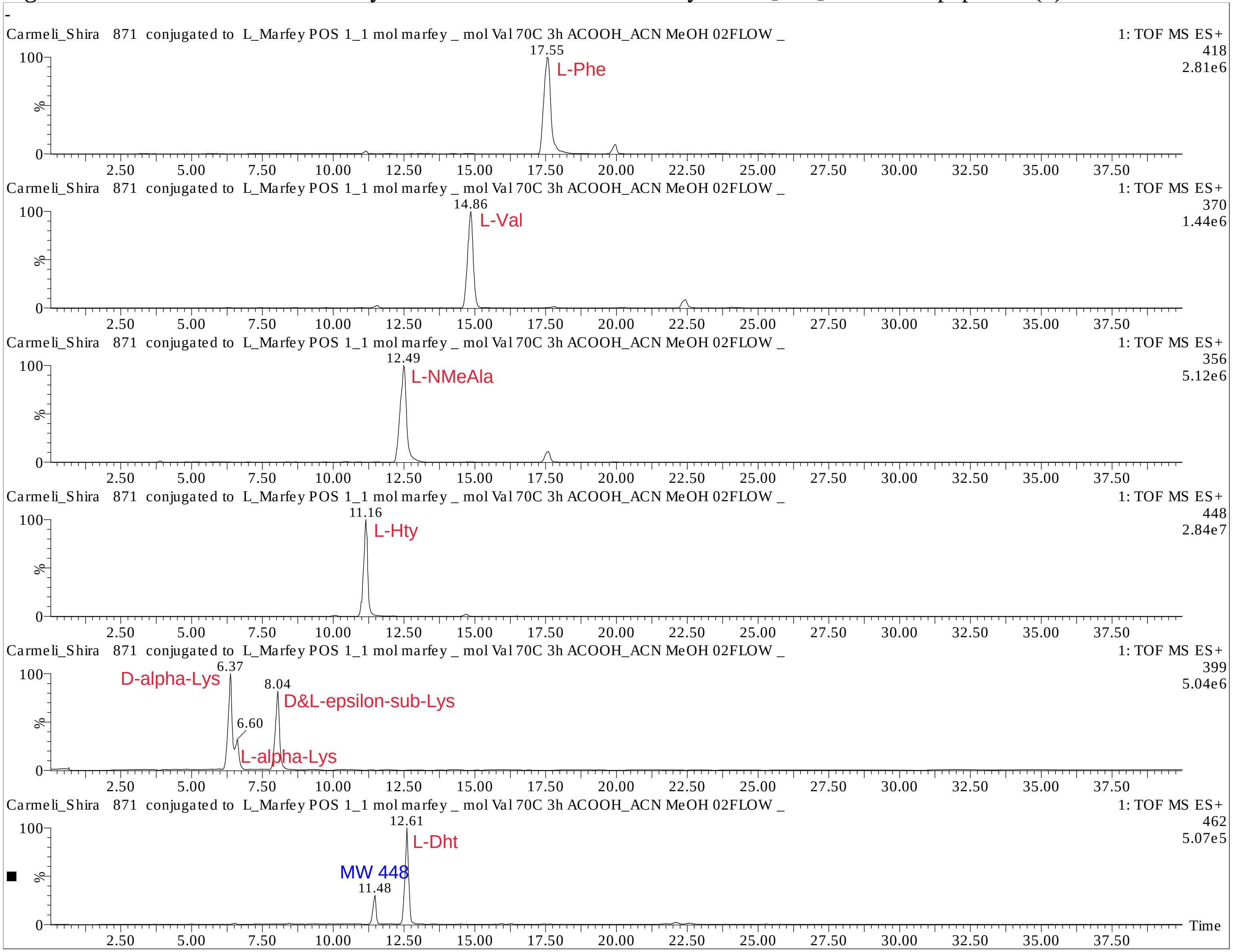

Table S5. NMR Data of [1][Dht]-Anabaenopeptin A (**5**) in DMSO-d_6

Position	δ_C, Mult.	δ_H, Mult. (J in Hz)[b]	HMBC correlations[c]	COSY correlations	TOCSY correlations	ROESY correlations
Dht-1	174.6, C					
Dht-2	53.3, CH	3.91, m	Dht-3a	Dht-2-NH, 3a, 3b	Dht-2-NH, 3a, 3b, 4a, 4b	
Dht-3a<br><br>Dht-3b	32.3, CH_2	1.61, m<br><br>1.50, m		Dht-2, 3b, 4a, 4b<br>Dht-2, 3a, 4a, 4b	Dht-2, 2-NH, 3b, 4a, 4b<br>Dht-2, 2-NH, 3a, 4a, 4b	
Dht-2-NH		6.27, brd (7.0)		Dht-2	Dht-2, 3a, 3b, 4a, 4b	Lys-2-NH, Dht-4a
Dht-4a<br><br>Dht-4b	27.7, CH_2	1.48, m<br><br>1.45, m	Dht-5	Dht-3a, 3b, 5<br>Dht-3a, 3b, 5	Dht-2, 2-NH, 5<br>Dht-2, 2-NH, 5	Dht-2-NH 7,7’
Dht-5	34.4, CH_2	2.40, m	Dht-4b, 7,7’	Dht-4a, 4b		Dht-7,7’
Dht-6	132.4, C		Dht-5, 8,8’			
Dht-7,7’	129.2, CH x2	6.92, d (8.5)	Dht-5, 7’,7,	Dht-8,8’	Dht-8,8’	Dht-4a, 5, 8,8’
Dht-8,8’	115.2, CH x2	6.63, d (8.5)	Dht-8’,8	Dht-7,7’	Dht-7,7’	Dht-7,7’
Dht-9	155.4, C		Dht-7,7’, 8,8’			
Dht-9-OH*		9.20, brs				
CO	157.4, C					
Lys-1	172.7, C		Lys-2			
Lys-2	55.0, CH	3.91, ddd (5.4, 6.3, 7.4)		Lys-3, 6-NH	Lys-3, 6-NH	
Lys-2-NH		6.48, brd (6.9)		Lys-2	Lys-2, 3	Dht-2-NH, Lys-2, 3, 4b
Lys-3	32.1, CH_2	1.60, m	Lys-2	Lys-2, 4a, 4b	Lys-2, 2-NH	Lys-2, 2-NH

Lys-4a<br><br>Lys-4b	20.6, CH_3	1.28, m<br>1.16, m	Lys-2	Lys-3, 5<br><br>Lys-3, 5		Lys-2, 2-NH, 6-NH<br>Lys-2, 2-NH
Lys-5	28.3, CH_2	1.41, m		Lys-4a, 4b, 6a, 6b	Lys-6-NH	Lys-2, 6-NH
Lys-6a<br><br>Lys-6b	38.5, CH_2	3.56, m<br><br>2.78, m		Lys-6b, 6-NH, 5<br>Lys-6a, 6-NH, 5	Lys-6b, 6-NH<br>Lys-6a, 6-NH, 5	Lys-6-NH<br><br>Lys-6-NH
Lys-6-NH		7.14, m		Lys-6a, 6b	Lys-5, 6a, 6b	Phe-2-NH, Lys-4a, 5, 6a, 6b(-)
Val-1	172.9, C		Hty-2-NH			
Val-2	58.4, CH	3.88, dd (7.4, 8.5)	Val-3, 4, 5	Val-2-NH	Val-2-NH, 3, 4, 5	Hty-2-NH, Val-3, 4, 5
Val-2-NH		7.06, m		Val-2	Val-2, 3, 4, 5	
Val-3	30.2, CH_2	1.95, dqq (8.5, 6.3, 6.7)	Val-2, 4, 5	Val-2, 4, 5	Val-2, 2-NH, 4, 5	Phe-5,5’, Val-2,
Val-4	19.6, CH_3	0.92, d (6.3)	Val-2, 5	Val-3	Val-2, 2-NH, 5	Val-2,
Val-5	19.1, CH_3	1.02, d (6.7)	Val-2, 4	Val-3	Val-2, 2-NH, 4,	Val-2,
Hty-1	171.1, C		NMe-Ala-2, NMe			
Hty-2	48.9, CH	4.71, dt (6.7, 6.3)		Hty-2-NH Hty-3a, 3b	Hty-2-NH Hty-3a, 3b, 4a, 4b	Hty-2-NH, 3a, 3b, NMe-Ala-2
Hty-2-NH		8.92, d (4.6)		Hty-2	Hty-2	Hty-2, 3a, 3b, 4a, 4b Val-2
Hty-3a<br><br>Hty-3a	33.5, CH_2	1.85, m<br><br>1.70, m	Hty-4b	Hty-2, 4a, 4b<br>Hty-2, 4a, 4b	Hty-2, 3b, 4a, 4b<br>Hty-2, 3a, 4a, 4b	Hty-2, 2-NH<br>Hty-2, 2-NH, 6,6’
Hty-4a<br><br>Hty-4b	30.7, CH_2	2.62, m<br><br>2.41, m	Hty-6,6’	Hty-3a, 3b, 4b<br>Hty-3a, 3b, 4a	Hty-2, 3a, 3b, 4b<br>Hty-2, 3a, 3b, 4a	Hty-2-NH, 6,6’<br>Hty-2-NH, 6,6’
Hty-5	131.2, C		Hty-4a, 4b, 5,5’			

Hty-6,6’	129.3, CH x2	7.00, d (8.5)	Hty-4a, 4b, 6’,6	Hty-7,7’	Hty-7,7’	Hty-3b, 4a, 4b, 7,7’
Hty-7,7’	115.4, CH x2	6.65, d (8.5)	Hty-7’,7	Hty-6,6’	Hty-6,6’	Hty-6,6’
Hty-8	155.8, C		Hty-6,6’, 7,7’			
Hty-8-OH*		9.12, brs				
NMe-Ala-1	170.1, C		Phe-2-NH, NMe-Ala-2, 3			
NMe-Ala-2	54.5, CH	4.77, q (6.7)	NMe-Ala-3, NMe	NMe-Ala-3	NMe-Ala-3	Phe-2-NH, Hty-2, NMe-Ala-3
NMe-Ala-3	14.1, CH_3	1.05, d (6.7)	NMe-Ala-2	NMe-Ala-2	NMe-Ala-2	NMe-Ala-2
NMe-Ala-NMe	27.3, CH_3	1.76, s	NMe-Ala-2			Phe-2-NH, 5,5’, 6,6’
Phe-1	171.2, C					
Phe-2	55.2, CH	4.36, ddd (3.4, 8.9, 12.7)	Phe-3b	Phe-2-NH, 3a, 3b	Phe-2-NH, 3a, 3b	Phe-2-NH, 3a, 3b, 5,5’
Phe-2-NH		8.68, d (9.0)		Phe-2	Phe-2, 3a, 3b	Lys-6-NH, NMe-Ala-2, NMe Phe-2, 3b
Phe-3a<br>Phe-3a	37.7, CH_2	3.30, m<br>2.77, dd (12.7, 13.4)	Phe-5,5’	Phe-2, 3b<br>Phe-2, 3a	Phe-2, 2-NH<br>Phe-2, 2-NH	Phe-2, 3b, 5,5’<br>Phe-2, 2-NH, 3a, 5,5’
Phe-4	138.5, C		Phe-3b, 6,6’			
Phe-5,5’	129.1, CH x2	7.05, d (7.3)	Phe-3a, 3b, 5’,5, 7	Phe-6,6’	Phe-6,6’	Phe-2, 3a, 3b, 6,6’, Val-3, NMe-Ala-NMe
Phe-6,6’	128.5, CH x2	7.18, dd (7.1, 7.3)	Phe-6’,6	Phe-5,5’,7	Phe-5,5’, 7	Phe-5,5’, 7
Phe-7	126.3, CH	7.13, t (7.1)	Phe-5,5’	Phe-6,6’	Phe-6,6’	Phe-6,6’

[a]500 MHz for ^{1}H and 125 MHz for ^{13}C. [b]From HSQC correlations. [c]J_{C-H}=8 Hz. *May interchange.

Figure S60. LCMS Chromatogram and Mass spectrum of [1][Dht]-Anabaenopeptin A (**5**)

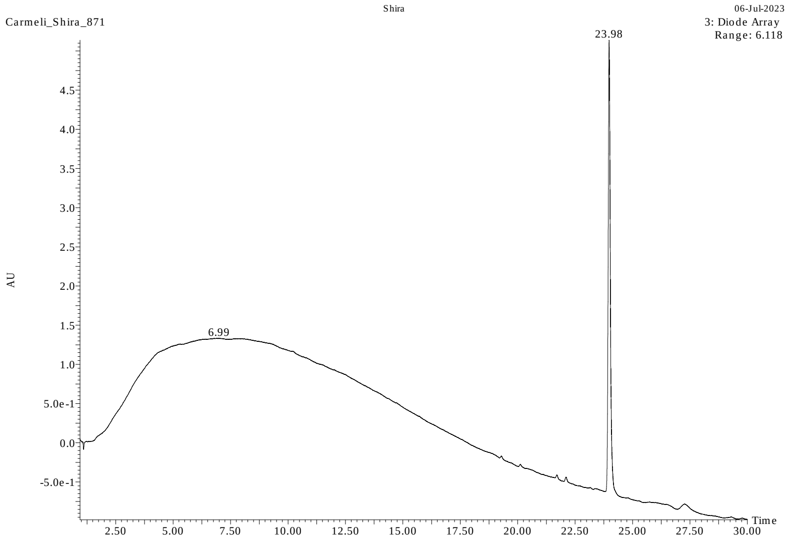

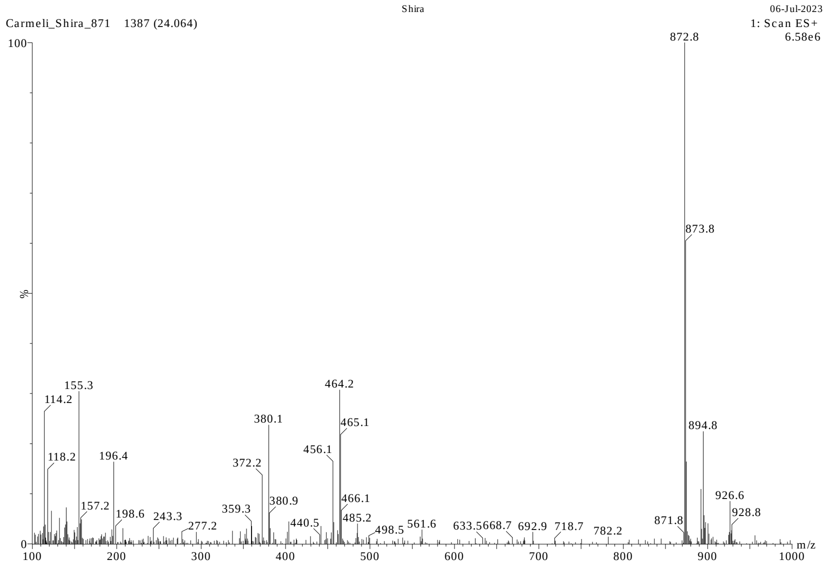

Figure S61. ^{1}H NMR Spectrum of Hydanto-^{1}[Dht]-anabaenopeptin A (**6**) in DMSO-d_6

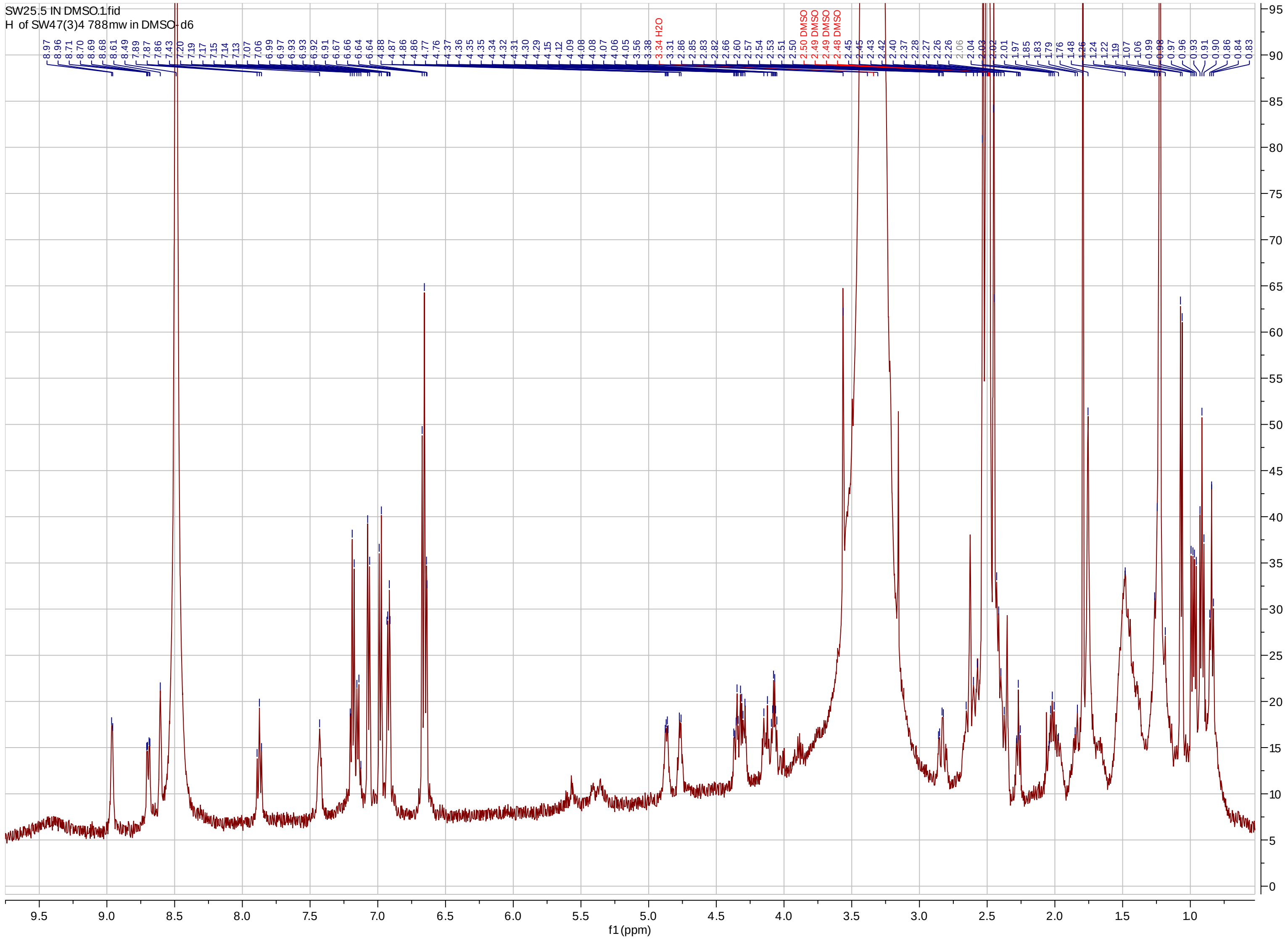

Figure S62. ^{13}C NMR Spectrum of Hydanto-[1][Dht]-anabaenopeptin A (**6**) in DMSO-d_6

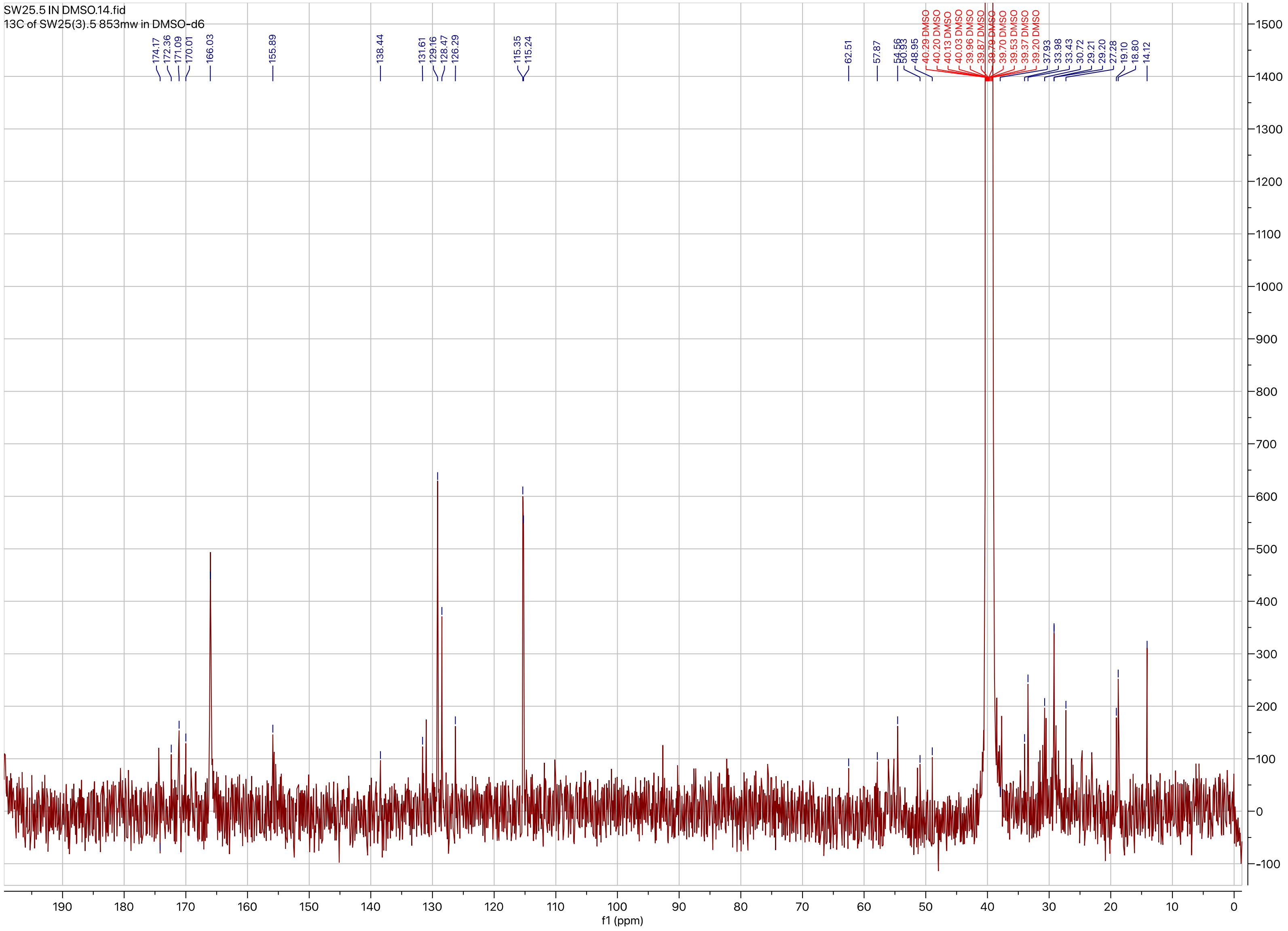

Figure S63. HSQC Spectrum of Hydanto-[1][Dht]-anabaenopeptin A (**6**) in DMSO-d_6

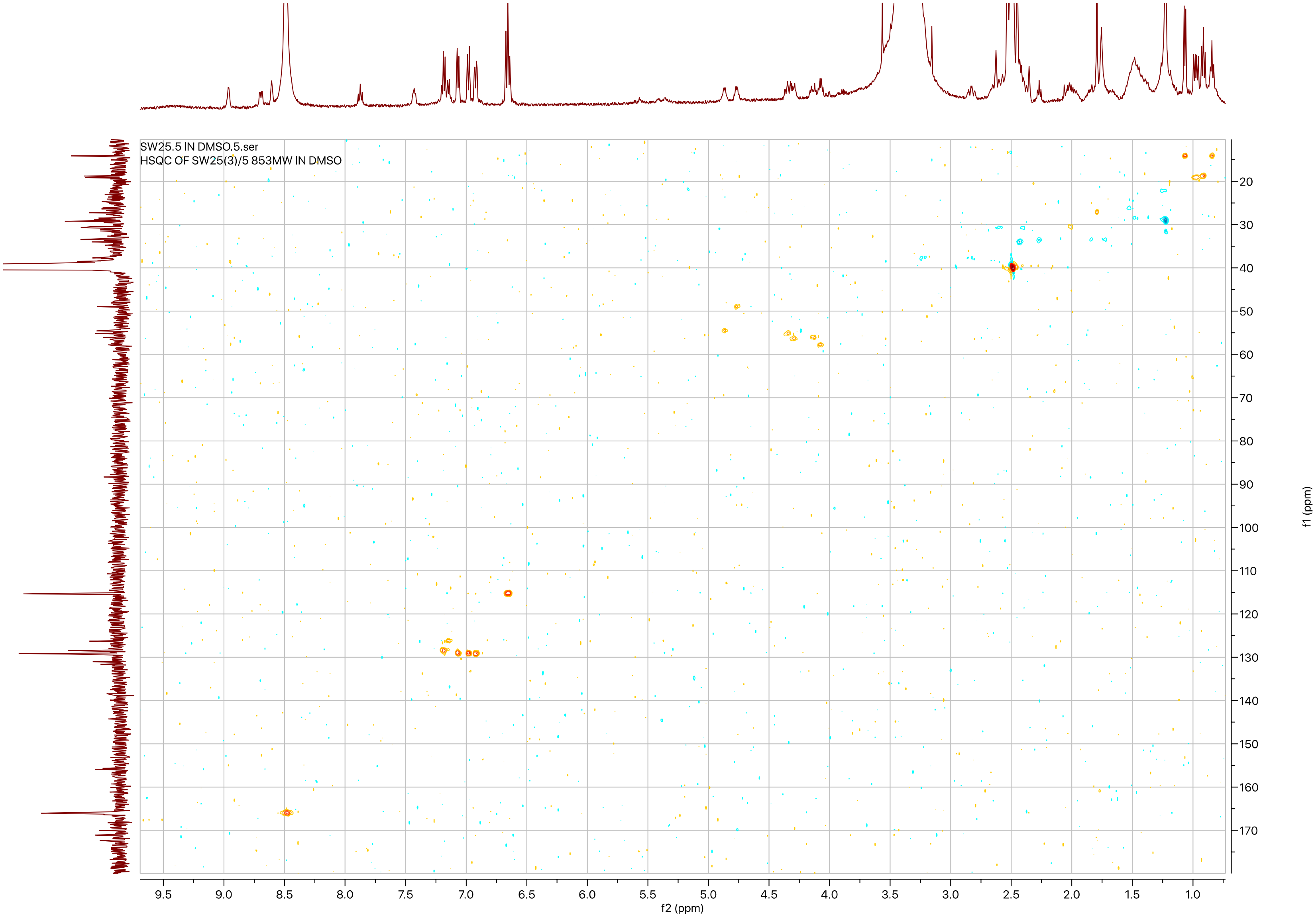

Figure S64. HMBC Spectrum of Hydanto-[1][Dht]-anabaenopeptin A (**6**) in DMSO-d_6

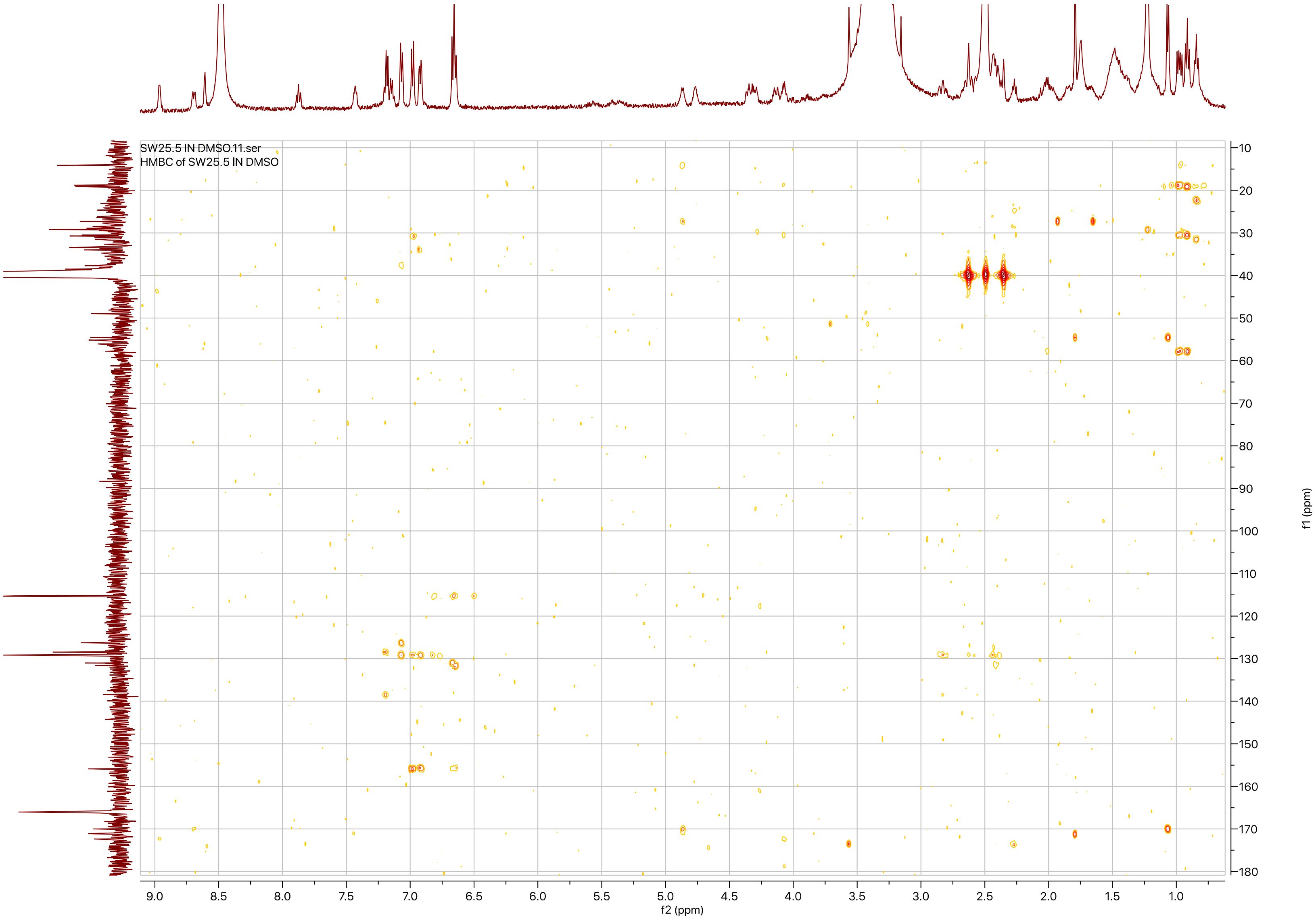

Figure S65. COSY Spectrum of Hydanto-[1][Dht]-anabaenopeptin A (**6**) in DMSO-d_6

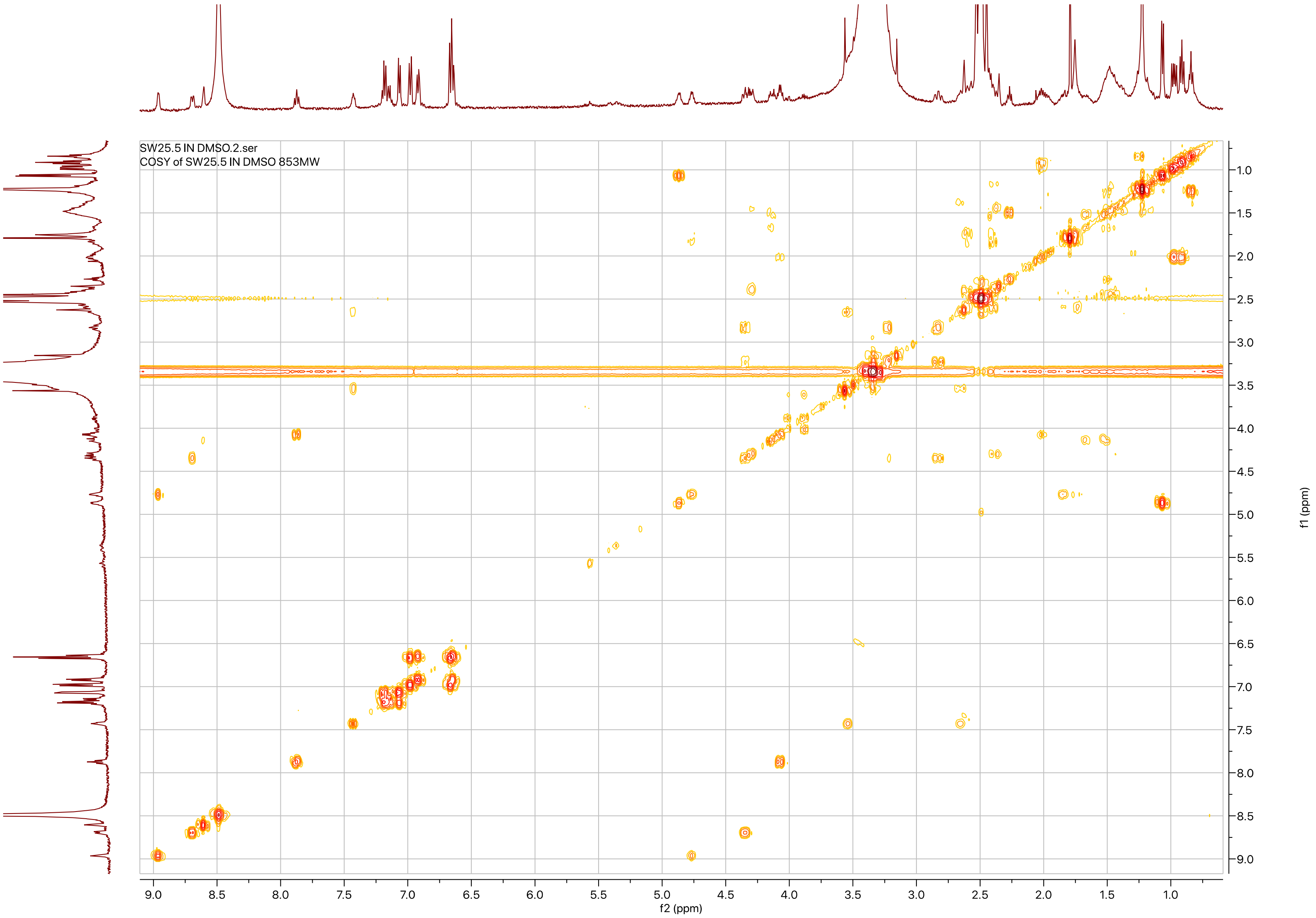

Figure S66. TOCSY Spectrum of Hydanto-[6][Dht]-anabaenopeptin A (**6**) in DMSO-d_6

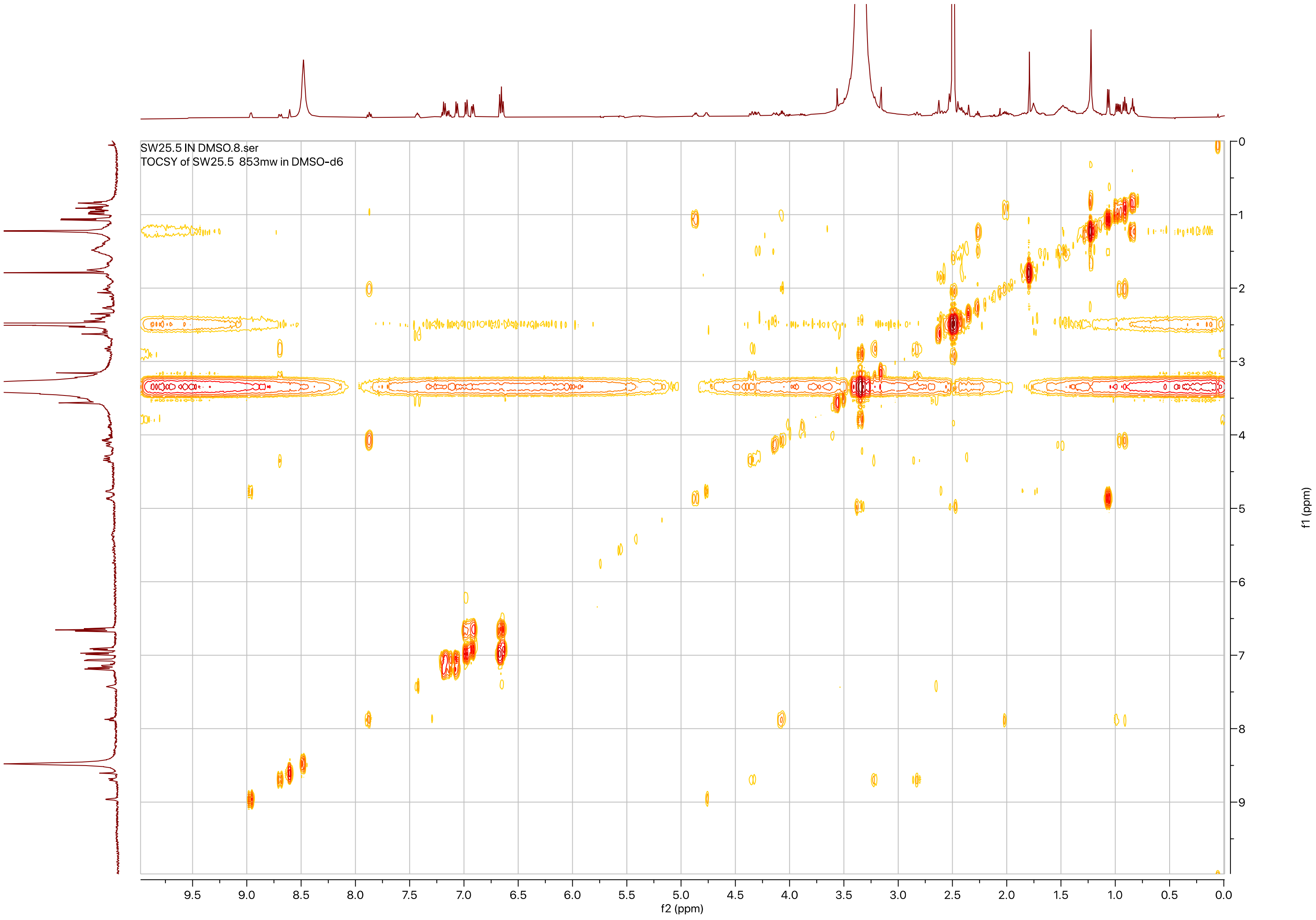

Figure S67. ROESY Spectrum of Hydanto-[1][Dht]-anabaenopeptin A (**6**) in DMSO-d_6

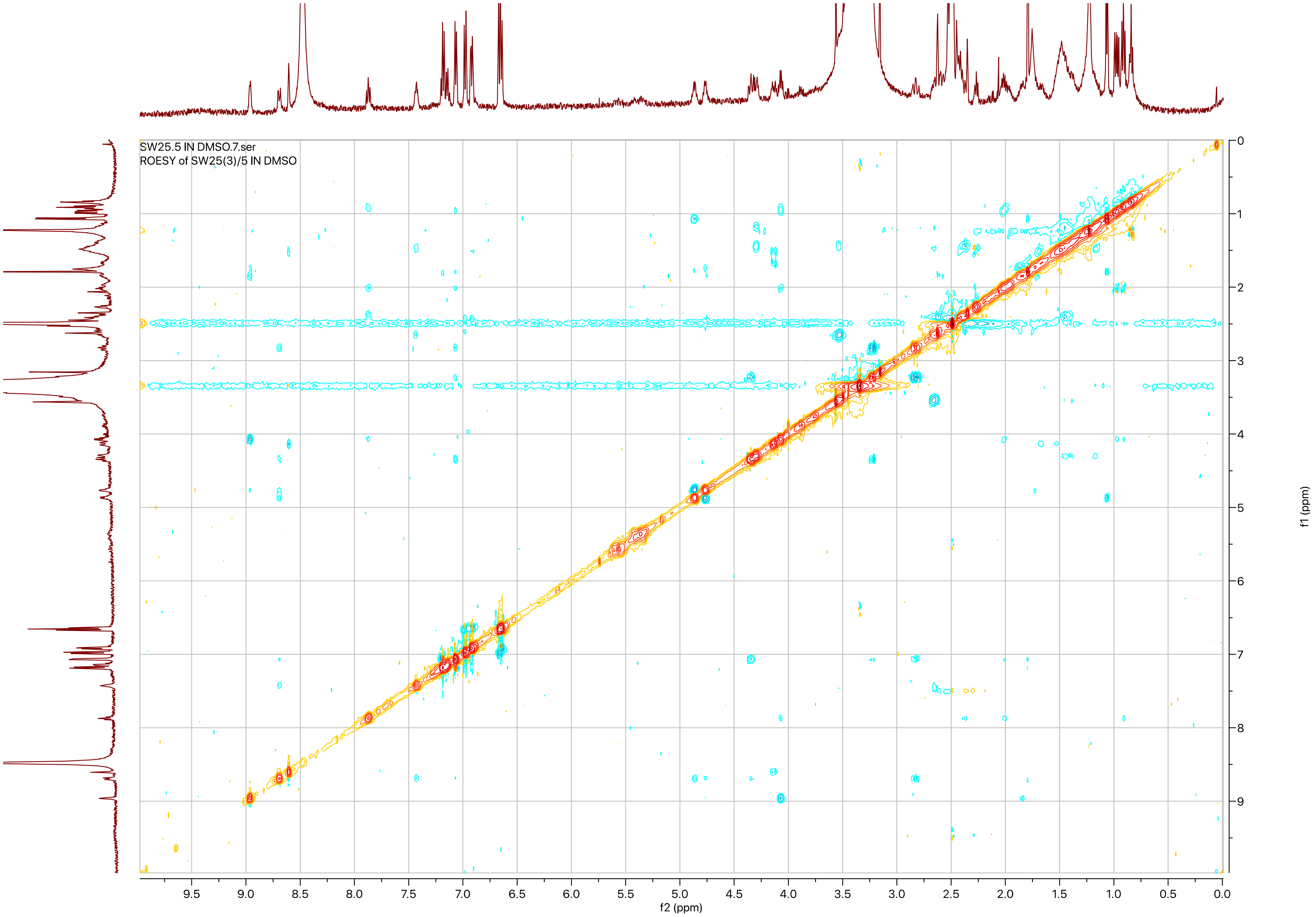

Figure S68. Positive HR ESI MS of Hydanto-[1][Dht]-anabaenopeptin A (**6**)

Single Mass Analysis
Tolerance = 3.0 PPM / DBE: min = -1.5, max = 100.0
Element prediction: Off
Number of isotope peaks used for i-FIT = 3
Monoisotopic Mass, Odd and Even Electron Ions
461 formula(e) evaluated with 4 results within limits (up to 10 best isotopic matches for each mass)
Elements Used:

Mass	Calc. Mass	mDa	PPM	DBE	Formula	i-FIT	i-FIT Norm	Fit Conf %	C	H	N	O
854.4464	854.4439	2.5	2.9	21.0	C44 H58 N10 O8	892.2	0.414	66.10	44	58	10	8
	854.4453	1.1	1.3	20.5	C46 H60 N7 O9	893.1	1.365	25.54	46	60	7	9
	854.4466	-0.2	-0.2	25.5	C47 H56 N11 O5	894.3	2.569	7.66	47	56	11	5
	854.4479	-1.5	-1.8	25.0	C49 H58 N8 O6	896.7	4.948	0.71	49	58	8	6

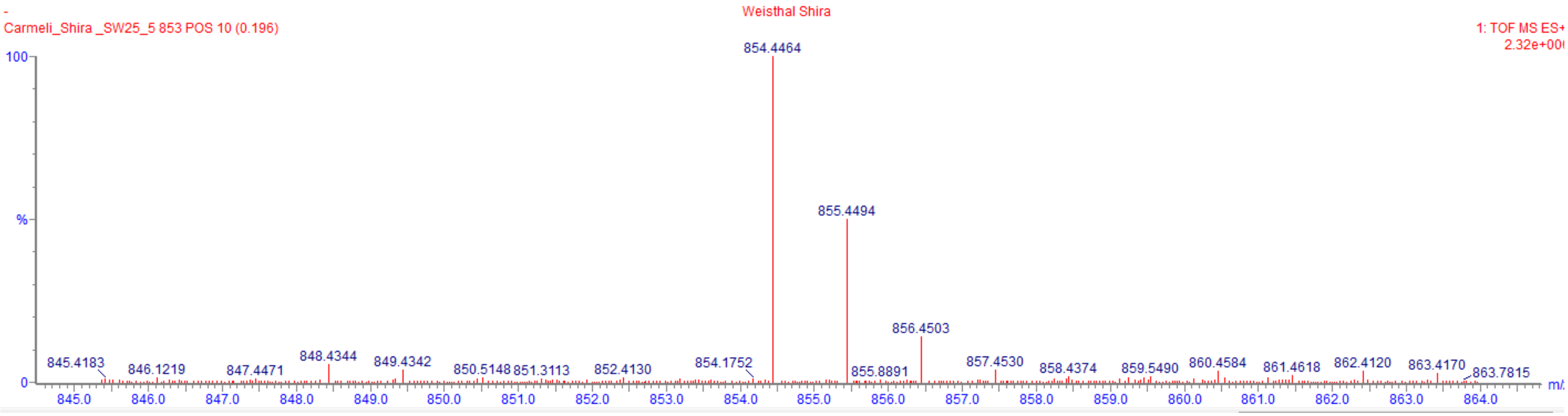

Figure S69. Positive HR ESI MS/MS Spectrum of Hydanto-[1][Dht]-anabaenopeptin (**6**)

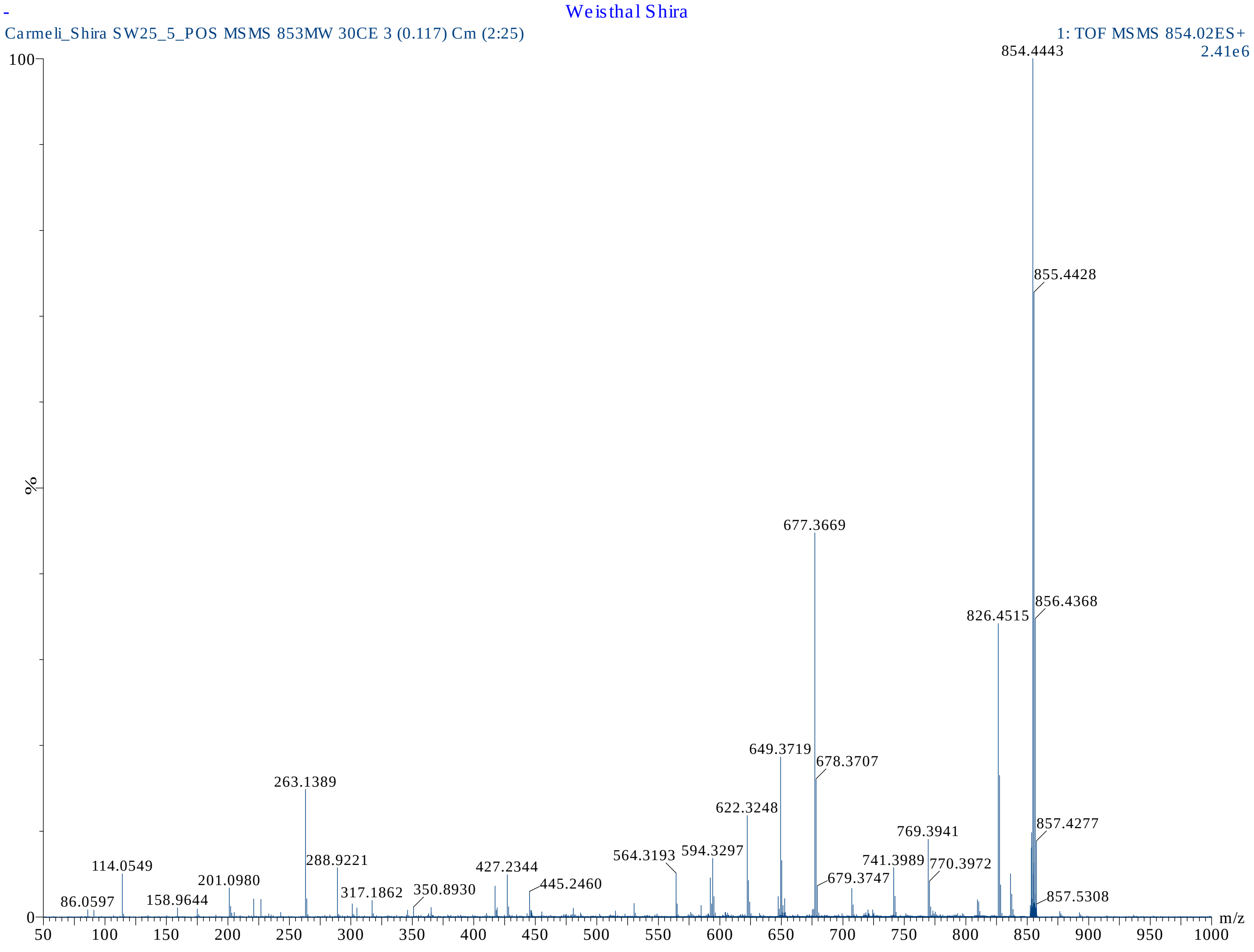

Figure S70. Scheme of Fragmentation in the Positive HR ESI MS/MS Spectrum of Hydanto-[1][Dht]-anabaenopeptin (**6**)

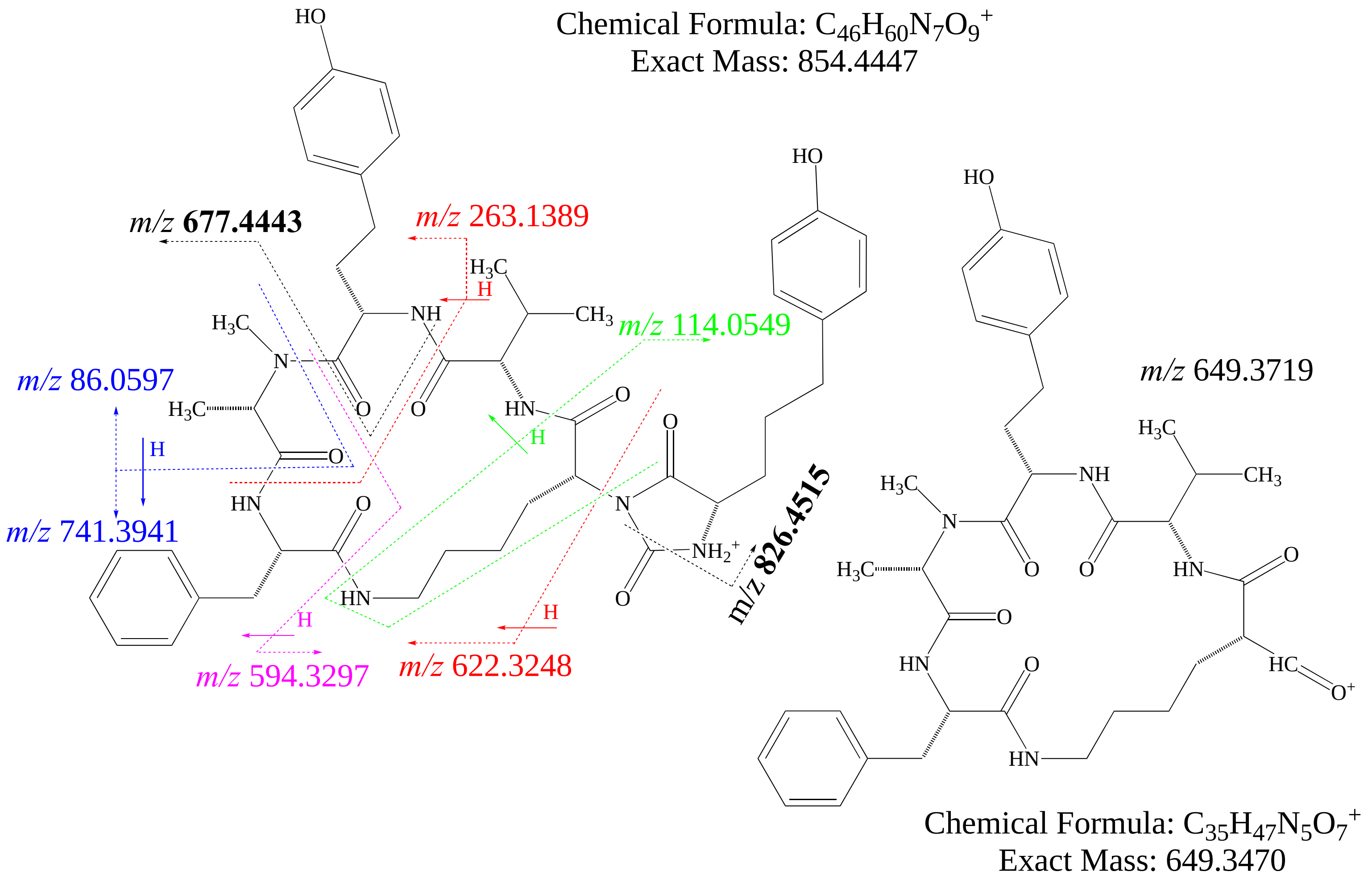

Figure S71. LCMS Traces of Marfey's Method Amino Acids Analysis of Hydanto-[1][Dht]-anabaenopeptin (**6**)

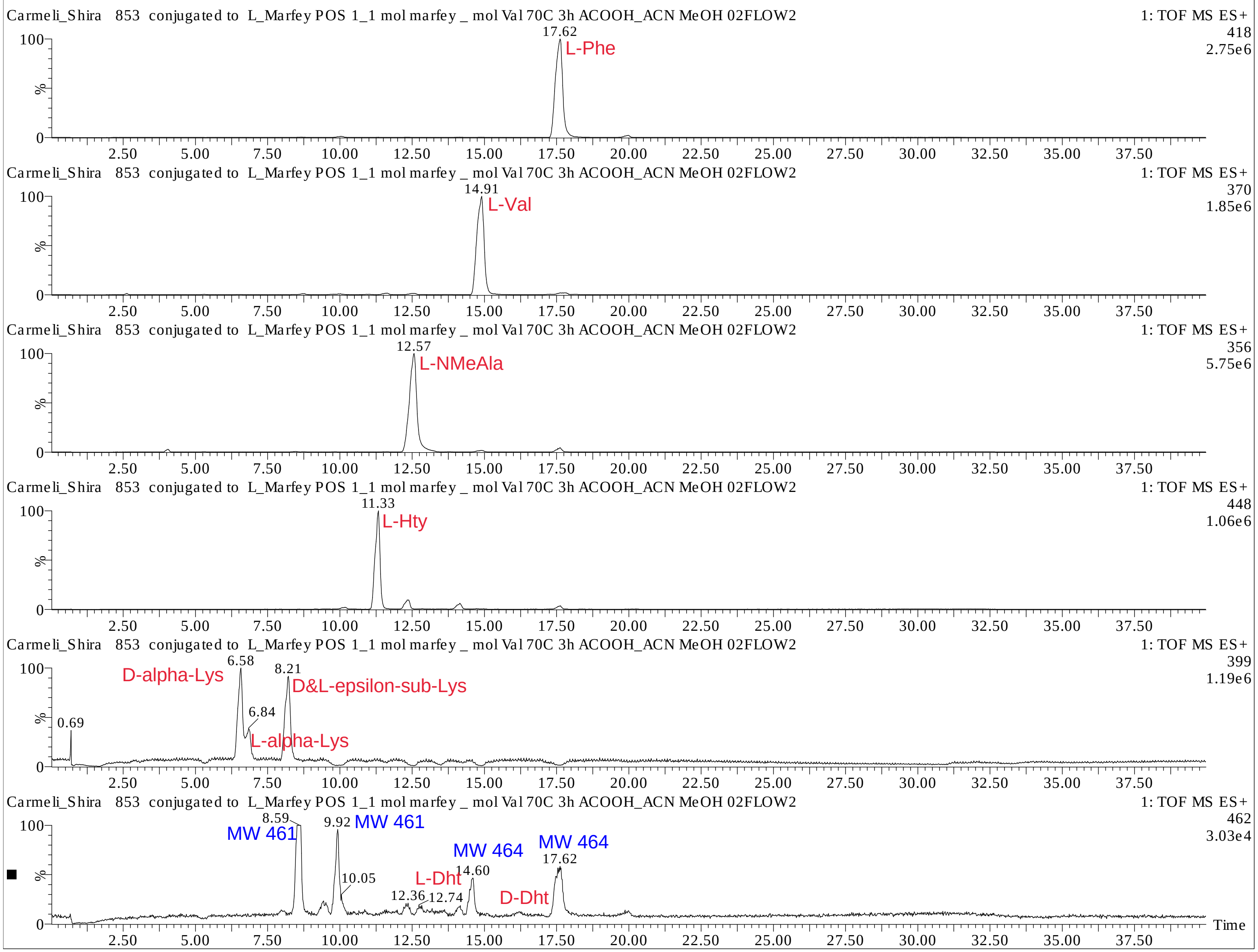

Table S6. NMR Data of Hydanto-[1][Dht]-anabaenopeptin A (**6**) in DMSO-d_6

Position	δ_C, Mult.	δ_H, Mult. (*J* in Hz)[b]	HMBC correlations[c]	COSY correlations	TOCSY correlations	ROESY correlations
Dht-1<br>*Dht-1*	174.0, C<br>*174.1, C*		Dht-2-NH			
Dht-2<br>*Dht-2*	56.2, CH<br>*56.1, CH*	4.15, m<br>4.12, m	Dht-2-NH, 4	Dht-3a,3b	Dht-3b	
Dht-2-NH		8.61, brs				Dht-2
Dht-3a<br><br>Dht-3b	30.8, CH_2	1.66, m<br><br>1.50, m		Dht-2,3b,4a, 4b<br>Dht-2,3a		Dht-2<br><br>Dht-2
Dht-4a<br>Dht-4b<br>*Dht-4*	28.9, CH_2<br><br>*28.7, CH_2*	1.48, m<br>1.45, m		Dht-3a,5<br>Dht-3a,5		
Dht-5	34.0, CH_2	2.42, m	Dht-8,8’	Dht-4a,4b		
Dht-6	131.6, C		Dht-3, 7,7’			
Dht-7,7’<br>*Dht-7,7’*	129.3, CH<br>*129.2, CH*	6.92, d (8.5)<br>*6.92, d (8.5)*	Dht-3,7’,7	Dht-8,8’<br>*Dht-8,8’*		Dht-3, 8,8’<br>*Dht-8,8’*
Dht-8,8’<br>*Dht-8,8’*	115.2, CH x2	6.65, d (8.5)<br>*6.65, d (8.5)*	Dht-8’,8	Dht-7,7’<br>*Dht-7,7’*		Dht-7,7’<br>*Dht-7,7’*
Dht-9	155.9, C		Dht-7,7’,8,8’			
CO<br>*CO*	156.9, C<br>*157.0, C*		Lys-2, Dht-2<br>*Lys-2*			
Lys-1	168.4, C<br>*168.2, C*		Lys-2			
Lys-2<br>*Lys-2*	56.5, CH<br>*56.3, CH*	4.31, m<br>*4.29, m*		Lys-3a,3b	Lys-3a,5b	Lys-4a,4b, 5b
Lys-3a<br>Lys-3b<br>*Lys-3a*	29.6, CH_2,<br><br>*29.5, CH_2*	2.39, m<br>1.44, m		Lys-2,4a, 4b		 Lys-2
Lys-4a<br>Lys-4b	23.1, CH_2	1.45, m<br>1.17, m		Lys-4b Lys-4a		Lys-2<br>Lys-2

Lys-5a<br>Lys-5b	28.6, CH_2<br>*28.5, CH_2*	1.48, m<br>1.38, m		 Lys-6b		
Lys-6a<br>Lys-6b	38.5, CH_2	3.53, m<br>2.65, m		Lys-6b, 6-NH<br>Lys-5b,6a,6-NH	Lys-6-NH<br>Lys-6-NH	2.65, Lys-4a 1.38<br>Lys-6a
Lys-6-NH		7.43, t (6.4)		Lys-6a,6b	Lys-6a,6b	Phe-2,2-NH, Lys-6a,6b
Val-1	172.4, C		Hty-2-NH, Val-2			
Val-2<br>*Val-2*	57.9, CH<br>*57.7, CH*	4.08, t (7.6)<br>*4.07, t (7.6)*	Val-4,5<br>*Val-4,5*	Val-2-NH, 3<br>*Val-2-NH, 3*	Val-2-NH, 3,4,5	Val-3,4,5
Val-2-NH<br>*Val-2-NH*		7.88, d (8.2)<br>*7.86, d (7.9)*		Val-2<br>*Val-2*	Val-2,3,4,5	Val-2,3,5, Lys-3a<br>*Val-2,5*
Val-3	30.5, CH	2.01, m	Val-2-NH,4,5	Val-2,4,5	Val-2-NH	
Val-4<br>*Val-4*	19.2, CH_3<br>*19.1, CH_3*	0.99, d (7.1)<br>*0.96, d (7.5)*	Val-5		Val-2-NH	
Val-5<br>*Val-5*	18.8, CH_3	0.92, d (6.9)<br>*0.90, d (6.7)*	Val-4		Val-2-NH	
Hty-1	171.2, C		NMe-Ala-2,NMe			
Hty-2	49.0, CH	4.77, m		Hty-2-NH,3a, 3b	Hty-2-NH,3a, 3b,4a,	NMe-Ala-2, 1 Hty-3a,3b
Hty-2-NH		8.96, brs		Hty-2	Hty-2	Hty-2,3a, 3b, Val-2
Hty-3a<br>Hty-3b	33.4, CH_2	1.85, m<br>1.72, m		Hty-2,3b,4a, 4b<br>Hty-2,3a,4a,4b		
Hty-4a<br>Hty-4b	30.7, CH_2	2.60, m<br>2.40, m	Hty-2-NH	Hty-3a,3b, 4b<br>Hty-3a,3b, 4a		 Hty-6,6’
Hty-5	131.0, C		Hty-4b,7,7’			
Hty-6,6’	129.2, CH x2	6.98, d x2 (8.5)	Hty-4a,4b			Hty-7,7’
Hty-7,7’	115.4, CH x2	6.66, d x2 (8.5)	Hty-7’,7			Hty-2-NH
Hty-8	155.7, C		Hty-7,7’			

NMe-Ala-1	170.0, C		Phe-2-NH, NMe-Ala-2,3			
NMe-Ala-2	54.6, CH	4.87, q (6.7)	NMe-Ala-3, NMe	NMe-Ala-3	NMe-Ala-3	Phe-2-NH, Lys-6-NH, Hty-2, NMe-Ala-3
NMe-Ala-3	14.1, CH_3	1.06, d (6.7)	NMe-Ala-2	NMe-Ala-2		
NMe-Ala-Me	27.3, CH_3	1.79, s	NMe-Ala-2			Phe-2-NH, Phe-6,6’
Phe-1	171.1, C		Lys-6-NH			
Phe-2<br><br>*Phe-2*	55.2, CH	4.35, ddd (12.2, 8.7, 3.2)<br>*4.31, m*	Phe-3b	Phe-2-NH, 3a,3b<br>*Phe-3b*	Phe-2-NH,3a, 3b	Phe-3a
Phe-2-NH<br><br>*Phe-2-NH*		8.69, d (8.3)<br><br>*8.69, d (9.0)*		Phe-2<br><br>*Phe-2*	Phe-2,3a,3b	Lys-6-NH, Phe-2,3b,5, 5’, Hty-2, NMe-Ala-2,NMe
Phe-3a<br>Phe-3b<br><br>*Phe-3b*	37.7, CH_2	3.23, m<br>2.81, dd (13.3,3.9)<br>*2.84, dd (13.3,3.9)*	Phe-5,5’	Phe-2,3b<br>Phe-2,3a<br><br>*Phe-2*		Phe-2,3a, 3b
Phe-4	138.4, C		Phe-3b,6,6’			
Phe-5,5’	129.1, CH	7.07, d x2 (7.4)	Phe-3b,5’,5			Phe-2, 2-NH,3a,3b, Val-3,4,5, NMe-Ala-Me,
Phe-6,6’	128.5, CH	7.19, t x2 (7.4)	Phe-6’,6			NMe-Ala-Me
Phe-7	126.3, CH	7.14, t (7.4)	Phe-5,5’			

[a]500 MHz for ^{1}H and 125 MHz for ^{13}C. [b]From HSQC correlations. [c]$J_{C\text{-}H}$=8 Hz.

Figure S72. LCMS Chromatogram and Mass spectrum of Hydanto-[1][Dht]-anabaenopeptin A (**6**)

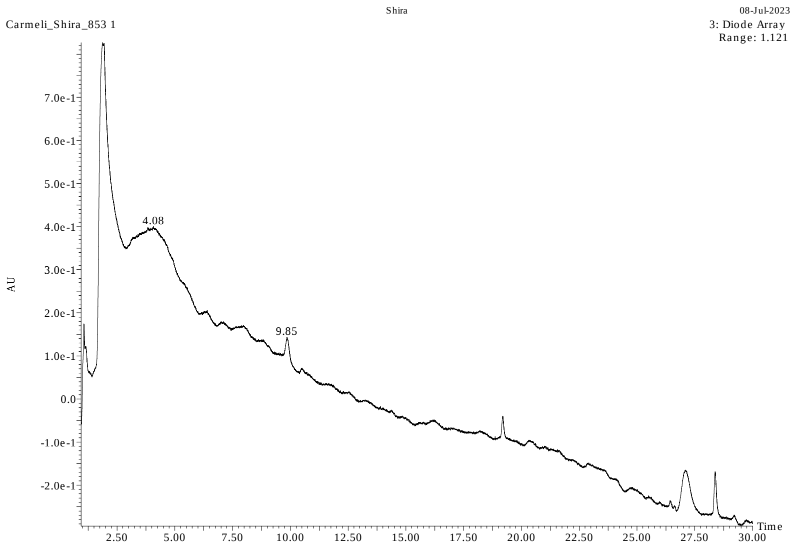

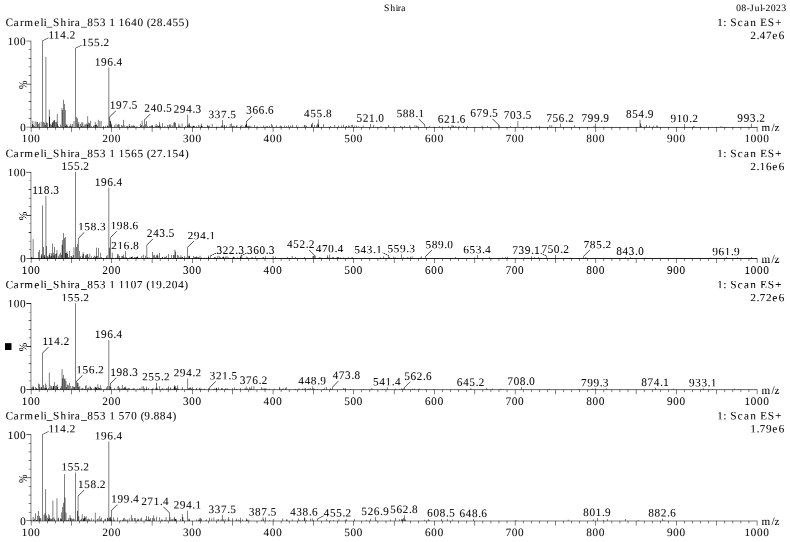

Figure S73. ^{1}H NMR Spectrum of Anabaenopeptin A (**7**) in DMSO-d_6

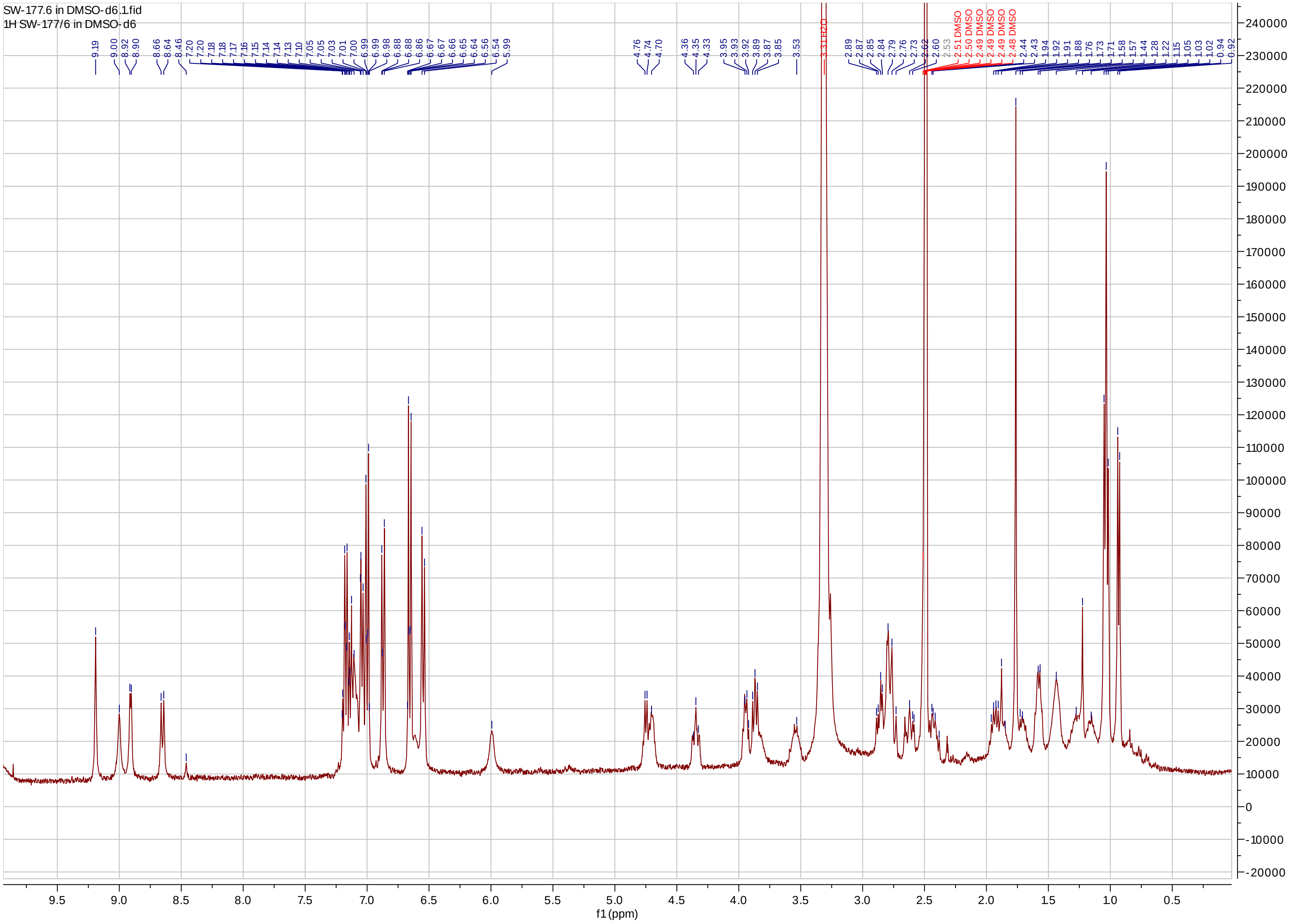

Figure S74. ^{13}C NMR Spectrum of Anabaenopeptin A (**7**) in DMSO-d_6

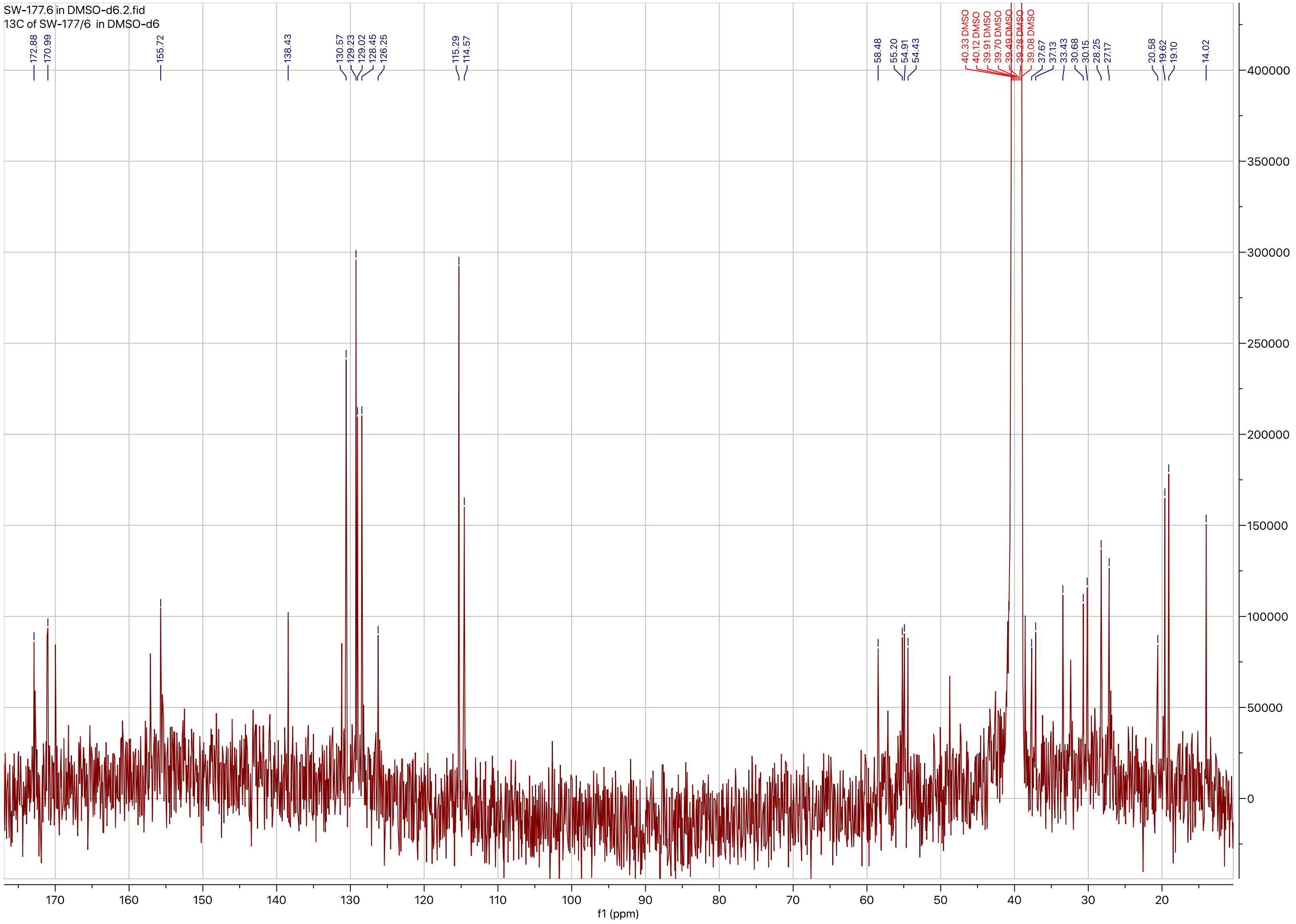

Figure S75. Negative HR ESI MS of Anabaenopeptin A (**7**)

Single Mass Analysis
Tolerance = 3.0 PPM / DBE: min = -1.5, max = 50.0
Element prediction: Off
Number of isotope peaks used for i-FIT = 3
Monoisotopic Mass, Even Electron Ions
479 formula(e) evaluated with 2 results within limits (all results (up to 1000) for each mass)
Elements Used:

Mass	Calc. Mass	mDa	PPM	DBE	Formula	i-FIT	i-FIT Norm	Fit Conf %	C	H	N	O
842.4099	842.4102	-0.3	-0.4	25.5	C45 H52 N11 O6	543.4	1.463	23.14	45	52	11	6
	842.4089	1.0	1.2	20.5	C44 H56 N7 O10	542.2	0.263	76.86	44	56	7	10

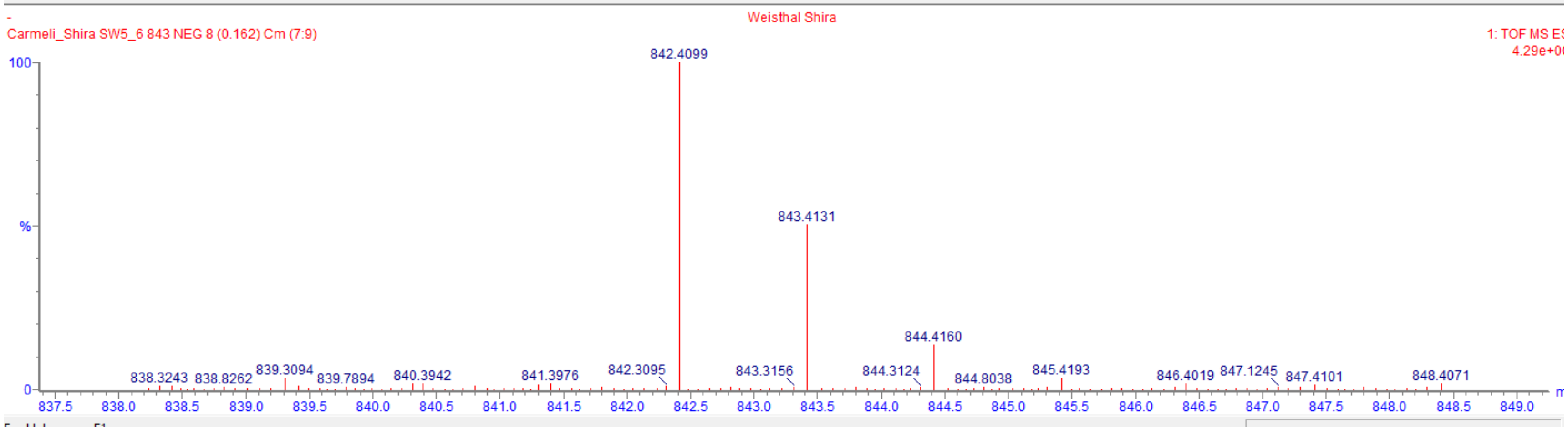

Figure S76. Positive HR ESI MS/MS Spectrum of Anabaenopeptin A (**7**)

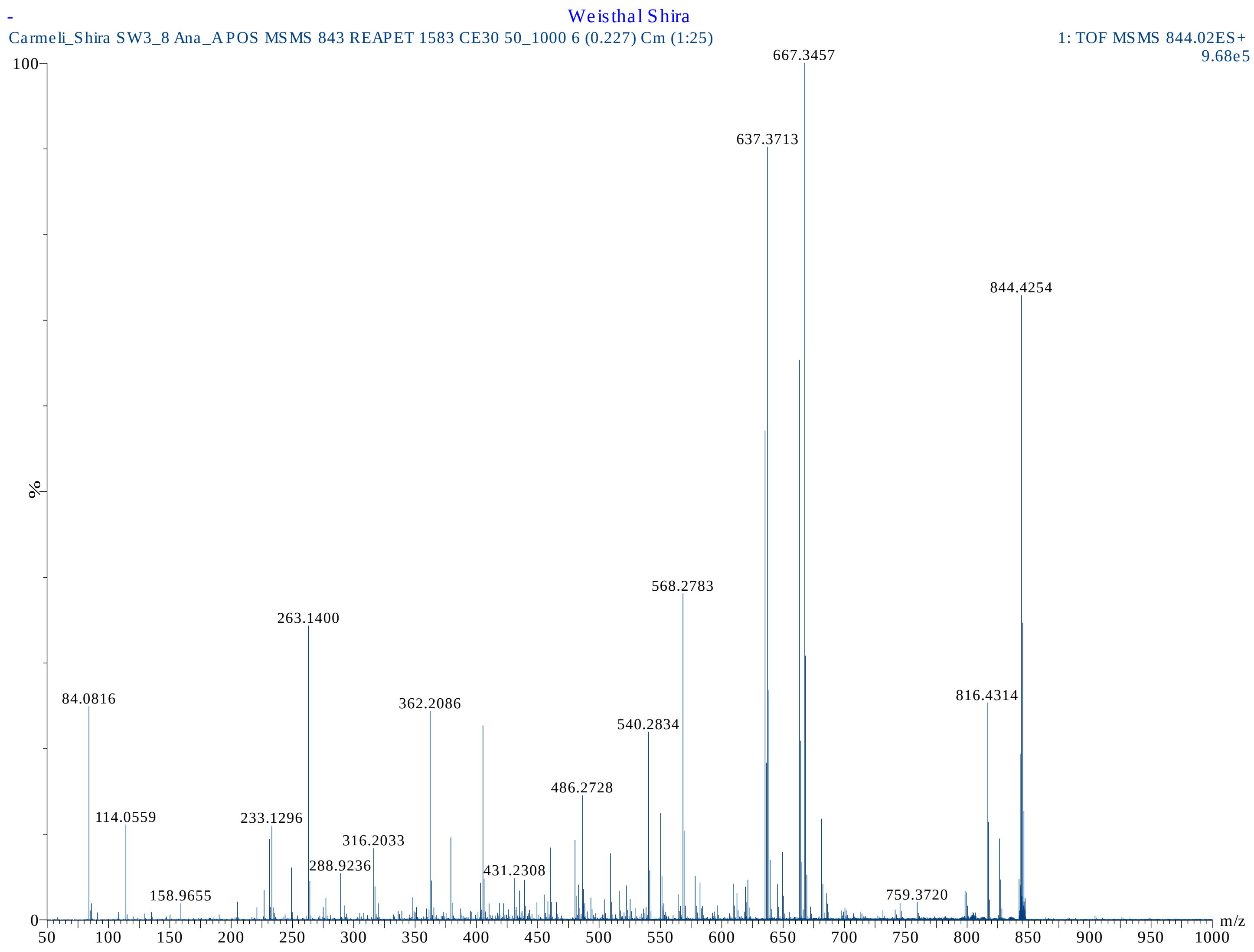

Figure S77. Scheme of Fragmentation in the Positive HR ESI MS/MS Spectrum of Anabaenopeptin A (**7**)

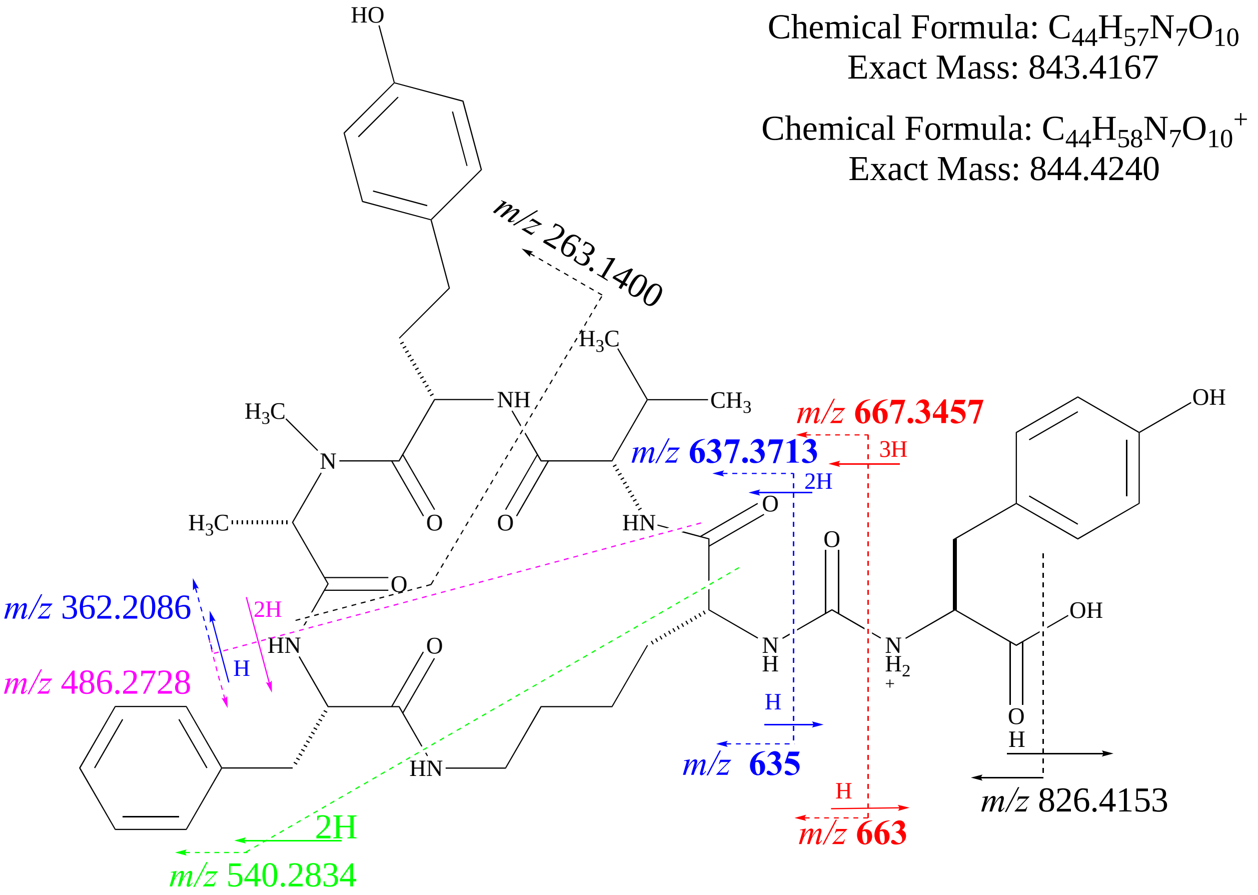

Figure S78. LCMS Traces of Marfey's Method Amino Acids Analysis of Anabaenopeptin A (**7**)

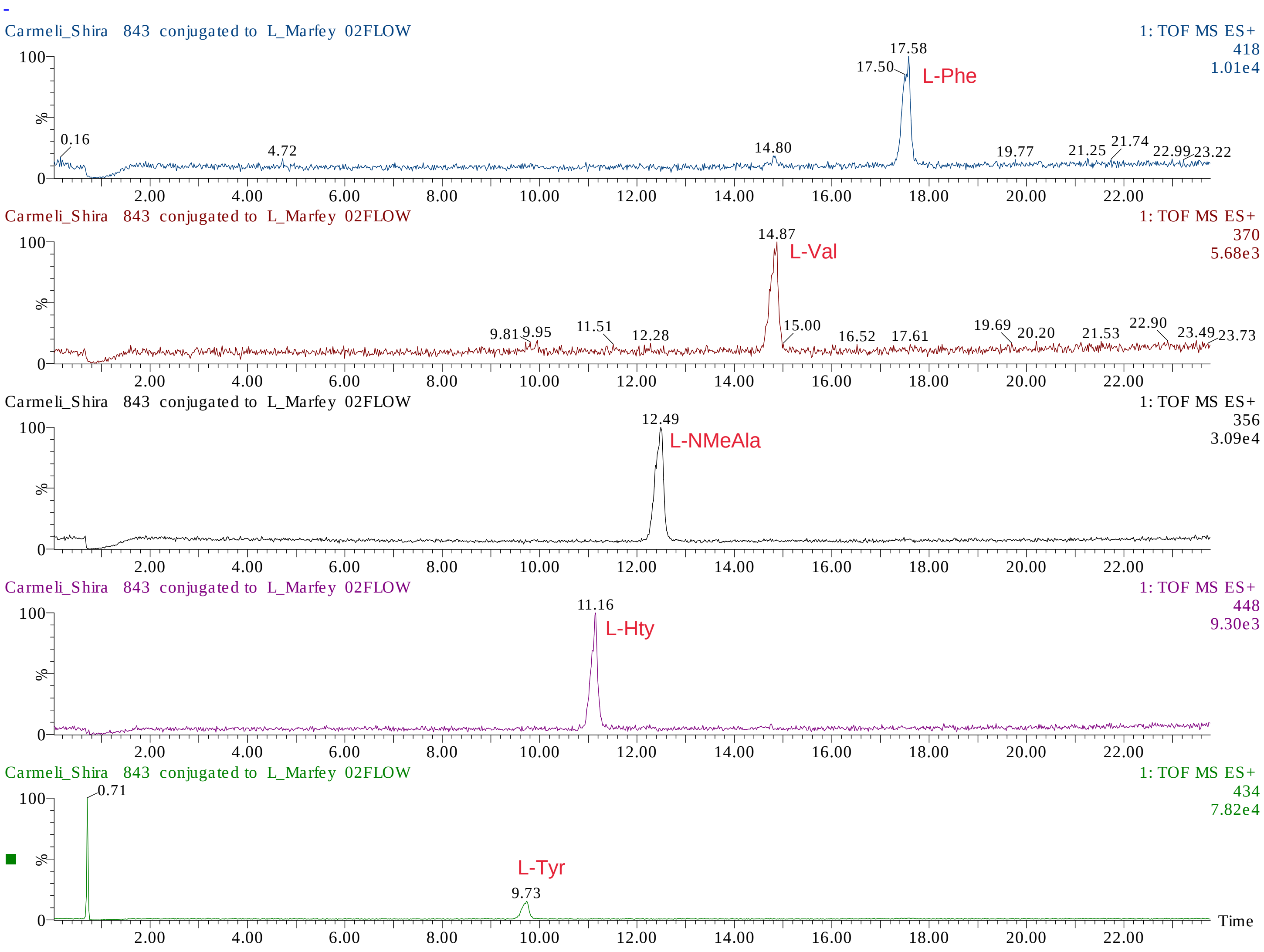

Figure S79. LCMS Chromatogram and Mass spectrum of Anabaenopeptin A (**7**)

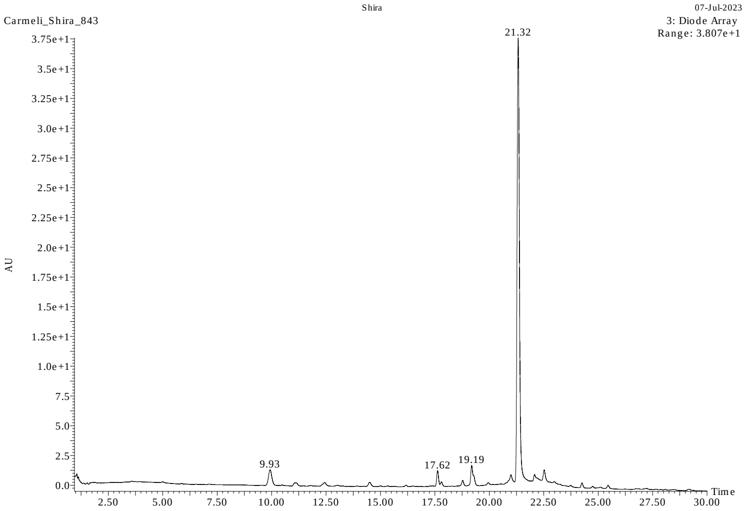

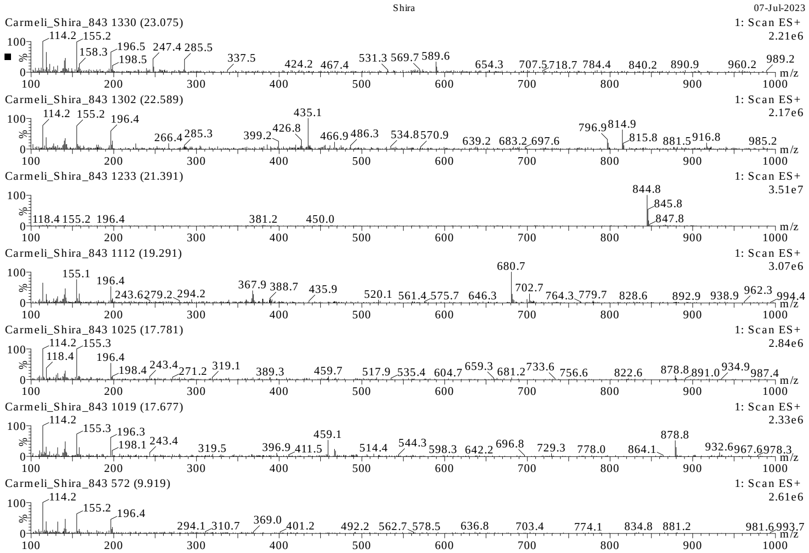

Figure S80. ^{1}H NMR Spectrum of Anabaenopeptin B (**8**) in DMSO-d_6

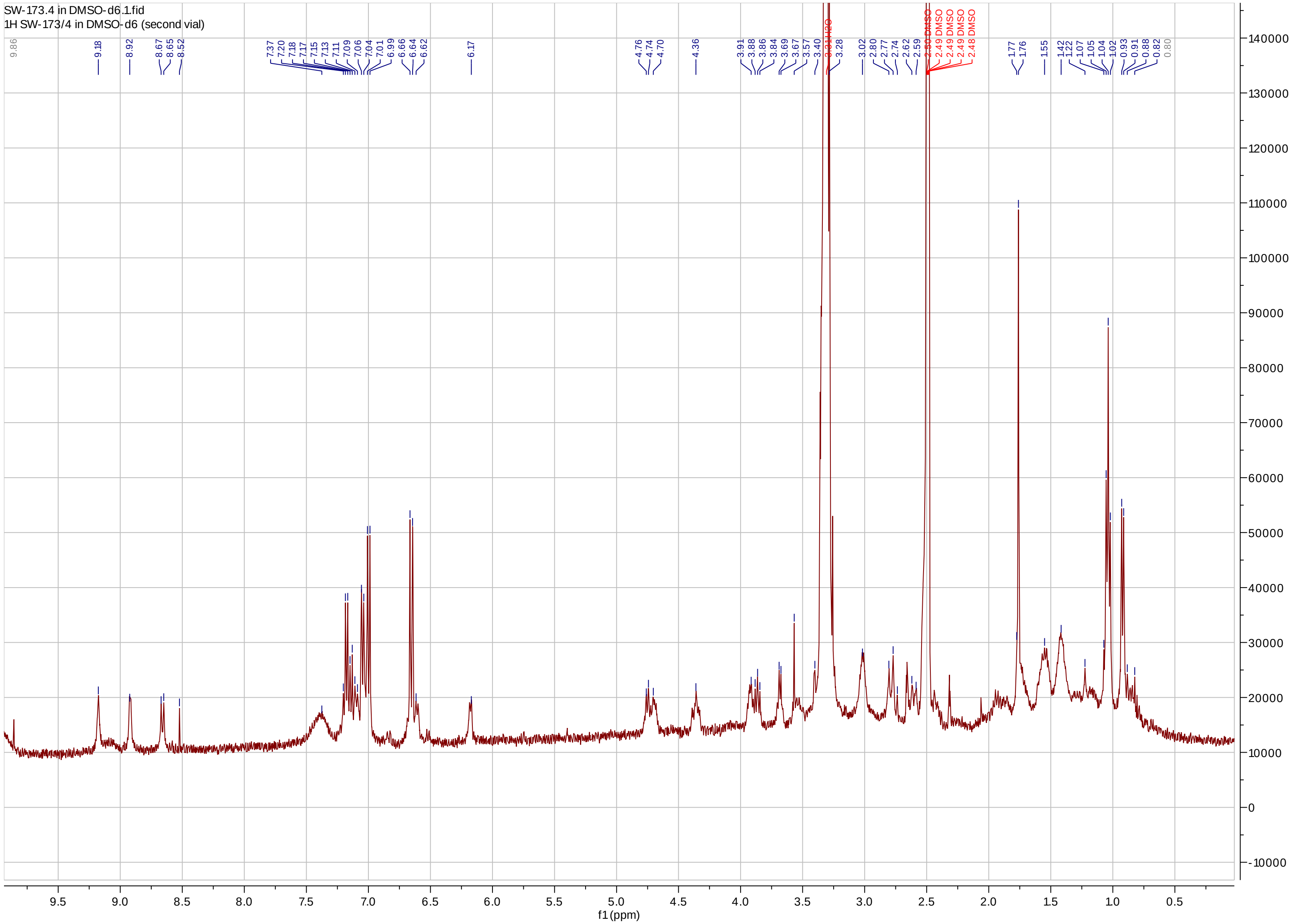

Figure S81. ^{13}C NMR Spectrum of Anabaenopeptin B (**8**) in DMSO-d_6

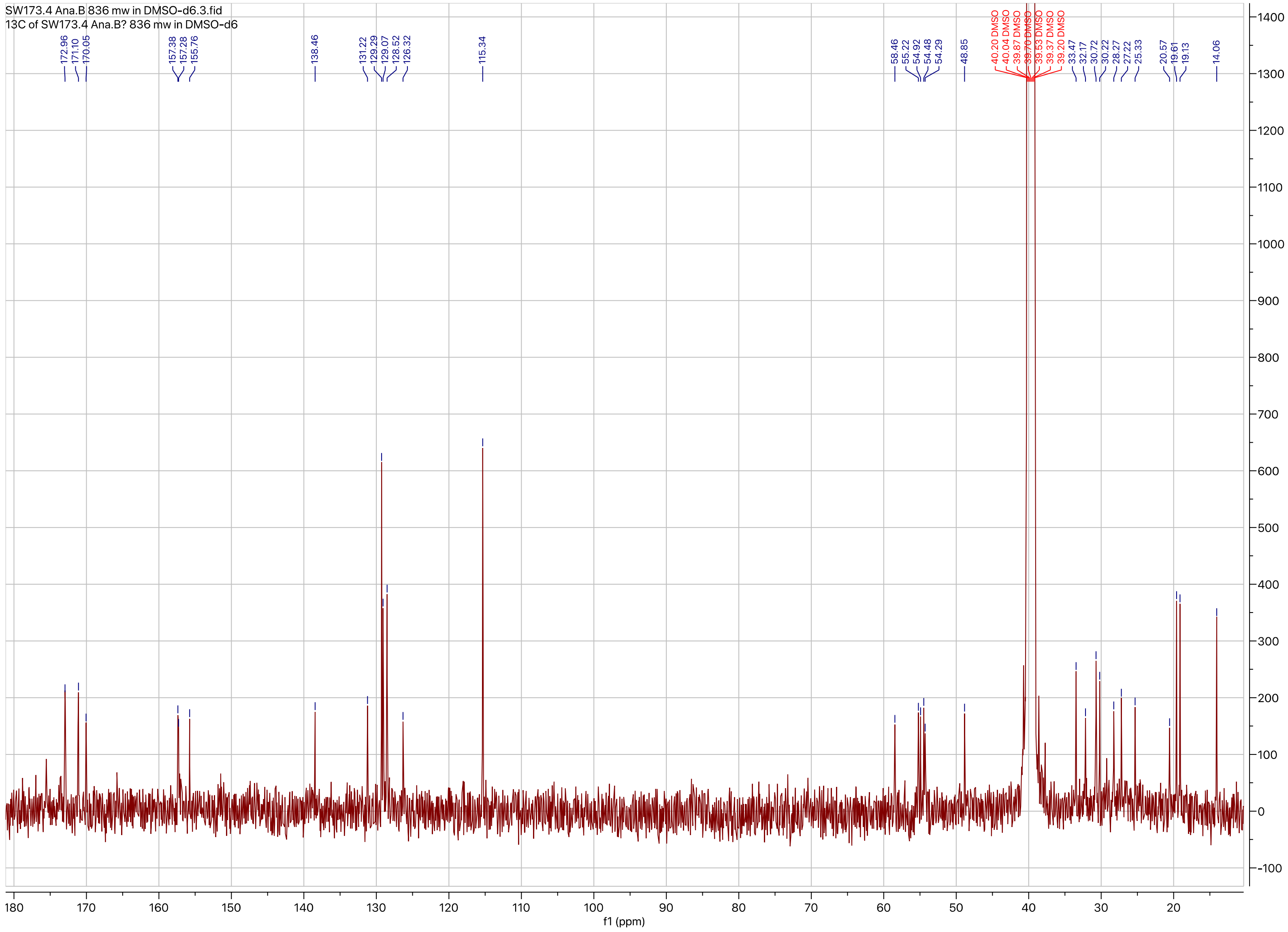

Figure S82. Positive HR ESI MS of Anabaenopeptin B (**8**)

Single Mass Analysis
Tolerance = 10.0 PPM / DBE: min = 0.0, max = 100.0
Element prediction: Off
Number of isotope peaks used for i-FIT = 3
Monoisotopic Mass, Odd and Even Electron Ions
248 formula(e) evaluated with 6 results within limits (all results (up to 1000) for each mass)
Elements Used:

Mass	Calc. Mass	mDa	PPM	DBE	Formula	i-FIT	i-FIT Norm	Fit Conf %	C	H	N	O	Na
837.4614	837.4612	0.2	0.2	13.0	C41 H64 N7 O10 Na	700.4	1.337	26.26	41	64	7	10	1
	837.4623	-0.9	-1.1	16.5	C41 H61 N10 O9	700.7	1.610	19.99	41	61	10	9	
	837.4599	1.5	1.8	13.5	C39 H62 N10 O9 Na	700.1	1.002	36.70	39	62	10	9	1
	837.4636	-2.2	-2.6	16.0	C43 H63 N7 O10	701.5	2.404	9.04	43	63	7	10	
	837.4639	-2.5	-3.0	17.5	C44 H62 N8 O7 Na	701.7	2.572	7.64	44	62	8	7	1
	837.4537	7.7	9.2	21.0	C45 H59 N9 O7	704.7	5.577	0.38	45	59	9	7	

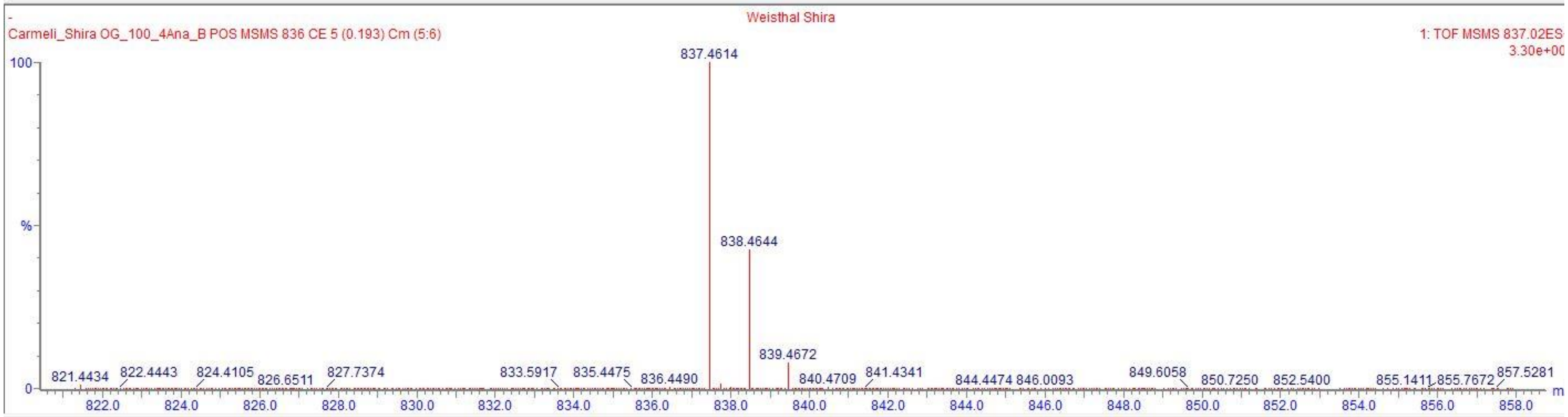

Figure S83. Positive HR ESI MS/MS Spectrum of Anabaenopeptin B (**8**)

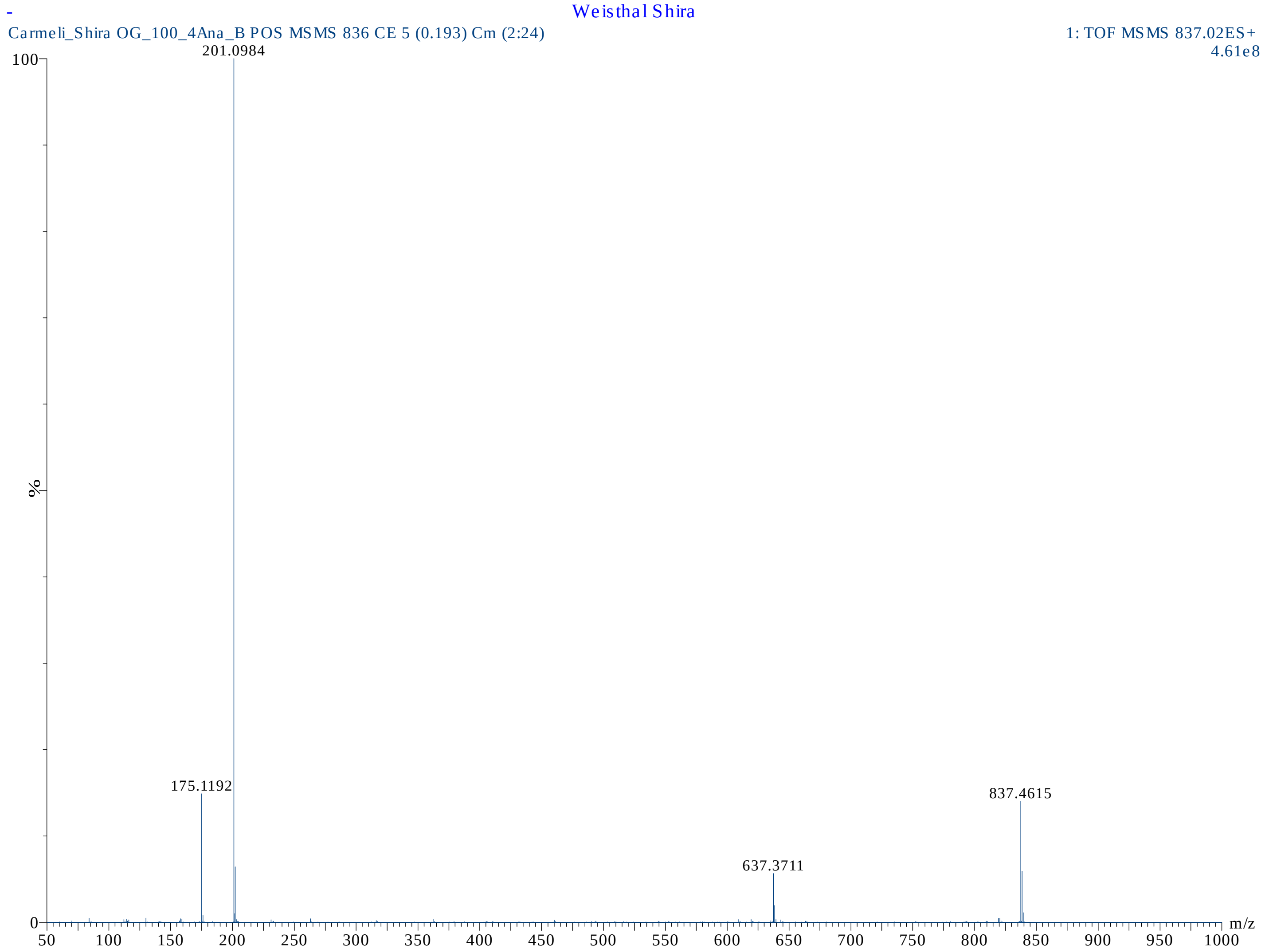

Figure S84. Scheme of Fragmentation in the Positive HR ESI MS/MS Spectrum of Anabaenopeptin B (**8**)

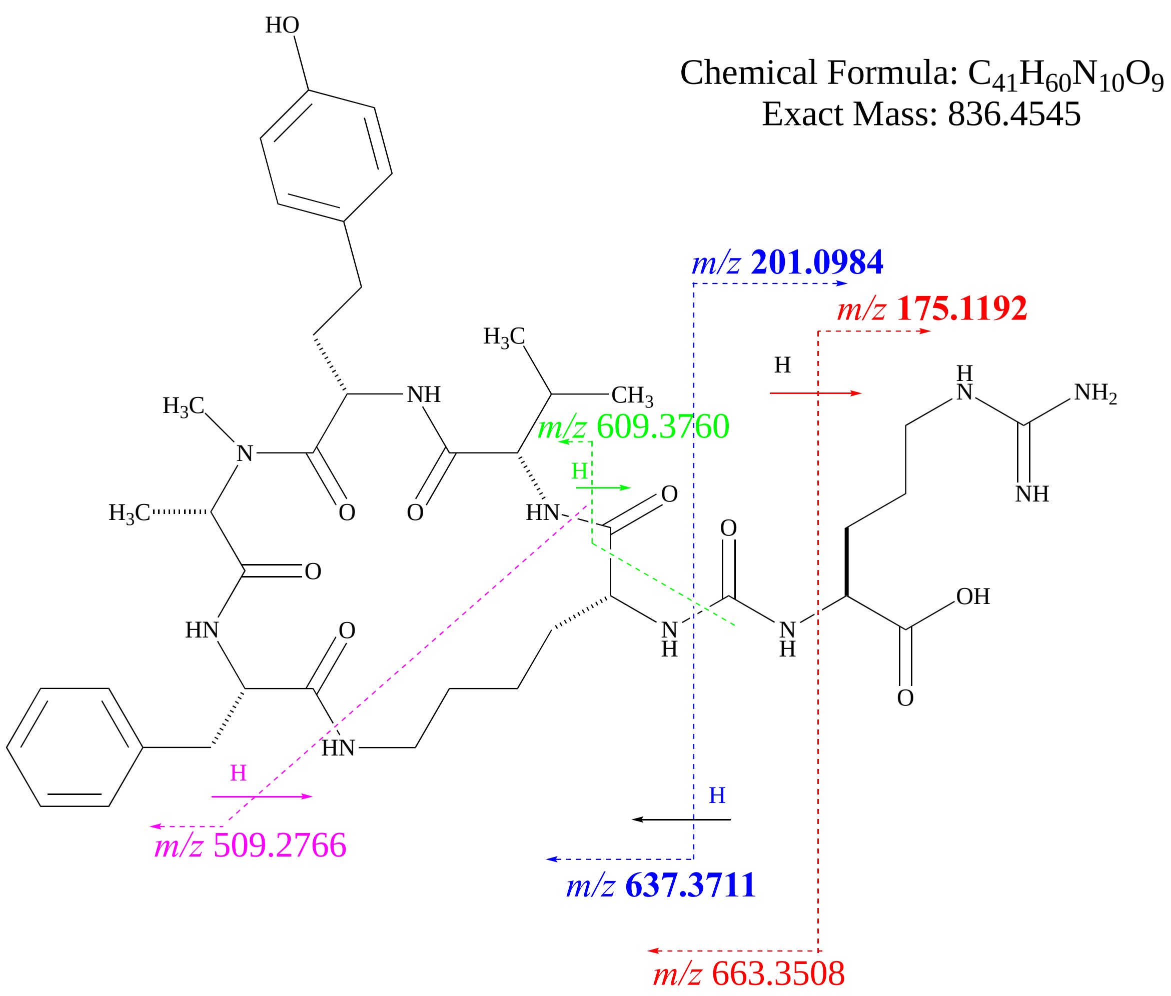

Figure S85. LCMS Traces of Marfey's Method Amino Acids Analysis of Anabaenopeptin B (**8**)

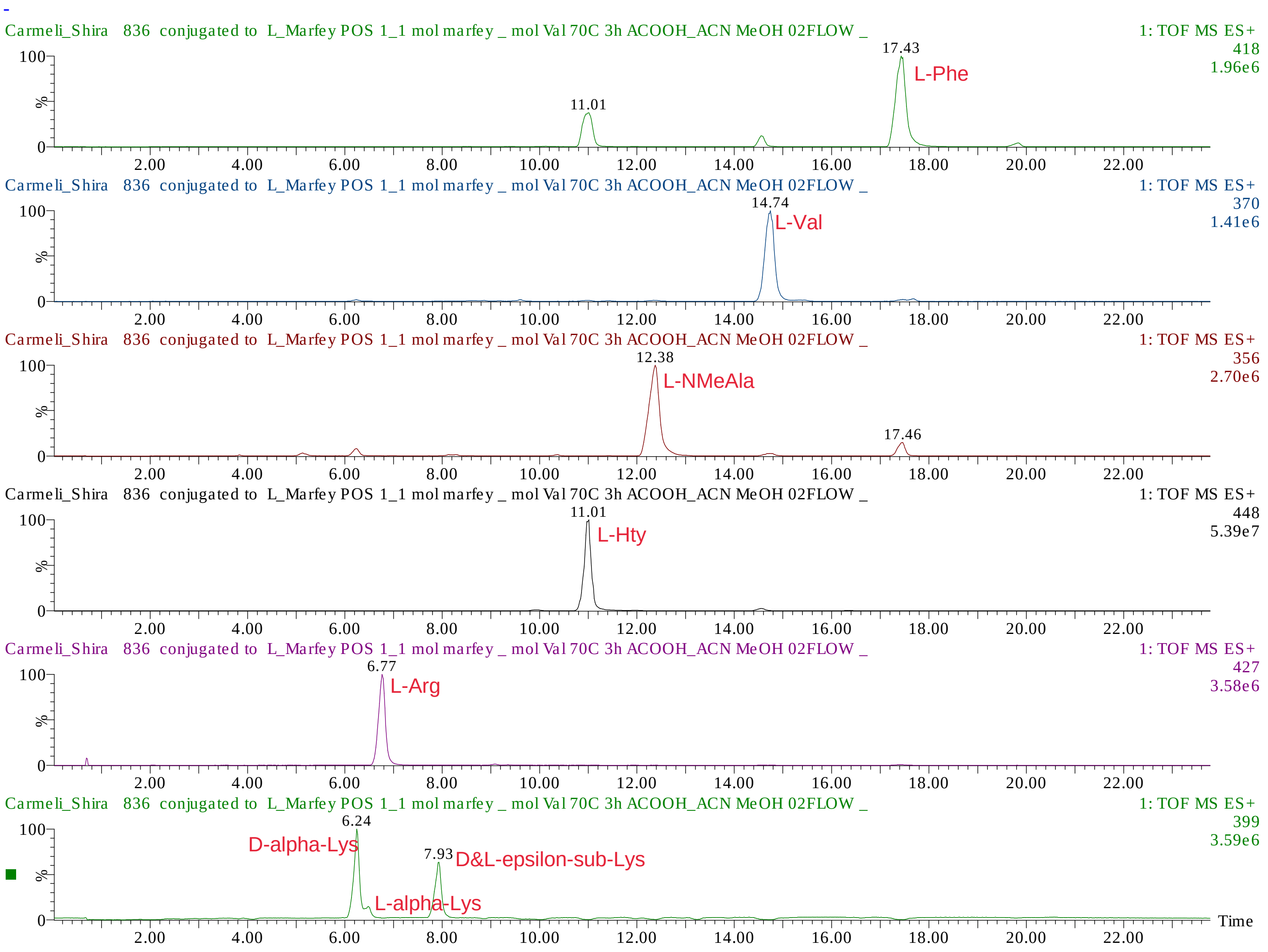

Figure S86. LCMS Chromatogram and Mass spectrum of Anabaenopeptin B (**8**)

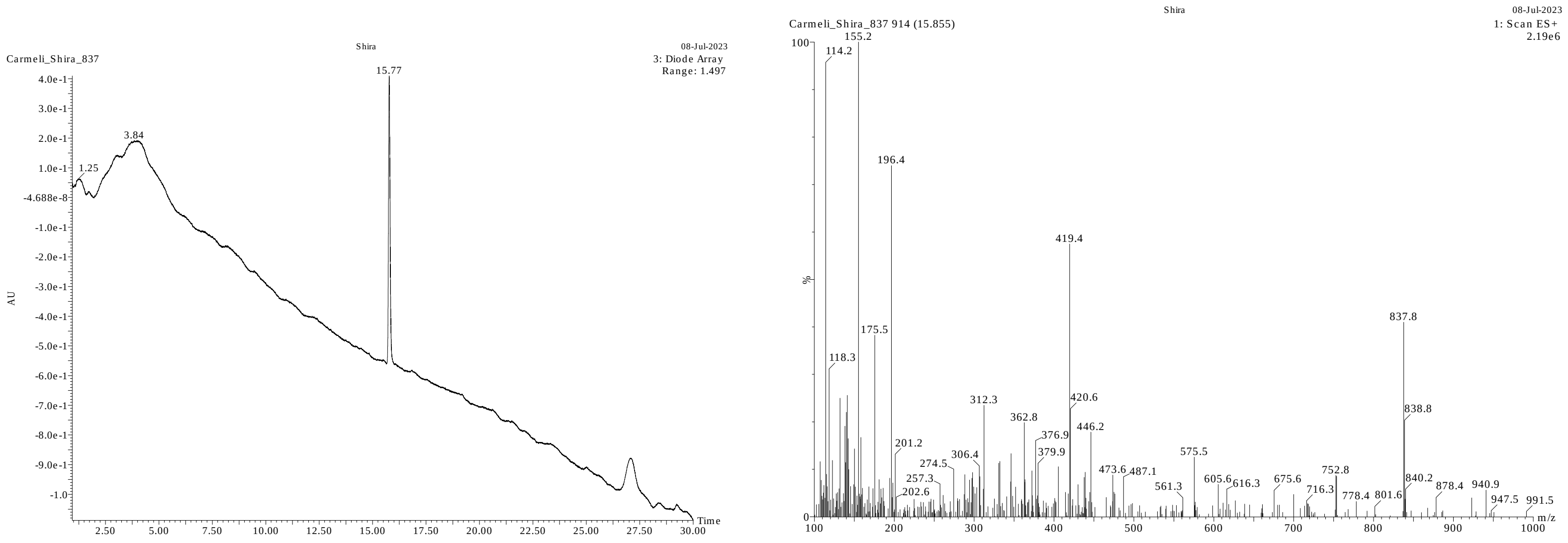

Figure S87. ^{1}H NMR Spectrum of Anabaenopeptin F (**9**) in DMSO-d_6

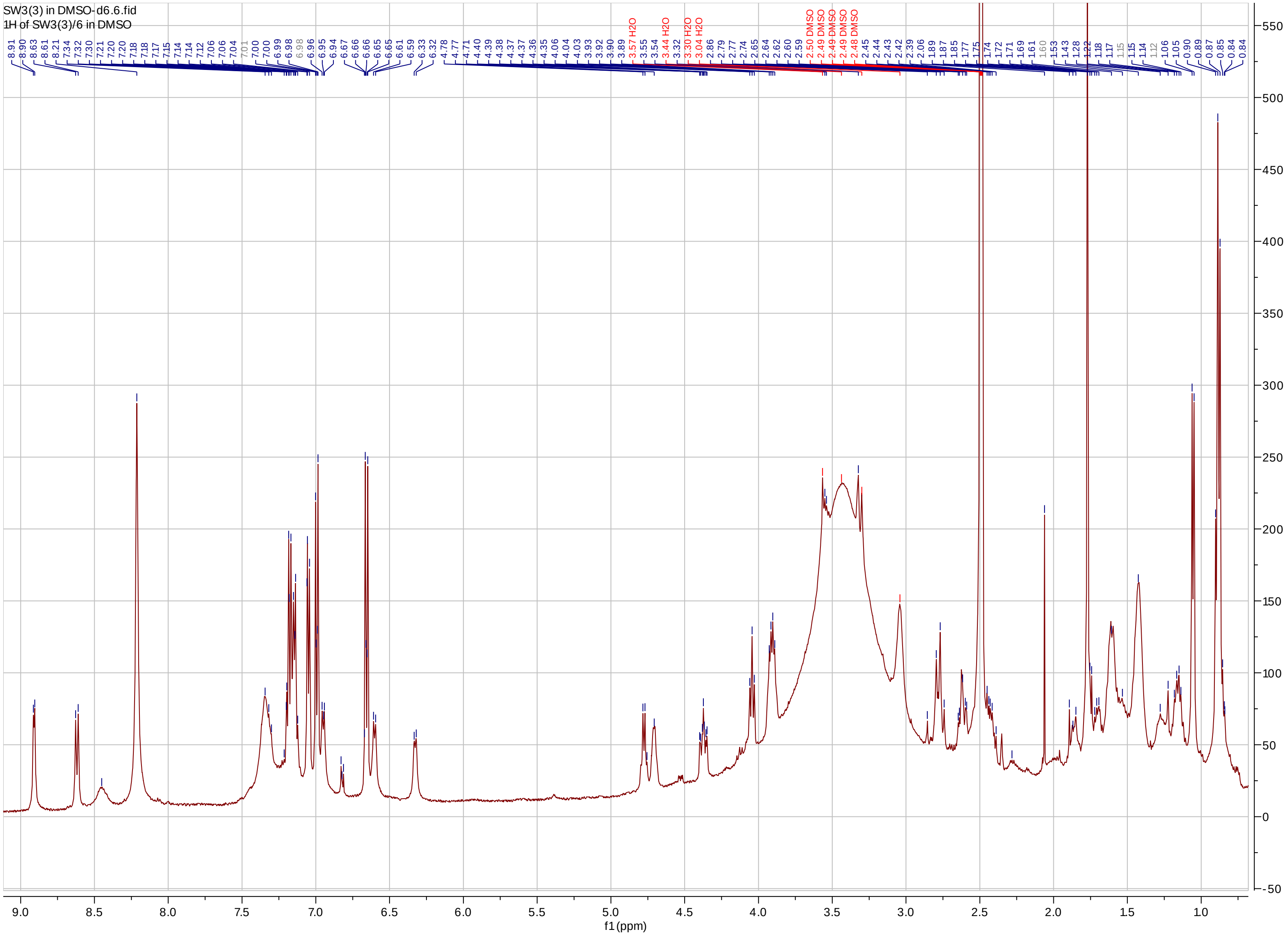

Figure S88. Positive HR ESI MS of Anabaenopeptin F (**9**)

Elemental Composition

File Edit View Process Help

Single Mass Analysis
Tolerance = 1.0 PPM / DBE: min = -1.5, max = 100.0
Element prediction: Off
Number of isotope peaks used for i-FIT = 3
Monoisotopic Mass, Odd and Even Electron Ions
839 formula(e) evaluated with 3 results within limits (up to 10 best isotopic matches for each mass)
Elements Used:

Mass	Calc. Mass	mDa	PPM	DBE	Formula	i-FIT	i-FIT Norm	Fit Conf %	C	H	N	O
851.4788	851.4779	0.9	1.1	16.5	C42 H63 N10 O9	1010.3	0.737	47.83	42	63	10	9
	851.4793	-0.5	-0.6	21.5	C43 H59 N14 O5	1010.6	1.056	34.79	43	59	14	5
	851.4793	-0.5	-0.6	16.0	C44 H65 N7 O10	1011.3	1.750	17.37	44	65	7	10

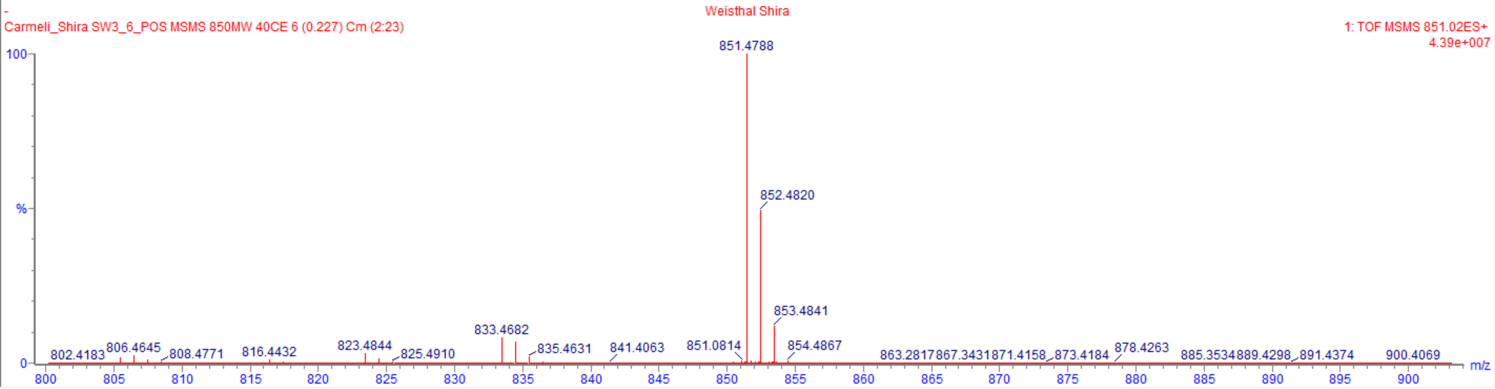

For Help, press F1

Figure S89. Positive HR ESI MS/MS Spectrum of Anabaenopeptin F (**9**)

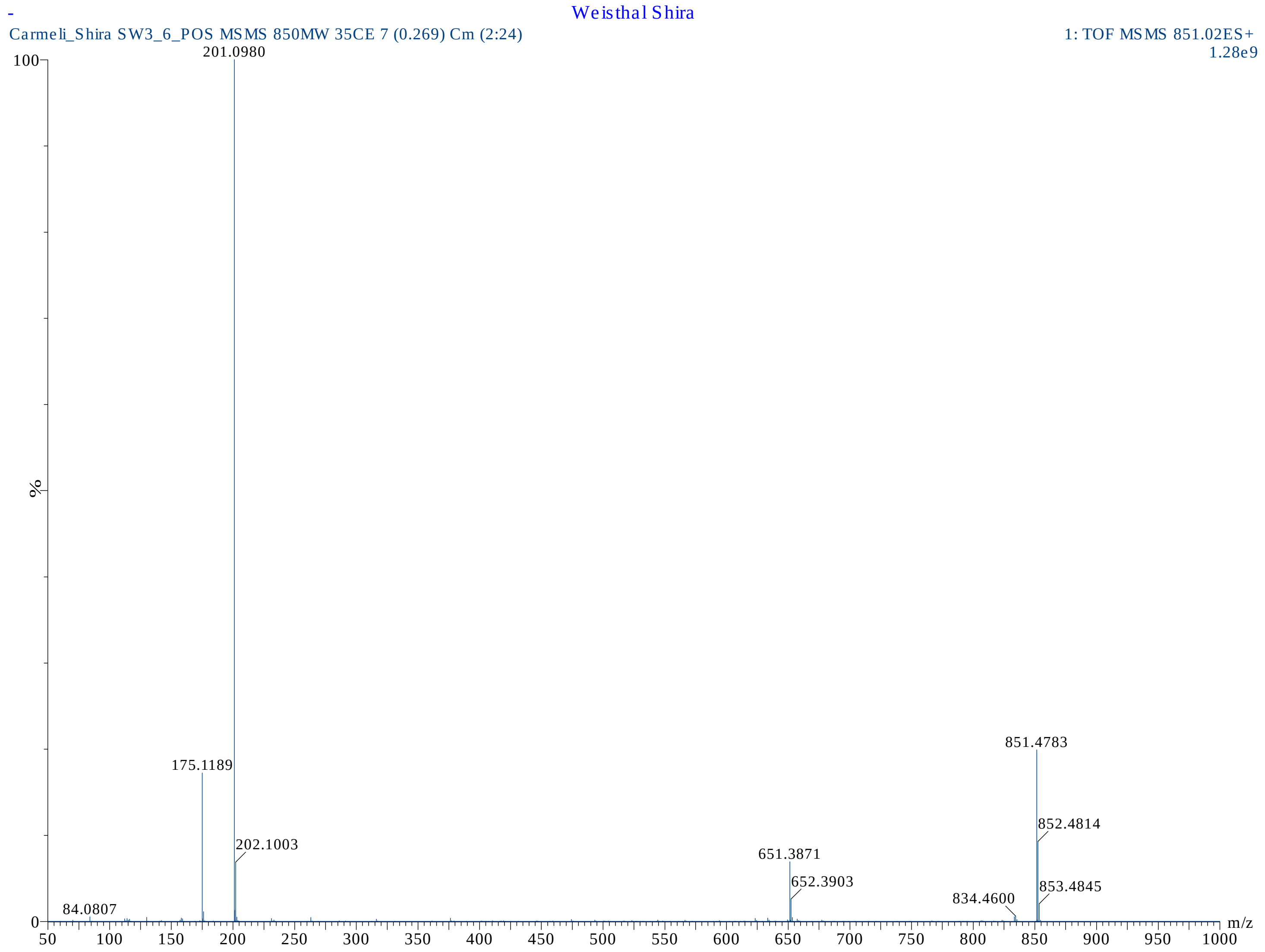

Figure S90. Scheme of Fragmentation in the Positive HR ESI MS/MS Spectrum of Anabaenopeptin F (**9**)

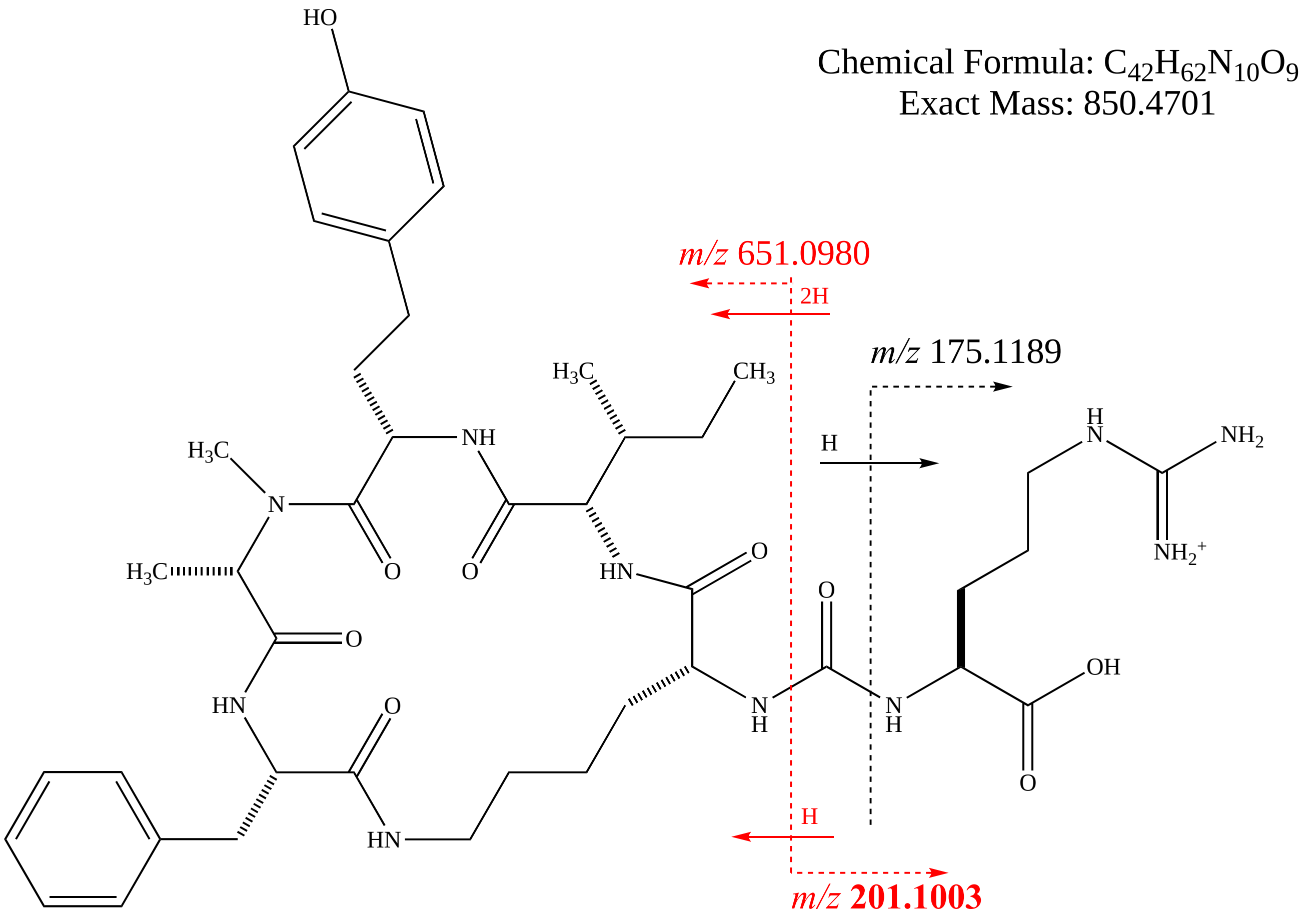

Figure S91. LCMS Traces of Marfey's Method Amino Acids Analysis of Anabaenopeptin F (**9**)

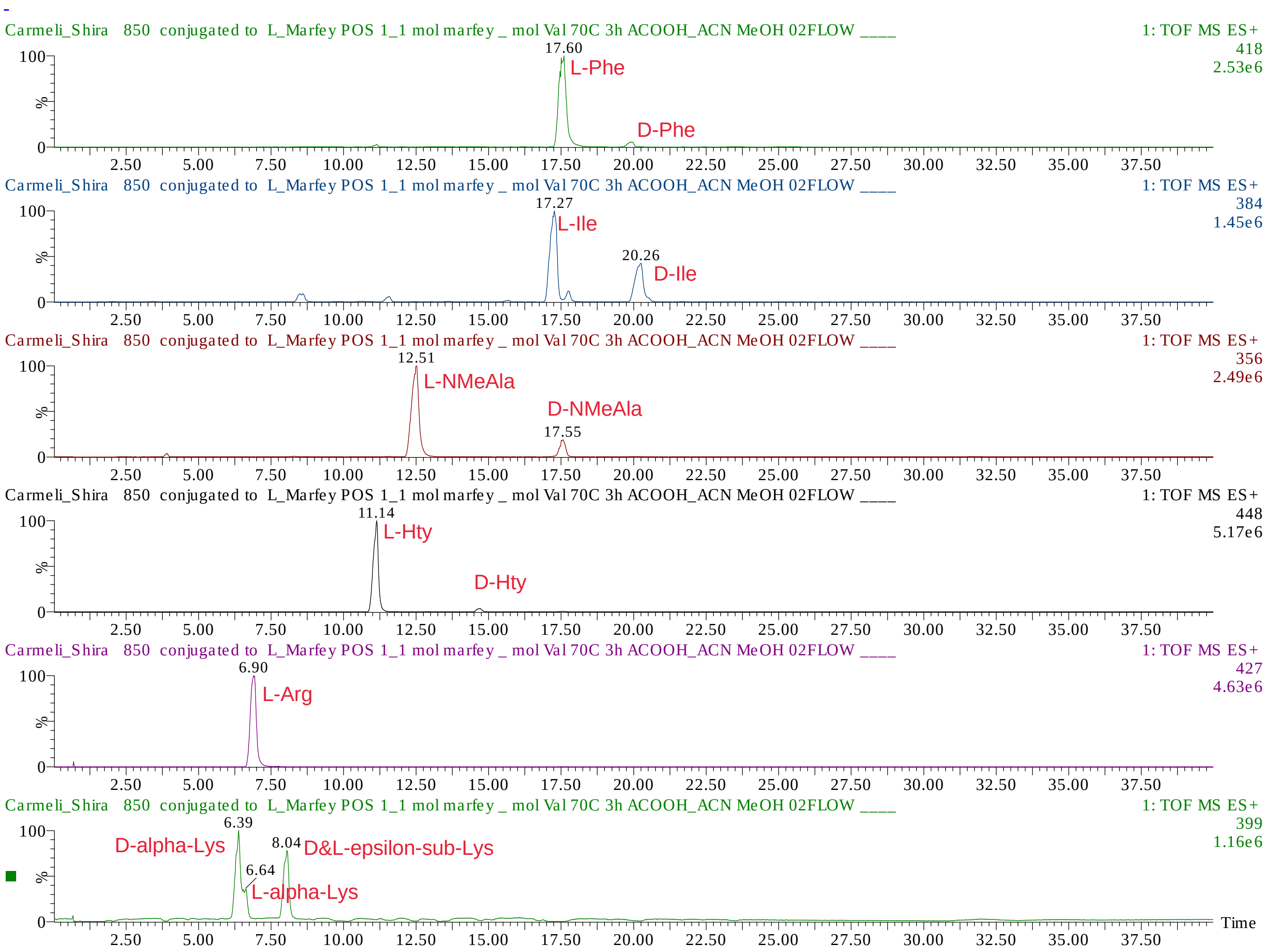

Figure S92. LCMS Chromatogram and Mass spectrum of Anabaenopeptin F (**9**)

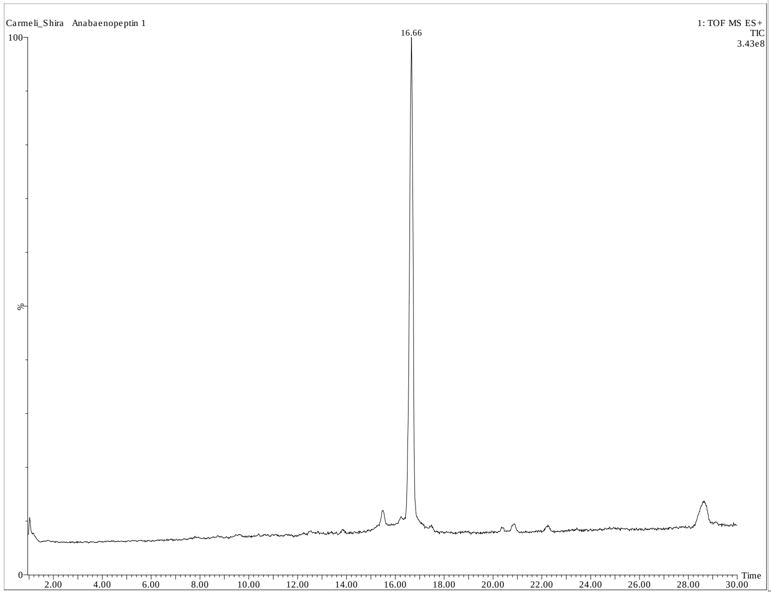

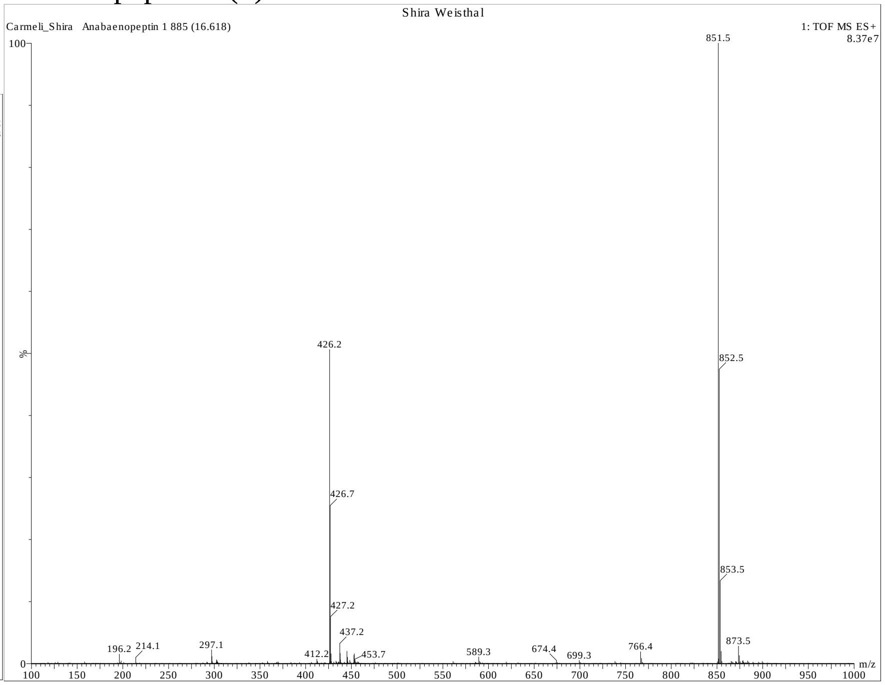

Figure S93. ^{1}H NMR Spectrum of Oscillamide Y (**10**) in DMSO-d_6

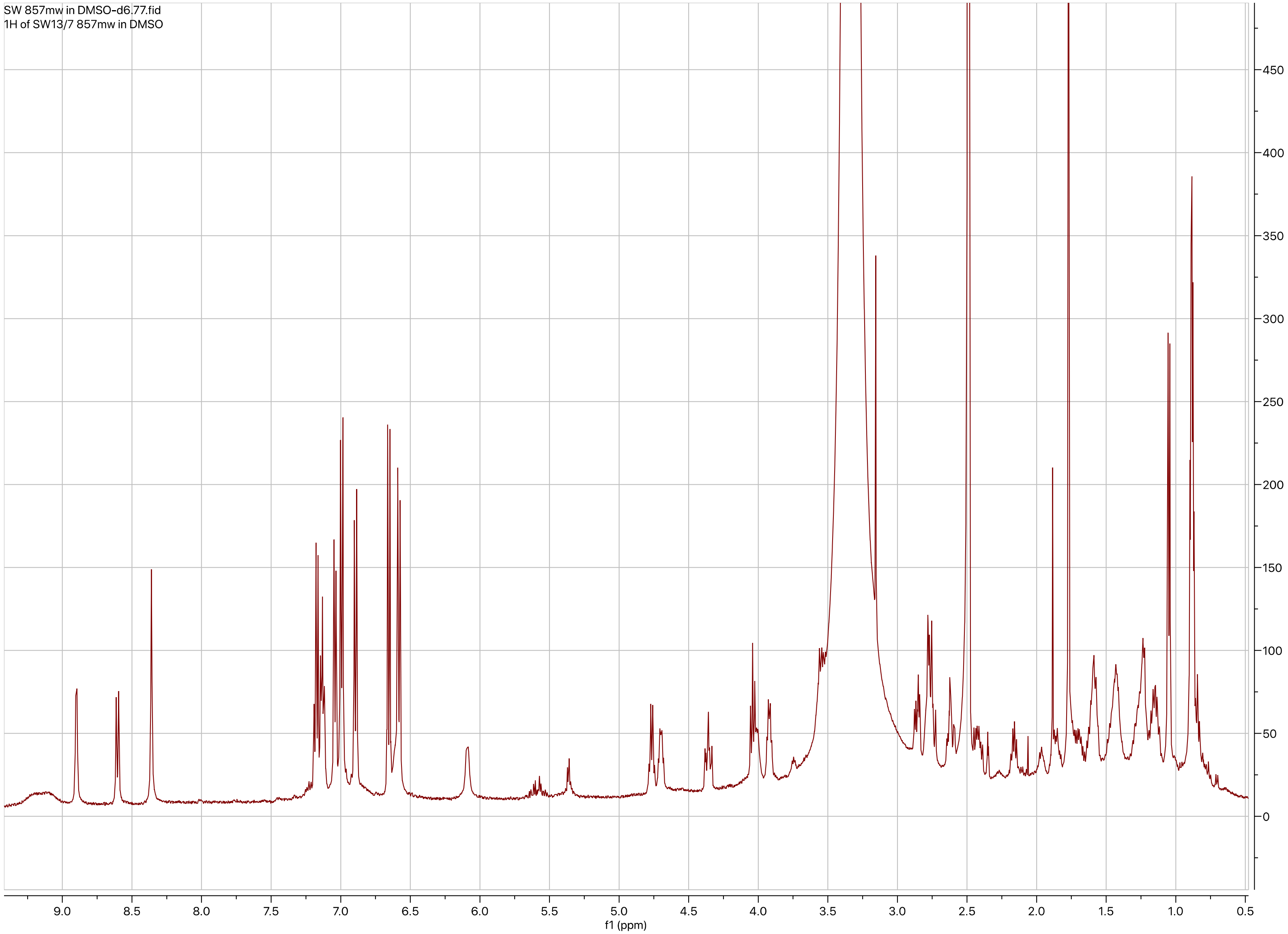

Figure S94. Negative HR ESI MS of Oscillamide Y (**10**)

Single Mass Analysis
Tolerance = 3.0 PPM / DBE: min = -1.5, max = 50.0
Element prediction: Off
Number of isotope peaks used for i-FIT = 3
Monoisotopic Mass, Odd and Even Electron Ions
645 formula(e) evaluated with 5 results within limits (all results (up to 1000) for each mass)
Elements Used:

Mass	Calc. Mass	mDa	PPM	DBE	Formula	i-FIT	i-FIT Norm	Fit Conf %	C	H	N	O
856.4249	856.4245	0.4	0.5	20.5	C45 H58 N7 O10	47.4	0.000	100.00	45	58	7	10
	856.4245	0.4	0.5	26.0	C44 H52 N14 O5	68.1	20.690	0.00	44	52	14	5
	856.4259	-1.0	-1.2	25.5	C46 H54 N11 O6	70.3	22.918	0.00	46	54	11	6
	856.4232	1.7	2.0	21.0	C43 H56 N10 O9	69.7	22.304	0.00	43	56	10	9
	856.4272	-2.3	-2.7	25.0	C48 H56 N8 O7	74.6	27.193	0.00	48	56	8	7

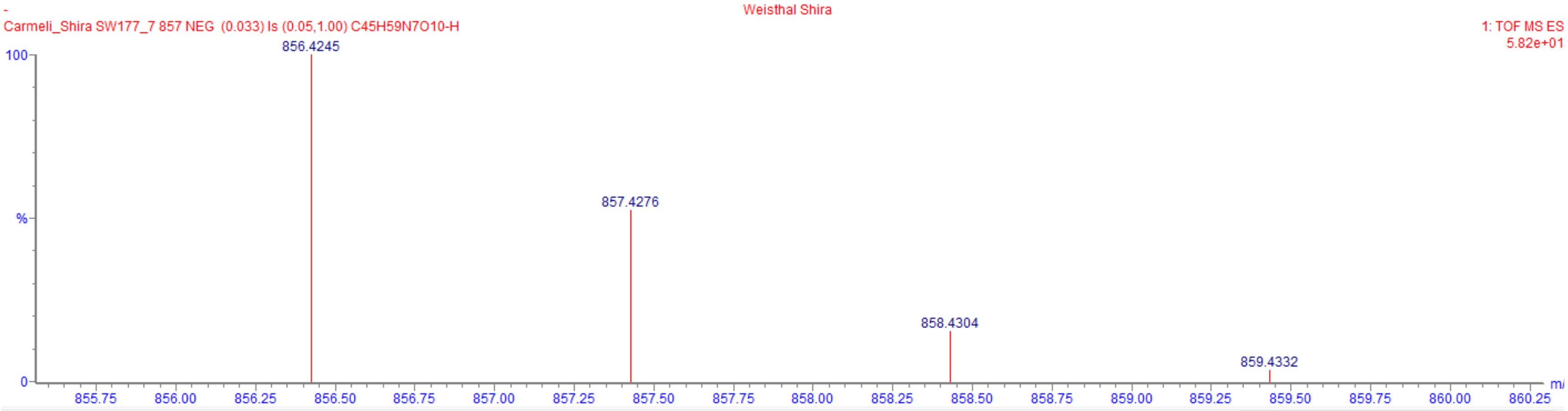

For Help, press F1

Figure S95. Positive HR ESI MS/MS Spectrum of Oscillamide Y (**10**)

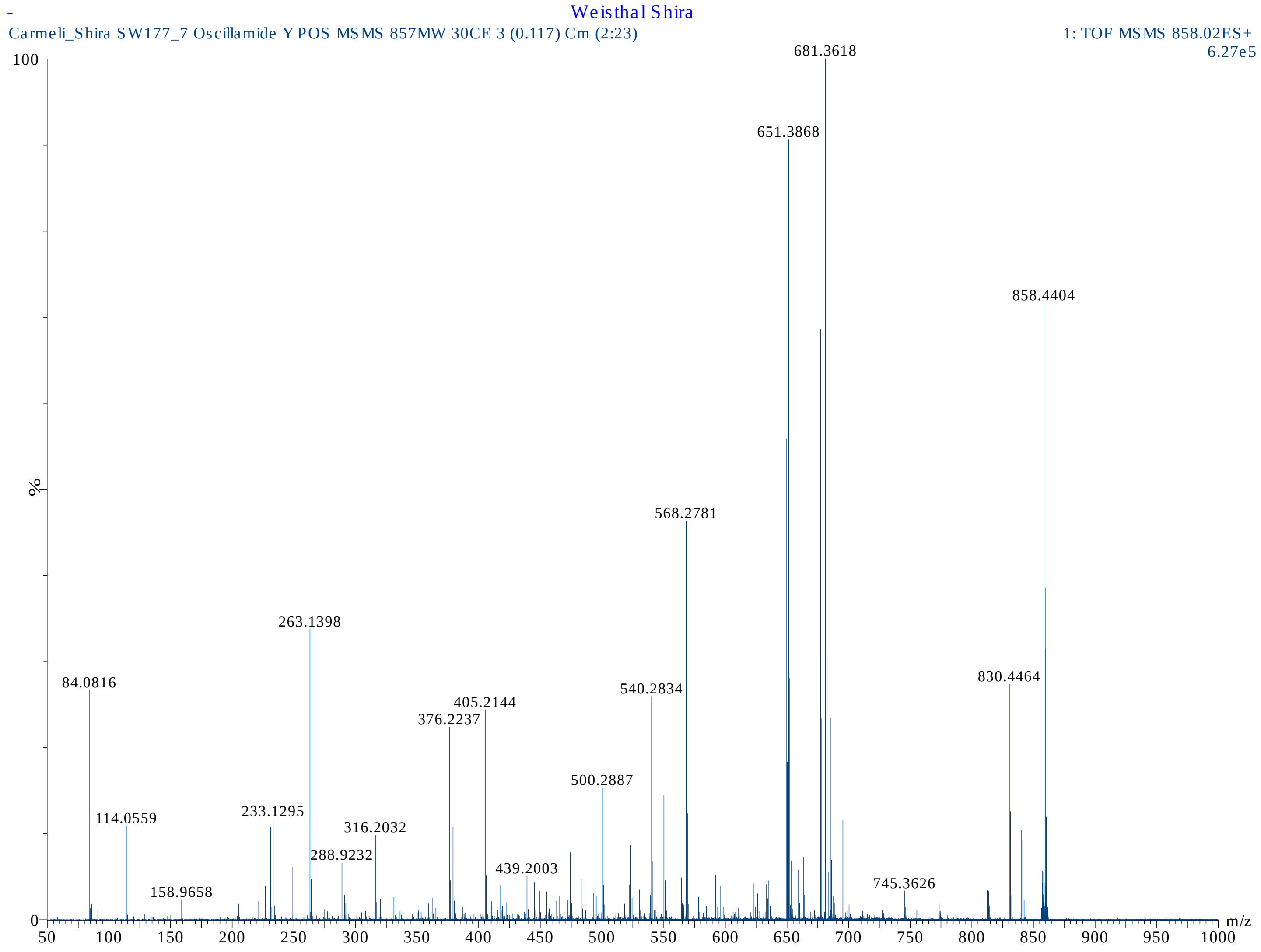

Figure S96. Scheme of Fragmentation in the Positive HR ESI MS/MS Spectrum of Oscillamide Y (**10**)

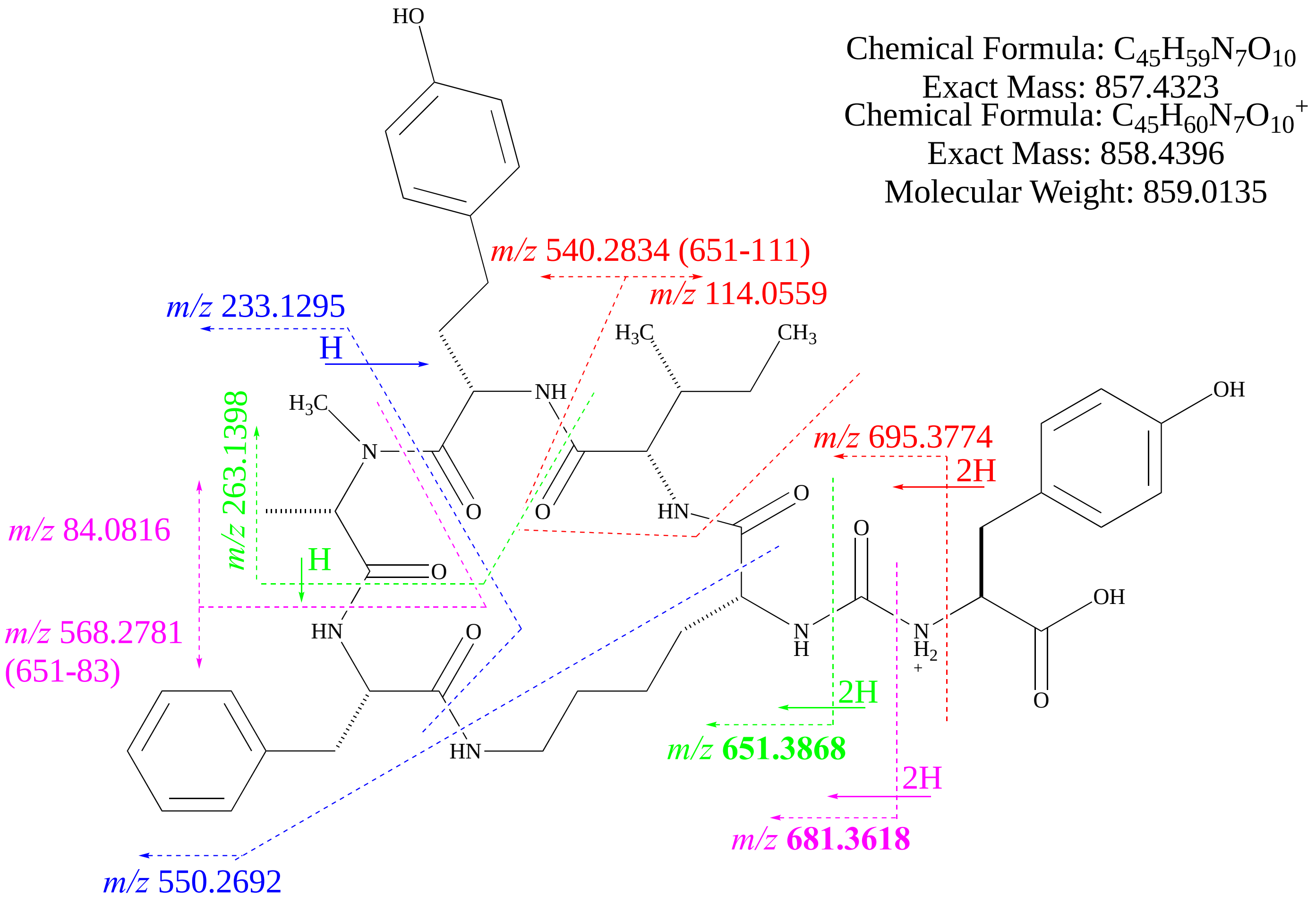

Figure S97. LCMS Traces of Marfey's Method Amino Acids Analysis of Oscillamide Y (**10**)

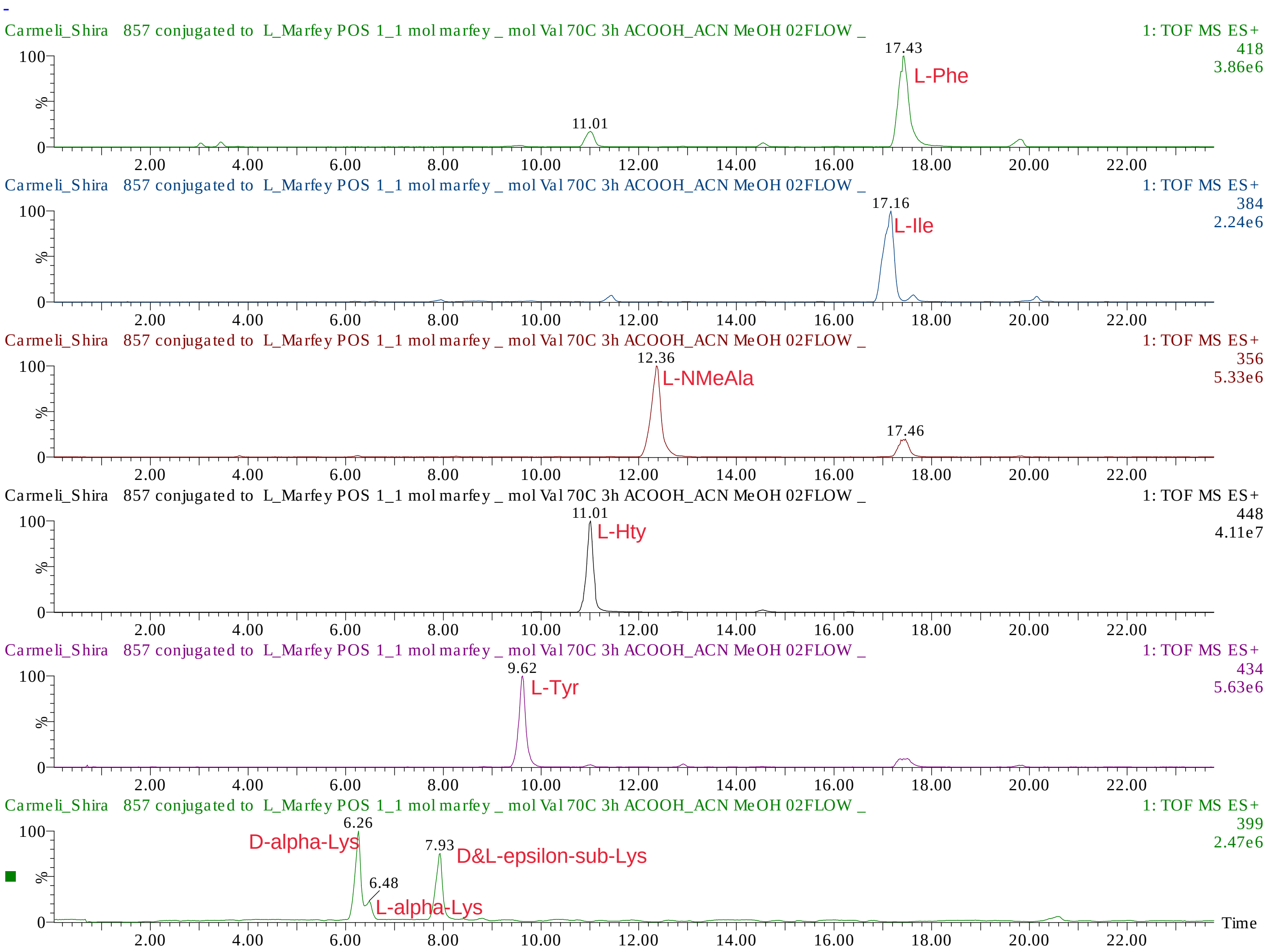

Figure S98. LCMS Chromatogram and Mass spectrum of Oscillamide Y (**10**)

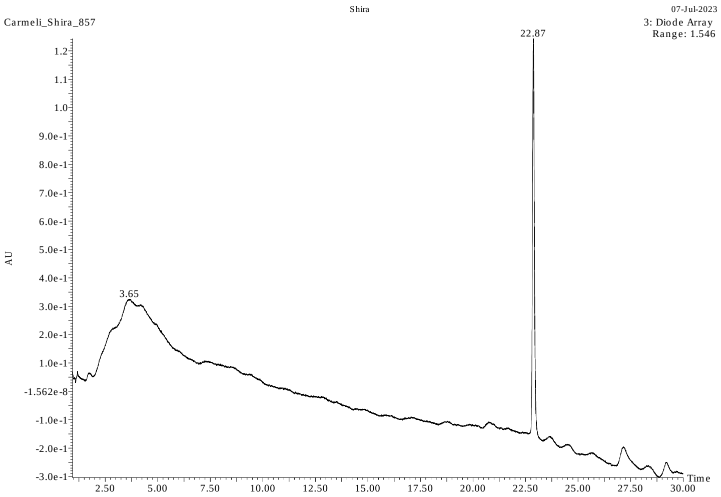

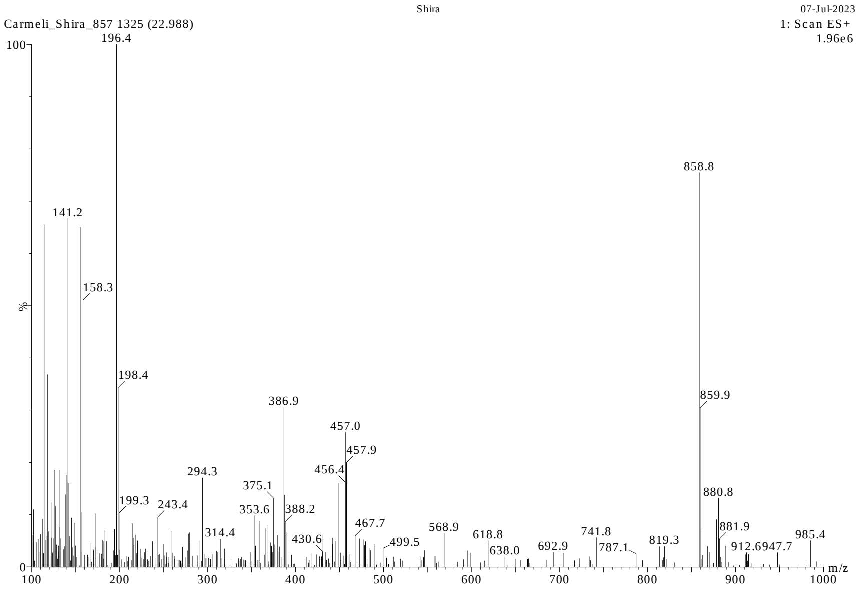

Supplement: Supplementary file 1 [file marinedrugs-21-00401-s001.zip › marinedrugs-2429752-supplementary.pdf]
